# Microwave-Assisted One-Pot Synthesis of Isothiouronium Salts: Experimental and DFT Insights into Silica-Promoted Cyclization toward Thiazolidinium and Thiazole Frameworks

**DOI:** 10.1021/acs.joc.5c01131

**Published:** 2025-09-05

**Authors:** Pablo Macías-Benítez, Andrés G. Algarra, F. Javier Moreno-Dorado, Francisco M. Guerra

**Affiliations:** † Departamento de Química Orgánica e Instituto de Biomoléculas (INBIO), Facultad de Ciencias, 16727 Universidad de Cádiz, Polígono Río San Pedro s/n, Puerto Real, Cádiz 11510, Spain; ‡ Departamento de Ciencia de los Materiales e Ingeniería Metalúrgica y Química Inorgánica e Instituto de Biomoléculas (INBIO), Facultad de Ciencias, Universidad de Cádiz, Polígono Río San Pedro s/n, Puerto Real, Cádiz 11510, Spain

## Abstract

Isothiouronium and thiazolidinium salts are sulfur-containing scaffolds commonly found in bioactive molecules. We report an expeditive one-pot, two-step procedure for the rapid synthesis of isothiouronium salts from carbon disulfide under microwave irradiation, allowing their isolation in less than 30 min and in good to excellent yields, without the need for a catalyst. When propargyl bromide is used as an alkylating agent, the corresponding isothiouronium salt undergoes an intramolecular cyclization during silica gel chromatography, affording a thiazolidinium salt. This rearrangement, not observed under the reaction conditions, was investigated via DFT calculations. Computations show that the uncatalyzed isomerization is not feasible but becomes accessible in the presence of silica gel, which acts as a proton shuttle. The rearrangement is shown to comprise two main steps that can take place in any order, i.e., [1,3] hydrogen shift and C–N bond formation. This leads to two alternative mechanisms with similar free energy barriers of ca. 18–19 kcal·mol^–1^, in both cases associated with the rate-determining C–N bond formation step.

## Graphical abstract

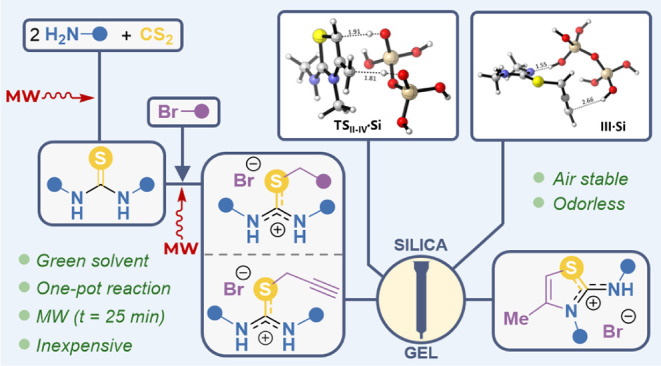

## Introduction

Isothiouronium salts are an important class of sulfur-containing compounds with diverse applications in both synthetic and biological chemistry. They can be found in various pharmaceuticals, such as difetur, used as an antihypotensive agent;^1^ levamisole hydrochloride as an anthelmintic;^2^ and xylazine hydrochloride as a sedative, analgesic, and muscle relaxant (Scheme 1A).^3^

**Scheme 1:**
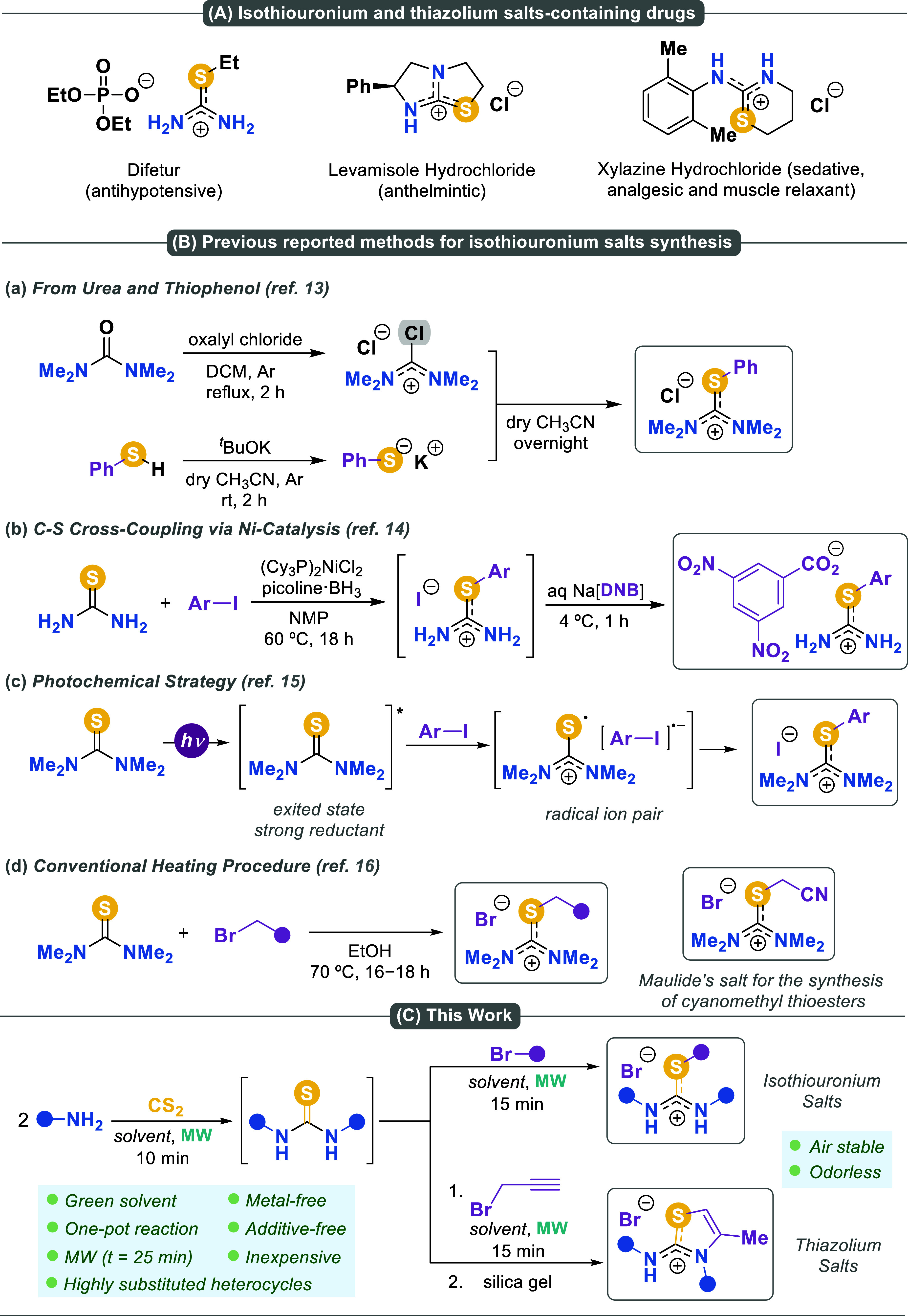
(A) Examples of Isothiouronium and Thiazolidinium Salts Containing Bioactive Molecules. (B) Previous Methods for Accessing Isothiouronium Salts. (C) This work: One-Pot Synthesis of Isothiouronium Salts and Highly Substituted Heterocycles via Microwave Radiation

Beyond their biological significance, isothiouronium salts have found widespread use in catalysis^4^ and materials science. They present a strong capacity for establishing hydrogen bonding,^4^ which makes them particularly useful in oxoanion recognition, an application exploited in the enantioselective discrimination of oxoanions by nuclear magnetic resonance^5^ and the development of fluorescence sensors.^6^ In synthetic chemistry, they have been employed as catalysts in hydrogenation reactions, often in conjunction with the Hantzsch ester,^7^ facilitating the reduction of 1,4-benzoxazines^8^ and 2-substituted quinolines.^9^

They are also involved in the reductive amination of aldehydes,^7^ the reduction of conjugated nitroalkenes,^10^ and proline-catalyzed aldol condensations.^11^

The synthesis of isothiouronium salts is traditionally based on the reaction of thiourea with alkyl or aryl halides.^12^ Classical methods involve the reaction of thiourea with alkyl bromides under prolonged heating (16–18 h). Thompson and coworkers described the formation of isothiouronium salts from urea and thiophenol, in which oxalyl chloride facilitates the substitution of the urea oxygen to form a chlorinated intermediate, which is then attacked by the deprotonated thiophenol (Scheme 1Ba).^13^ Although effective, this approach suffers from high base requirements and long reaction times. Ball and Magné developed a different approach, consisting in a nickel-catalyzed cross-coupling method to synthesize *S*-arylisothiouronium salts (Scheme 1Bb).^14^ This method provided an air-stable, odorless thiophenol surrogate that could be released *in situ* with a weak base, eliminating the need for direct handling of volatile and malodorous thiophenols. The *in situ* generation of isothiouronium salts as intermediates in the synthesis of thioesters under violet irradiation was demonstrated by Wu and Melchiorre (Scheme 1Bc).^15^ A remarkable example of the applicability of isothiouronium salts has been reported by Maulide and coworkers, who developed a method for the synthesis of cyanomethyl thioesters from carboxylic acids using bench-stable isothiouronium salts. The salt generates a cyanomethylthiolate unit that functions as a vector for sulfur transfer, enabling the formation of thioesters. This transformation avoids the need for unstable and odorous thiocarboxylic acids or thiols and shows the potential of isothiouronium salts as thiol surrogates (Scheme 1Bd).^16^

Ultimately, the sulfur source can be traced back to thiourea or isothiocyanate or, better yet, to carbon disulfide (CS_2_), the sulfur analogue of carbon dioxide. Among sulfur-containing compounds, CS_2_ stands out as an inexpensive, readily accessible, and versatile C_1_ synthon. Despite its reputation, carbon disulfide remains an attractive reagent due to its high reactivity, low cost, and ability to generate valuable intermediates when handled with proper protocols. In recent years, environmental concerns have motivated us to develop more efficient synthetic routes by streamlining reaction sequences, minimizing waste, reducing energy consumption, and shortening reaction times. Herein, we describe a concise two-step, one-pot synthesis of isothiouronium salts from carbon disulfide where thiourea acts as a nonisolated intermediate. Such a methodology allows for the isolation of isothiouronium salts from different alkylating agents, and microwave irradiation enables reaction completion within minutes. When propargyl bromide was employed as the electrophilic partner, the corresponding isothiouronium salt was first formed, which then cyclized to the thiazolidinium salt upon chromatographic separation on silica gel. Interestingly, Botta and coworkers had previously reported that treatment of thioureas with propargyl bromides in the presence of potassium carbonate triggers a domino alkylation-cyclization sequence that leads to 2-aminothiazoles.^17^ In this case, the presence of a base hampered the isolation of the isothiouronium salts, and those authors indicated that, in the absence of such a base, the reaction leads to complex mixtures. Building on this precedent, we sought to explore the mechanism of this transformation. We present a computational analysis of the cyclization process, examining both the intrinsic rearrangement and the influence of silica gel on the reaction outcome (Scheme 1C). Given the importance of thiazole scaffolds in medicinal chemistry—associated with antioxidant, analgesic, antifungal, neuroprotective, and antitumor properties—this reaction may prove useful for the synthesis of bioactive molecules.

## Results

The synthesis of isothiouronium salts commenced by optimizing the reaction conditions, aiming to identify those providing both high efficiency and environmental compatibility. Careful solvent selection is crucial for achieving an environmentally friendly and sustainable process. To this end, we prioritized solvents that meet green chemistry standards and used the Cumulative Energy Demand (CED) of solvent production—a measure of the total energy required to produce one kilogram of solvent—as a guiding metric (Figure 1).^18^

**Figure 1:**
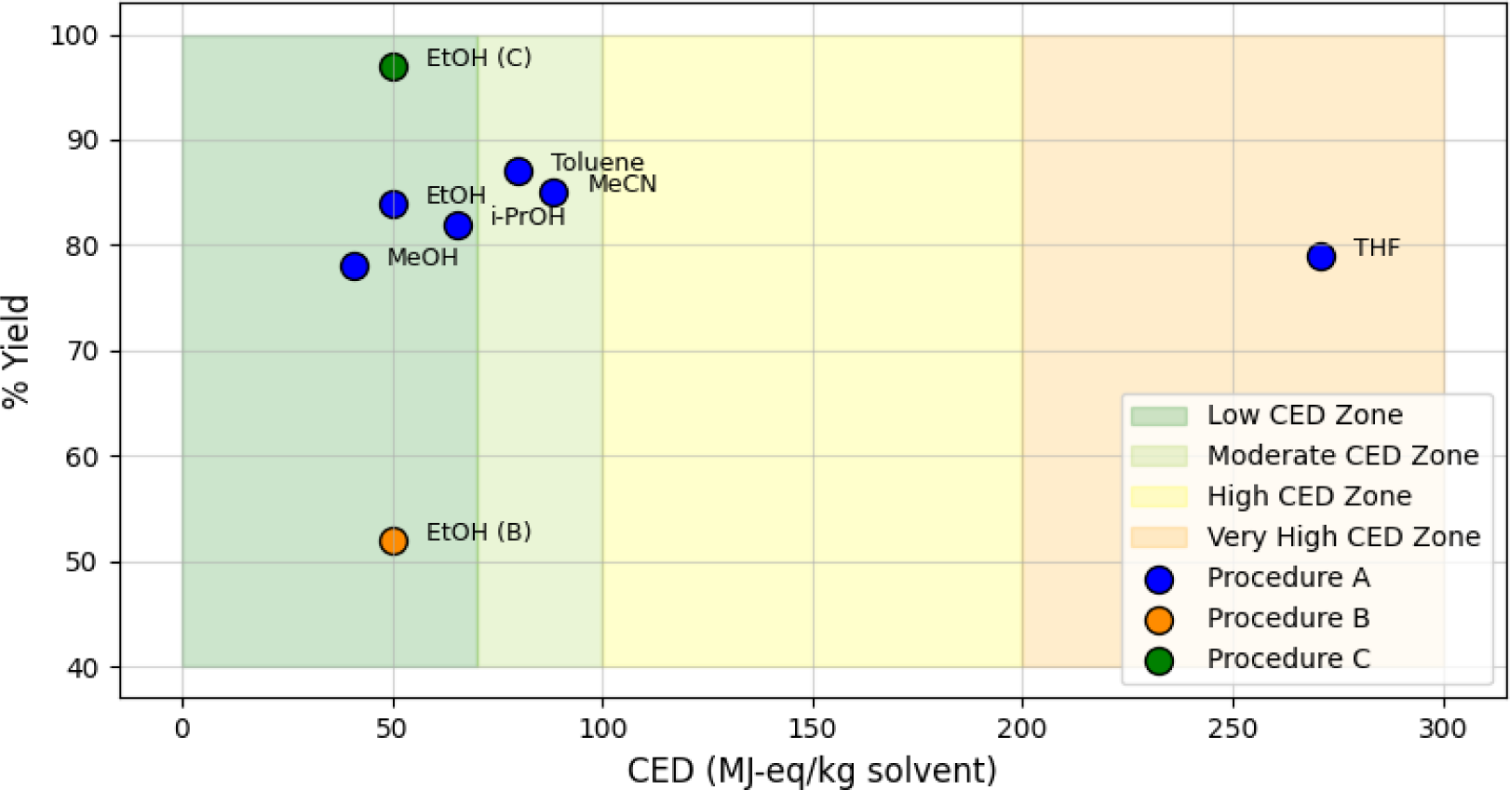
Relationship between the solvent cumulative energy demand (CED) and reaction yield for different solvents and procedures.

In order to investigate the effects of the solvents, we evaluated a number of solvents under conditions that included an initial irradiation step at 100 °C for 10 min, followed by the addition of benzyl bromide and a further 15 min of irradiation at the same temperature (Procedure A). As shown in Table 1, methanol (CED = 40.7 MJ-equiv per kg; entry 6) and ethanol (CED = 50.1 MJ-equiv per kg; entry 1), which were considered to be the greenest options based on CED, yielded 78% and 84%, respectively. Other solvents, including isopropanol (entry 7), toluene (entry 2), and acetonitrile (entry 4), yielded 82–87%. Tetrahydrofuran with a high CED (270.8 MJ-eq/kg; entry 3) gave a slightly lower yield (79%). Based on these results, ethanol was chosen as the preferred solvent due to its slightly higher yield and favorable environmental profile.

**Table 1:**
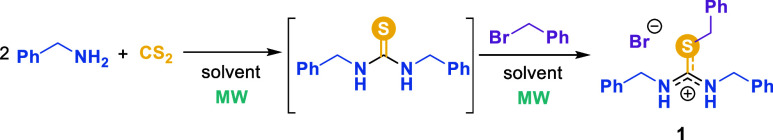
Optimization Process for the Formation of Isothiouronium Salts^a^

Entry	Solvent	Solvent production CED per kg solvent (MJ-eq)	Procedure	Yield (%)
1	EtOH	50.1	A	84
2	Toluene	80	A	87
3	THF	270.8	A	79
4	CH3CN	88.5	A	85
5	*t*-BuOH	-	A	73
6	MeOH	40.7	A	78
7	i-PrOH	65.6	A	82
8	EtOH	50.1	B	52
9	EtOH	50.1	C	97

a Reaction conditions: ***Procedure A***, step 1: MW (100 °C, 10 min); step 2: benzyl bromide addition, MW (100 °C, 15 min). ***Procedure B***, step 1: sealed tube in oil bath (100 °C, 10 min); step 2: benzyl bromide addition, sealed tube in oil bath (100 °C, 15 min). ***Procedure C***, step 1: MW (160 °C, 10 min); step 2: benzyl bromide addition, MW (100 °C, 15 min).

The effect of microwave irradiation was next investigated. A control reaction was carried out using conventional thermal heating in a sealed vessel, maintaining temperature and time conditions similar to those above (method B; entry 8). The yield decreased to 52%, highlighting the essential role of microwave irradiation in improving the efficiency.

To further improve the yield, we evaluated the effect of increasing the temperature at 160 °C in the first stage and maintaining the second stage at 100 °C, using ethanol as a solvent (procedure C). This modification led to a nearly quantitative yield of the corresponding isothiouronium salt (97% yield; entry 9).

Thus, the optimal conditions consisted of ethanol as the solvent, an initial heating stage at 160 °C for 10 min, followed by a second stage at 100 °C for 15 min after benzyl bromide addition.

With the optimal conditions in hand, we explored the reaction scope. To this end, we selected three primary amines—benzylamine, cyclohexylamine, and hexylamine—along with three alkylating agents: benzyl bromide, allyl bromide, and hexyl bromide. The corresponding reactions were conducted following Procedure C, and the results are presented in Scheme 2.

**Scheme 2:**
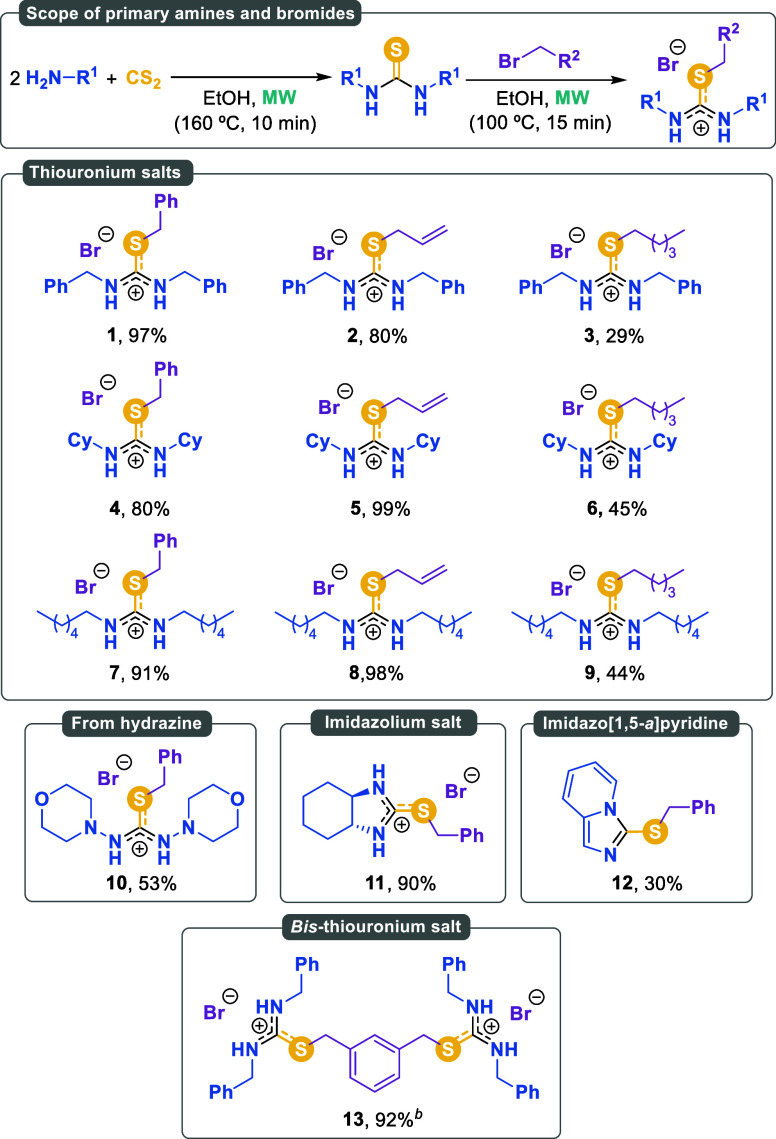
Scope of Amines and Alkyl Bromides^a^

a Reaction conditions: step 1: amine (2 mmol, 2 equiv), carbon disulfide (1 mmol, 1 equiv) in EtOH (5 mL), MW irradiation, 10 min, 160 °C; step 2: bromide (1.2 mmol, 1.2 equiv), MW irradiation, 15 min, 100 °C; isolated yields. ^b^Step 1: benzylamine (1 mmol, 2 equiv), carbon disulfide (0.5 mmol, 1 equiv) in EtOH (5 mL), MW irradiation, 10 min, 160 °C; step 2: 1,3-*bis*(bromomethyl)­benzene (0.25 mmol, 0.5 equiv), MW irradiation, 15 min, 100 °C.

The reaction of benzylamine with benzyl bromide proceeded following the previously described conditions, yielding product **1** in a nearly quantitative yield. A similar outcome was obtained with benzylamine and allyl bromide, which produced compound **2** in 80% yield. However, alkylation of the thiourea derived from benzylamine using hexyl bromide resulted in a lower yield, affording isothiouronium salt **3** in only a 29% yield. A comparable trend was observed when cyclohexylamine was reacted with all three alkylating agents. Both benzyl bromide and allyl bromide provided high yields of **4** and **5** (80% and 99%, respectively). The yield decreased when hexyl bromide was used as the alkylating agent, affording compound **6** in 45% yield. Finally, the reaction of hexylamine with benzyl bromide and allyl bromide gave yields approaching quantitative, affording compounds **7** and **8** in 91% and 98% yield, respectively. Similarly, hexyl bromide as the sulfur alkylating agent afforded compound **9** in a moderate 44% yield.

These promising results prompted us to explore more complex amines, yielding favorable outcomes. The hydrazine morpholin-4-amine afforded 2-benzyl-1,3-dimorpholinoisothiouronium bromide **10** in a moderate yet promising 53% yield. The presence of a second amino group in the molecule, regardless of its position on the carbon framework, was also well tolerated. For example, when cyclohexane-1,2-diamine was treated with carbon disulfide followed by benzyl bromide addition, perhydrobenzoimidazolium bromide **11** was afforded in an excellent 90% yield. A similar result was obtained using 2-picolylamine to give imidazo­[1,5-*a*]­pyridine **12** in 30% yield, which was isolated in its neutral form. These two examples illustrate the potential of this reaction for accessing different families of heterocycles. The synthesis of a double isothiouronium salt was confirmed by reacting 1,3-*bis*(bromomethyl)­benzene as the alkylating reagent, affording *bis*-isothiouronium salt **13** in 92% yield.

Building on the work by Botta and coworkers, who used propargyl bromide as the alkylating agent in a basic medium, we sought to explore the possibility of isolating the corresponding isothiouronium salts under neutral conditions. To this end, we subjected various amines to treatment with propargyl bromide under standard conditions and obtained crude mixtures containing the expected isothiouronium intermediates. However, upon purification by silica gel chromatography, cyclization occurred, leading to the exclusive formation of thiazolidinium salts. Silica gel-promoted cyclizations have been reported previously, particularly in Nazarov-type reactions of pentadienic systems. Hashmi and coworkers reported that silica gel, acting as a fixed-bed catalyst,^19^ enables the cyclization of vinyl allenyl ketones.^20^ Similarly, Dhoro and Tius showed that silica gel catalyzes amine-interrupted Nazarov cyclizations of allenyl vinyl ketones.^21^ This behavior observed during the purification process prompted us to investigate the scope and limitations of this transformation.^22,23^ The results are shown in Scheme 3.

**Scheme 3:**
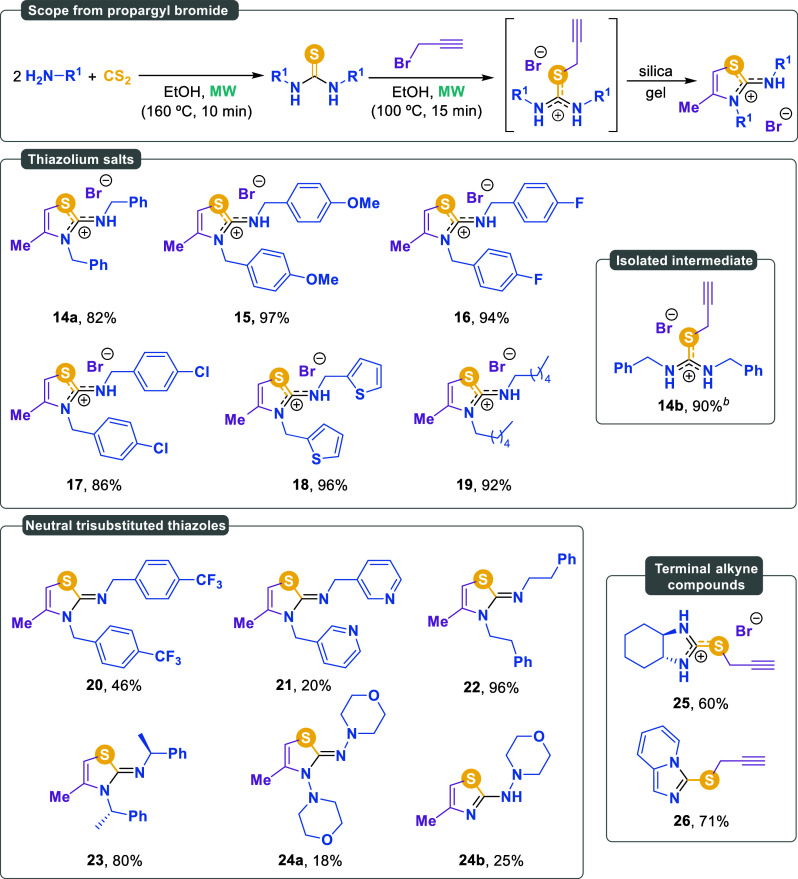
Scope of Thiazolidinium Salts using Propargyl Bromide as an Alkylating Reagent^a^

a Reaction conditions: step 1: amine (2 mmol, 2 equiv), carbon disulfide (1 mmol, 1 equiv) in EtOH (5 mL), MW irradiation, 10 min, 160 °C; step 2: propargyl bromide (1.2 mmol, 1.2 equiv), MW irradiation, 15 min, 100 °C; step 3: silica gel column chromatography; isolated yields. ^b^Isolated by crystallization and followed by filtration, avoiding the use of silica gel.

We selected various substituted benzylamines to study the influence of the electronic effects. In the case of benzylamine, we successfully isolated the corresponding isothiouronium salt **14b** through successive recrystallization. However, when the crude mixture was subjected to silica gel chromatography, thiazolidinium salt **14a** was inevitably obtained. The presence of electron-withdrawing or electron-donating substituents on the aromatic ring had a negligible impact. *p-*Methoxybenzylamine, *p-*fluorobenzylamine, and *p-*chlorobenzylamine provided excellent yields of the corresponding thiazolidinium salts **15**-**17** (97%, 94%, and 86%, respectively). Likewise, the presence of a thiophene ring in the amine was well tolerated. When 2-thiophenemethylamine was used, the corresponding thiazolidinium salt **18** was obtained in 96% yield. Finally, an aliphatic amine, such as hexylamine, also gave the corresponding thiazolidinium salt **19** in 92% yield.

Some of the cyclization products were isolated in their neutral form as trisubstituted thiazoles. 4-Trifluoromethylbenzylamine afforded thiazole **20** in 46% yield, while 3-picolylamine provided thiazole **21** in only 20%. In the latter case, the presence of a pyridine ring proved to be detrimental to the reaction outcome. In contrast, 2-phenylethylamine led to thiazole **22** in almost quantitative yield (96%). Likewise, the chiral amine (*S*)-1-phenylethan-1-amine afforded the corresponding chiral thiazole **23** in a particularly good yield of 80% yield. Remarkably, the hydrazine derivative *N*-aminomorpholine yielded thiazole **24a** (18%) and **24b** (25%), the latter resulting from the cleavage of one of the N–N bonds. Diamines, such as cyclohexane-1,2-diamine and 2-picolylamine, did not undergo cyclization. Instead, these substrates led to the formation of isothiouronium salt **25** (60%), while compound **26** (71%) was obtained as a neutral product featuring a propargyl group as a pendant substituent.

The use of other structurally related bromides as precursors of thiazoles was subsequently investigated (Scheme 4). Specifically, 1,2-dibromoethane led to thiazole **27** in 52% yield, confirming that the presence of a second bromine atom two bonds away can induce the cyclization process. A conjugated double bond, as in the case of 4-bromocrotonate, also acted as an electrophilic site for cyclization, yielding thiazolidinium bromide **28** (60%). Meanwhile, ethyl 2-bromopropanoate afforded thiazolidine-4-one **29** in a more modest 24% yield.

**Scheme 4:**
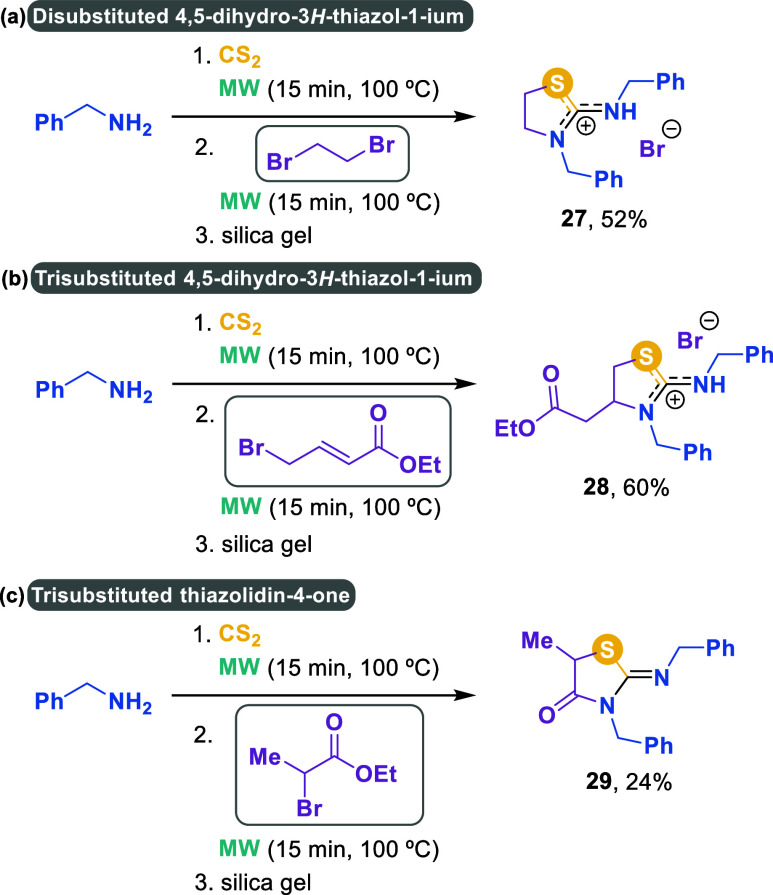
Structural Diversity using Different Dielectrophiles

Finally, it was observed that 2-bromoacetyl bromide, ethyl 2-bromoacetate, and *N*-(2-bromoacetyl)-2-amino-1,3-thiazole all led to the formation of the same *N*,*N’*-dibenzylthiazolidinone **33** (Scheme 5), confirming the preference of sulfur to attack the methylene group bearing the bromine atom. The case of 2-bromoacetyl bromide is particularly noteworthy, as it suggests that the nucleophilic attack by the nitrogen atom on the acyl group precedes the displacement of the bromine atom at C-2 by the sulfur atom.

**Scheme 5:**
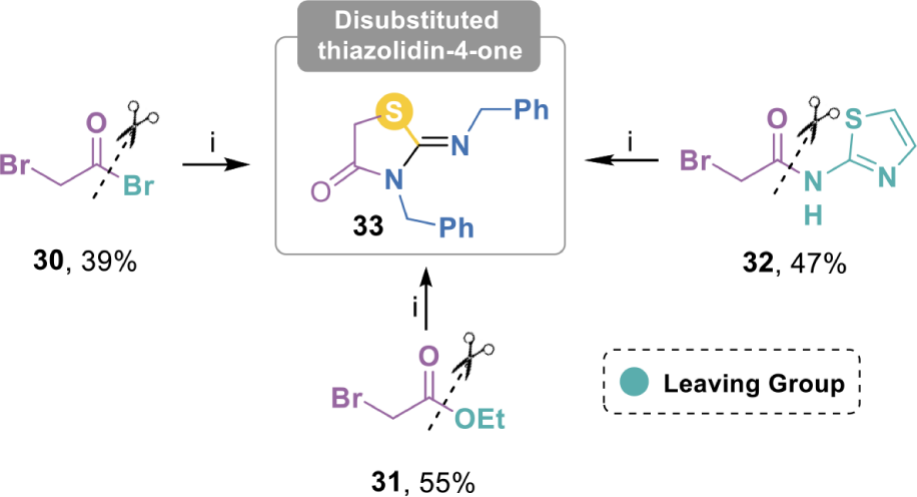
Access to Disubstituted Thiazolidine-4-one **33** from Different Bromide-Containing Electrophiles^a^

a (i) Reaction conditions: step 1: amine (2 mmol, 2 equiv), carbon disulfide (1 mmol, 1 equiv) in EtOH (5 mL), microwave irradiation, 10 min, 160 °C; step 2: dielectrophile (1.2 mmol, 1.2 equiv), microwave irradiation, 15 min, 100 °C; step 3: silica gel column chromatography; isolated yields.

### Computational Studies on the Cyclization of Isothiouronium Salts

Botta and coworkers have shown that in basic media, it is not possible to isolate the neutral form of the propargyl-containing thiazolidinium salts, as they easily evolve into their aminothiazole counterparts via cyclization.^17^ In fact, they proposed two possible mechanisms for this process, which differ in the sequence of the steps involved in the rearrangement—namely, C–N bond formation and [1,3] hydride shift. These mechanistic proposals have been adapted in Scheme 6 to account for the species observed herein under acidic conditions. In the so-called *mechanism A*, cyclization already takes place at the isothiouronium stage, yielding intermediate **II**, which then isomerizes to the final product **IV** via a [1,3] hydride shift. In contrast, *mechanism B* proposes that the propargyl moiety first isomerizes into allene **III**, followed by the cyclization step leading to the thiazolidinium ring in **IV**.

**Scheme 6:**
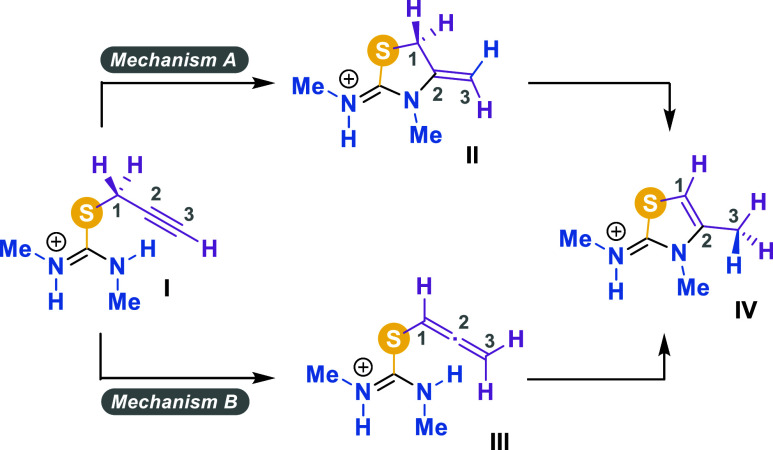
Suggested Mechanisms for the Isomerization of Isothiouronium Salts into Thiazolidinium Salts

In order to shed light on the mechanism of such isomerization, herein, we carried out a computational (DFT) analysis of the two mechanistic pathways depicted in Scheme 6, considering both the absence and presence of silica gel. All calculations were performed at the [SMD­(methanol)/M06-2*X*/6 + 31+G­(d,p)] level of theory (see the section Computational Details). To reduce the computational cost, the study started from a simplified model of compound **14b**, in which the amine-bound benzyl groups were replaced with lighter methyl groups. The resulting structure is termed species **I** in Scheme 6.

Thus, the computational results obtained so far are consistent with the experimental observations, indicating that the isomerization of **I** into **IV** is not kinetically viable in the absence of an appropriate catalyst (Figure 2). Both mechanisms depicted in Scheme 6 were subsequently examined in the presence of amorphous silica gel. Notably, silicic acids are known to catalyze a variety of organic transformations,^24–27^ acting both as Lewis acids and Lewis bases. In this study, and based on a number of literature precedents,^24,25,28^ along with the fact that the silica surface is known to become negatively charged at pH values above 6,^21^ we modeled this catalyst system by employing a deprotonated dimeric form of silicic acid (Si_2_O_7_H_5_^–^, denoted as **Si**).

**Figure 2:**
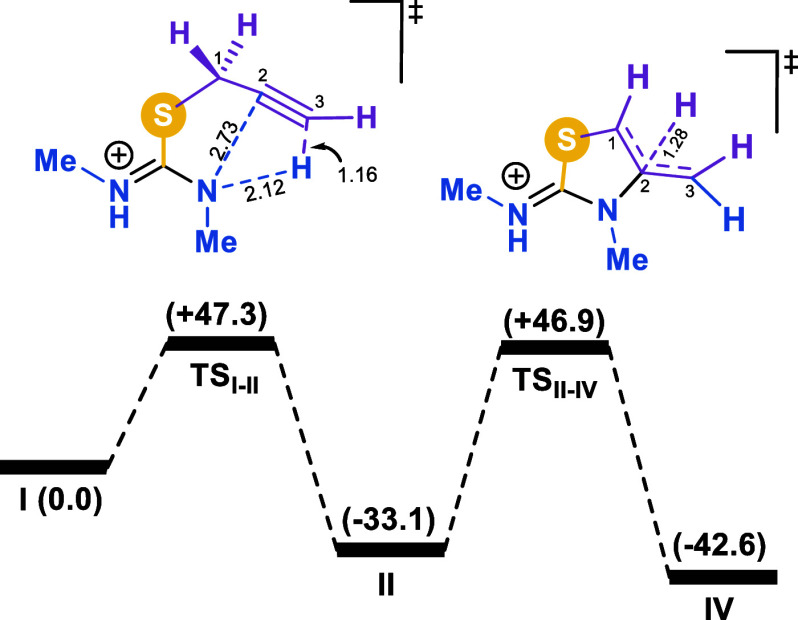
Computed free energy profile (kcal·mol^–1^) for the isomerization of **I** into **IV** via *mechanism A*.

According to the computations, the most stable adduct between **I** and silica gel (**Si**), labeled as **I·Si** in Figure 3, is weakly stabilized by 1.8 kcal·mol^–1^, primarily through a hydrogen-bond interaction between one of the amine protons and an anionic oxygen center in **Si** (d­(O···H) = 1.67 Å). From this adduct, mechanistic pathways analogous to those depicted in Scheme 6 were computationally explored. As previously mentioned, silica gel is known to catalyze a variety of organic transformations, and in this case, its dual ability to donate and accept protons enables it to catalyze the isomerization of **I** into **II**. This transformation proceeds through a transition state (**TS**_**I–II**_ **·Si**) in which **Si** behaves as a proton shuttle between the amine group and the C3 center. Simultaneously, this process involves the crucial C–N bond formation that generates the thiazole ring. Notably, comparison of **TS**_**I–II**_ **·Si** with the previous **TS**_**I–II**_ in the absence of silica gel reveals major structural differences, particularly in terms of the C–N distance. While **TS**_**I–II**_ corresponds to a very early transition state, characterized by a long C···N distance of 2.73 Å—a consequence of the hydrogen transfer from the amine group to the C3 position—the hydrogen-shuttling role of the silica avoids this constraint. As a result, the corresponding transition state **TS**_**I–II**_ **·Si** displays a significantly shorter C···N distance of 1.95 Å. In accordance with the Hammond–Leffler postulate,^29,30^ this structural shift corresponds to a notable decrease in the free energy barrier, which drops to only 18.3 kcal·mol^–1^. This step is also highly exergonic, with the resulting **II·Si** being 24.2 kcal·mol^–1^ more stable than **I·Si**. Analysis of **II·Si** reveals no significant hydrogen bonding interaction between the organic moiety and the catalyst, suggesting that the adduct is primarily stabilized by weak dispersion interactions (see Figure S69). Moving to the second step of *mechanism A*, the results again indicate that silica gel can facilitate proton transfer, effectively catalyzing the [1,3] hydride shift from C1 to C3 that generates the final **IV·Si**. The corresponding transition state **TS**_**II–IV**_ **·Si** shows both hydrogen atoms involved in the process positioned closer to the silicic catalyst, while the organic fragment retains an almost planar geometry (ϕ_N–C1–C2–C3_ = 175.4°). Energetically, **TS**_**II–IV**_ **·Si** lies only 11.6 kcal·mol^–1^ above **II·Si**, making this step even more accessible than the initial cyclization. These findings confirm that mechanism A becomes kinetically accessible in the presence of silica gel, thereby enabling the isomerization of **I** into **IV**. The results of the computational analysis of *mechanism B* in the presence of silica gel are shown in Figure 4. Initial calculations aimed at locating a concerted transition state for the alkyne–allene isomerization, leading to intermediate **III**. However, no such transition state could be identified. These findings are consistent with the literature, as alkyne–allene rearrangements are typically base-catalyzed,^31^ and our results indicate that silica gel can act as a Brønsted base in this context. Specifically, silica can deprotonate the C1 position of **I** via **TS**_**I‑A**_ **·Si** (Δ*G* ^‡^ = 13.8 kcal·mol^–1^) leading to the formation of intermediate **A·Si,** which lies 11.8 kcal·mol^–1^ above **I·Si** in free energy.

**Figure 3:**
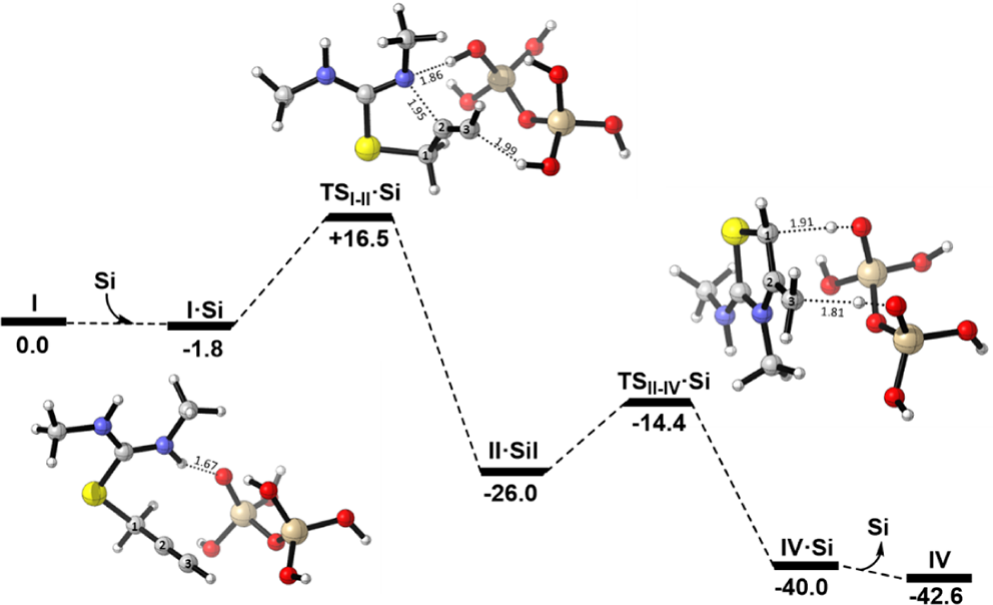
Computed free energy profile (kcal·mol^–1^) for the isomerization of **I** into **IV** in the presence of Si_2_O_7_H_5_^–^ (**Si**) via *mechanism A*. Color code: H, white; C, gray; N, blue; S, yellow; O, red; Si, brown.

**Figure 4:**
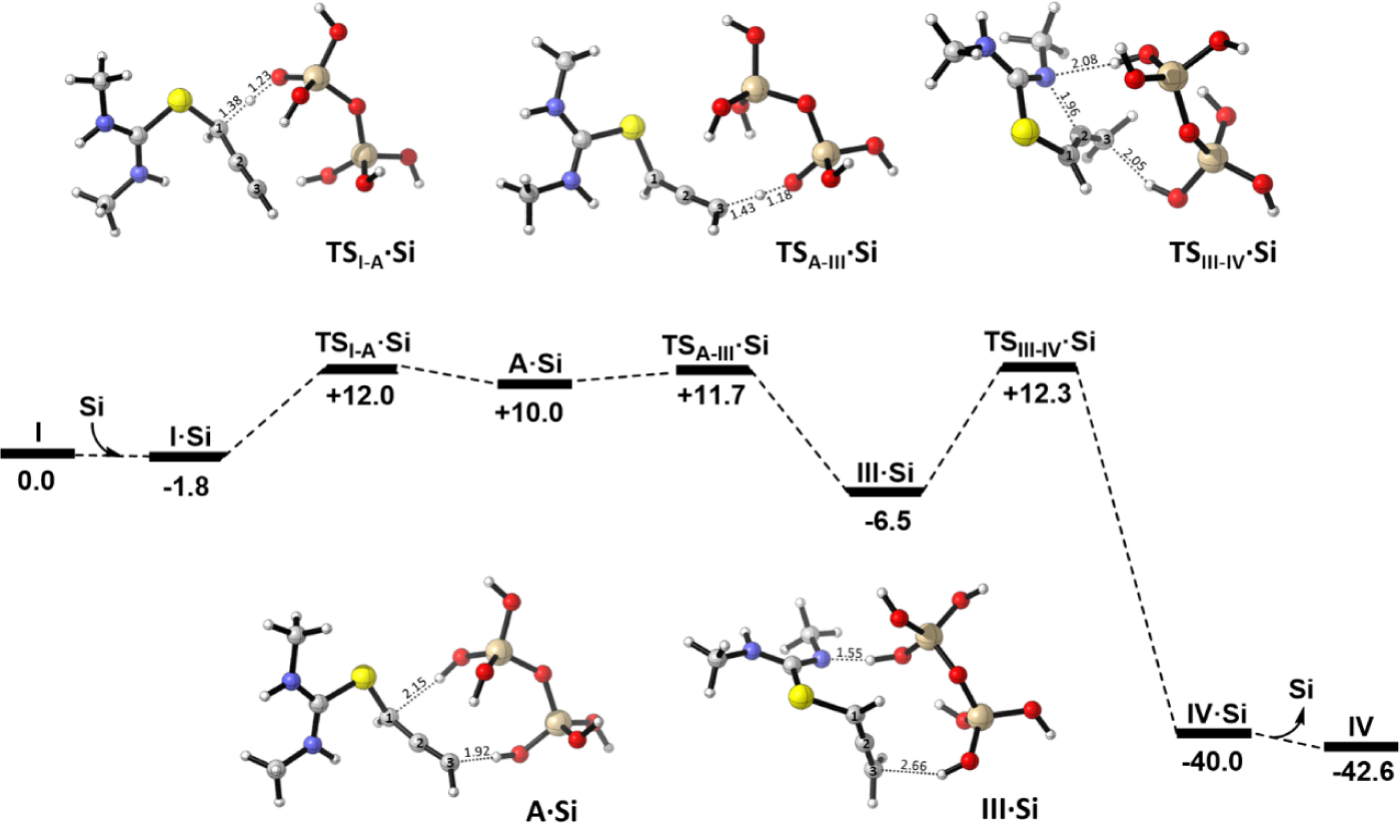
Computed free energy profile (kcal·mol^–1^) for the isomerization of **I** into **IV** in the presence of Si_2_O_7_H_5_^–^ (**Si**) via *mechanism B*. Color code: H, white; C, gray; N, blue; S, yellow; O, red; Si, brown.

Intermediate **A·Si** is stabilized by two hydrogen-bonding interactions with the silica surface (see Figure 4), which also promote the subsequent proton transfer needed to form allene intermediate **III·Si**. Despite proceeding through a stepwise pathway, the overall alkyne–allene isomerization is energetically favorable, with the second H-transfer step featuring a negligible barrier. Overall, the isomerization from **I·Si** to **III·Si** is exergonic by 4.7 kcal·mol^–1^. The resulting **III·Si** species can then undergo the cyclization step that results in the final **IV·Si**, this step again being rate-determining and taking place with an 18.8 kcal·mol^–1^ barrier via transition state **TS**_**IIIIV**_ **·Si**. This step involves not only the formation of the C–N bond (1.96 Å) but also a proton transfer from the silica to the C3 position, as reflected by a C3···*H—*O distance of 2.05 Å. Interestingly, **TS**_**III–IV**_ **·Si** presents structural similarities with **TS**_**I–II**_ **·Si** from *mechanism A*, although the latter also involves amine deprotonation, leading to a shorter N*H*···O distance (1.86 Å vs 2.08 Å in **TS**_**III–IV**_ **·Si**).

All in all, the computational studies herein demonstrate that in agreement with the experimental observations the uncatalyzed isomerization of **I** into **IV** is not kinetically feasible. However, silica gel can act as an effective catalyst for such transformation by facilitating the proton transfers involved in the process, thereby lowering the free energy barriers of each individual step. Interestingly, the results indicate that the C–N bond formation, which generates the thiazole ring, constitutes the most energy-demanding step of the entire isomerization, regardless of whether it takes place at the initial isothiouronium salt **I** via *mechanism A* (Δ*G* ^‡^= 18.3 kcal·mol^–1^) or at the allene intermediate **III** via *mechanism B* (Δ*G* ^‡^= 18.8 kcal·mol^–1^). Moreover, the small energy difference of only 0.5 kcal·mol^–1^ between the rate-determining steps of those mechanisms seems to indicate that both mechanisms may operate under the experimental conditions.

## Conclusions

In summary, we have developed a rapid, one-pot, two-step procedure to access isothiouronium salts using carbon disulfide as a simple and inexpensive sulfur source. The process proceeds through *in situ* generation of the corresponding thiourea, which reacts with various alkylating agents under microwave irradiation. The whole process is completed in less than 30 min, and the final isothiouronium salts are usually isolated in good to excellent yields. When propargyl bromide is used as the alkylating agent, cyclization to thiazolidinium salts is observed after purification via column chromatography on silica gel. This transformation occurs exclusively during purification, as no cyclization is observed under the reaction conditions themselves. Based on the interest of this silica gel-promoted cyclization, we conducted a computational (DFT) study to explore its underlying mechanism. In agreement with the experimental results, the uncatalyzed isomerization of isothiouronium salts into their thiazolidinium counterparts is computed to be not viable under the reaction conditions. On the contrary, the process becomes kinetically accessible when modeled in the presence of amorphous silica gel, which effectively reduces the free energy barriers of all the steps within the rearrangement by facilitating the associated H migrations. Notably, the formation of the C–N bond that generates the thiazole ring represents the rate-determining step of the whole rearrangement, and it can take place either at the initial isothiouronium salt or at an allene intermediate, thus leading to two alternative mechanisms. These feature very similar free energy barriers (18.3 and 18.8 kcal·mol^–1^, respectively), which suggests that they can both operate in the presence of amorphous silica gel.

## Experimental Section

### General One-Pot Sequential Approach (Two-Step) Procedure for the Preparation of Thiouronium Salts, Imidazolium Salts, and Imidazo­[1,5-*a*]­pyridines

Carbon disulfide (1 mmol) was carefully added dropwise to the amine (2 mmol, 2 equiv), resulting in the immediate formation of a solid intermediate. **Caution!** Rapid addition of carbon disulfide produced small popping sounds; the addition should be carried out slowly. After the addition of 5 mL of ethanol, the mixture was stirred under microwave irradiation (microwave irradiation methods are detailed in the footnotes of Schemes 2 and 3 and the Supporting Information). Upon formation of the *N,N’*-disubstituted thiourea, the corresponding bromide (1.2 mmol, 1.2 equiv) was added. The reaction mixture was then stirred further by using the optimal microwave method. The crude mixture was then directly purified by column chromatography on silica gel, yielding the desired compound.

### General One-Pot Sequential Approach (Two-Step) Procedure for the Preparation of Thiazolidinium Salts and Neutral Trisubstituted Thiazoles

Carbon disulfide (1 mmol) was carefully added dropwise to the amine (2 mmol, 2 equiv), leading to the immediate formation of a solid intermediate. **Caution!** Rapid addition of carbon disulfide produced small popping sounds; the addition should be carried out slowly. After the addition of 5 mL of ethanol, the mixture was stirred under microwave irradiation. Propargyl bromide (1.2 mmol, 1.2 equiv) was then added, and the reaction mixture was further stirred using the optimized microwave conditions to afford the open-chain terminal-alkyne salt. The crude reaction mixture was subsequently purified directly by column chromatography using wet silica gel (column dimensions: 2 cm diameter × 25 cm length), ensuring sufficient contact time with the stationary phase, which led to the formation of the desired cyclic compound.

### General Procedure for the Preparation of *bis*-Thiouronium Salt **13**

Carbon disulfide (0.5 mmol, 1 equiv) was carefully added dropwise to benzylamine (1 mmol, 2 equiv), leading to the formation of a solid intermediate. **Caution!** Rapid addition of carbon disulfide produced small popping sounds; the addition should be carried out slowly. After the addition of 5 mL of ethanol, the mixture was stirred under microwave irradiation. Upon formation of the thiourea, 1,3-*bis*(bromomethyl)­benzene (0.25 mmol, 0.5 equiv) was added. The reaction mixture was then stirred further under the optimized microwave conditions. The crude product was then directly purified by column chromatography on silica gel, affording compound **13**.
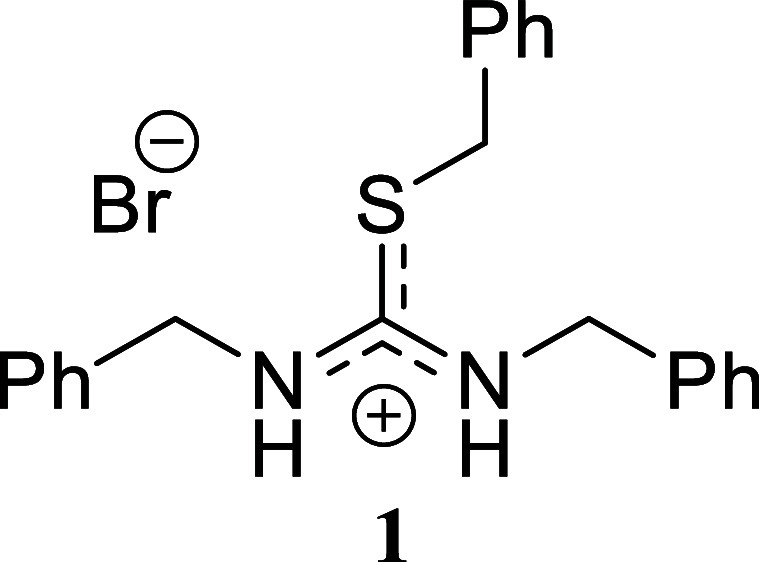


#### *S*-Benzyl-*N,N’*-dibenzylthiouronium Bromide **(1)**

White crystals (414.1 mg, 97%), mp 141.2–142.6 °C. Column chromatography eluent: petroleum ether/EtOAc gradient from 9:1 to pure EtOAc, followed by an EtOAc/MeOH gradient from 19:1 to 9:1. Rf 0.47 (1:9 v/v MeOH/DCM). ^1^H NMR (500 MHz, DMSO-*d*_6_) δ 9.98 (br s, 2H), 7.36–7.27 (m, 11H), 7.19 (br s, 2H), 7.11 (br s, 2H), 4.71 (br s, 2H), 4.65 (s, 2H), 4.61 (br s, 2H). ^13^C­{^1^H} NMR (125 MHz, DMSO-*d*_6_) δ 165.8, 136.3, 134.9, 134.5, 129.0, 128.8, 128.5, 128.1, 127.8, 127.6, 127.5, 127.0, 47.7, 46.7, 35.6. HRMS (ESI) *m*/*z*: [M]^+^ Calcd for C_22_H_23_N_2_S 347.1582, Found 347.1593; [^79^Br]^−^ Calcd for ^79^Br 78.9183, Found 78.9177; [^81^Br]^−^ Calcd for ^81^Br 80.9163, Found 80.9160. IR (ATR, cm^–1^): 3034, 2984, 1601, 1516, 1494, 1475, 1452, 1413, 1294, 1226, 1069, 1026, 952, 730, 717, 690, 608, 586, 564, 516, 500, 460.
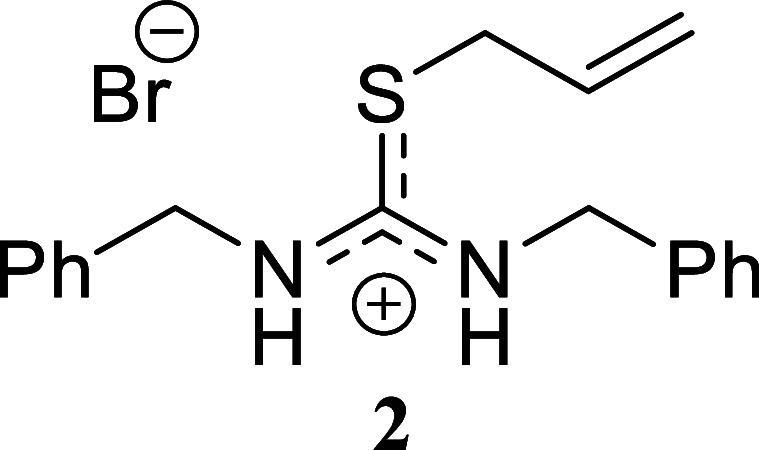


#### *S*-Allyl-*N,N’*-dibenzylthiouronium Bromide **(2)**

White crystals (301.9 mg, 80%), mp 87.5–89.2 °C. Column chromatography eluent: petroleum ether/EtOAc gradient from 9:1 to pure EtOAc, followed by an EtOAc/MeOH gradient from 19:1 to 9:1. Rf 0.32 (1:19 v/v MeOH/DCM). ^1^H NMR (400 MHz, DMSO-*d*_6_) δ 9.91 (br s, 2H), 7.47–7.27 (m, 8H), 7.27–7.13 (m, 2H), 5.85–5.68 (m, 1H), 5.25 (d, *J* = 17.0 Hz, 1H), 5.20–5.12 (m, 1H), 4.79–4.70 (m, 4H), 4.09–3.93 (m, 2H). ^13^C­{^1^H} NMR (100 MHz, DMSO-*d*_6_) δ 165.4 (from HMBC), 136.6, 135.2, 131.2, 128.6, 127.9, 127.7, 127.0, 120.1, 47.9, 46.8, 34.3. HRMS (ESI) *m*/*z*: [M]^+^ Calcd for C_18_H_21_N_2_S 297.1425, Found 297.1433; [^79^Br]^−^ Calcd for ^79^Br 78.9183, Found 78.9177; [^81^Br]^−^ Calcd for ^81^Br 80.9163, Found 80.9158. IR (ATR, cm^–1^): 3055, 3003, 2950, 2860, 1598, 1584, 1516, 1495, 1476, 1454, 1403, 1297, 1249, 1226, 1098, 1068, 1026, 994, 952, 939, 900, 870, 809, 730, 717, 695, 669, 594, 509, 499, 465, 449.
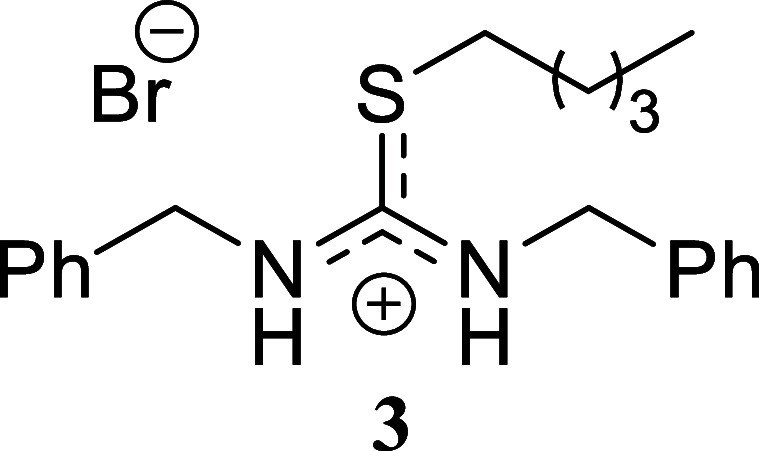


#### *N,N’*-Dibenzyl-*S*-pentylthiouronium Bromide **(3)**

White crystals (119.3 mg, 29%), mp 74.2–75.7 °C. Column chromatography eluent: petroleum ether/EtOAc gradient from 9:1 to pure EtOAc, followed by an EtOAc/MeOH gradient from 19:1 to 9:1. Rf 0.50 (1:9 v/v MeOH/DCM). ^1^H NMR (500 MHz, MeOD) δ 7.44–7.21 (m, 10H), 4.73 (s, 4H), 3.24 (t, *J* = 7.4 Hz, 2H), 1.60 (tt, *J* = 7.4, 7.4 Hz, 2H), 1.36–1.23 (m, 4H), 0.88 (t, *J* = 7.2 Hz, 3H). ^13^C­{^1^H} NMR (125 MHz, MeOD) δ 168.7, 137.0, 130.0, 129.2, 128.7, 49.0 (from HMBC), 33.5, 31.6, 29.7, 23.2, 14.3. HRMS (ESI) *m*/*z*: [M]^+^ Calcd for C_20_H_27_N_2_S 327.1895, Found 327.1903; [^79^Br]^−^ Calcd for ^79^Br 78.9183, Found 78.9172; [^81^Br]^−^ Calcd for ^81^Br 80.9163, Found 80.9150. IR (ATR, cm^–1^): 3347, 3064, 2957, 2929, 2871, 2291, 1596, 1583, 1515, 1495, 1478, 1454, 1417, 1347, 1067, 1028, 950, 922, 809, 740, 729, 716, 701, 694, 676, 602, 512, 463.
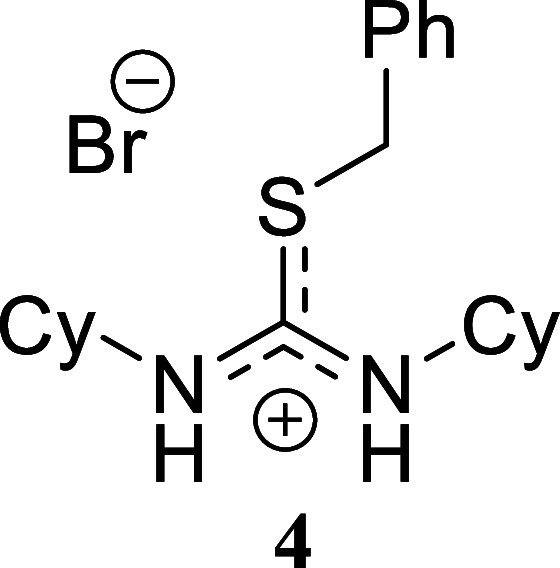


#### *S*-Benzyl-*N,N’*-diciclohexylthiouronium Bromide **(4)**

White crystals (328.5 mg, 80%), mp 90.5–92.0 °C. Column chromatography eluent: petroleum ether/EtOAc gradient from 9:1 to pure EtOAc, followed by an EtOAc/MeOH gradient from 19:1 to 9:1. Rf 0.42 (1:9 v/v MeOH/DCM). ^1^H NMR (500 MHz, MeOD) δ 7.37–7.33 (m, 2H), 7.33–7.28 (m, 2H), 7.26–7.22 (m, 1H), 4.12 (s, 2H), 3.49 (dddd, *J* = 10.5, 10.5, 3.6, 3.6 Hz, 2H), 1.72–1.64 (m, 8H), 1.64–1.57 (m, 2H), 1.34–1.10 (m, 10H). ^13^C­{^1^H} NMR (125 MHz, MeOD) δ 152.8, 139.5, 130.1, 129.7, 128.5, 56.6, 37.9, 34.9, 27.0, 26.4. HRMS (ESI) *m*/*z*: [M]^+^ Calcd for C_20_H_31_N_2_S 331.2208, Found 331.2221; [^79^Br]^−^ Calcd for ^79^Br 78.9183, Found 78.9171; [^81^Br]^−^ Calcd for ^81^Br 80.9163, Found 80.9154. IR (ATR, cm^–1^): 3326, 2925, 2852, 1604, 1524, 1497, 1452, 1236, 1152, 1103, 1065, 1031, 980, 779, 763, 712, 695, 591, 566, 518, 480, 453.
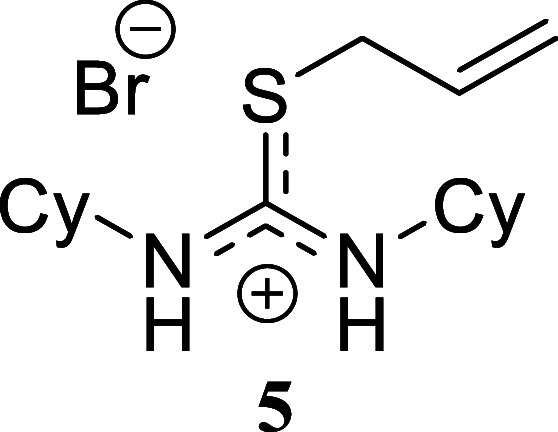


#### *S*-Allyl-*N,N’*-diciclohexylthiouronium Bromide **(5)**

Yellowish crystals (358.0 mg, 99%), mp 156.1–157.4 °C. Column chromatography eluent: petroleum ether/EtOAc gradient from 9:1 to pure EtOAc, followed by an EtOAc/MeOH gradient from 19:1 to 9:1. Rf 0.34 (1:9 v/v MeOH/DCM). ^1^H NMR (500 MHz, MeOD) δ 5.89 (ddt, *J* = 17.0, 10.0, 6.9 Hz, 1H), 5.34 (ddt, *J* = 17.0, 1.4, 1.4 Hz, 1H), 5.28 (ddt, *J* = 10.0, 1.0, 1.0 Hz, 1H), 3.90 (dt, *J* = 7.0, 1.1 Hz, 2H), 3.86 (br s, 1H), 3.73 (br s, 1H), 1.96–1.64 (m, 10H), 1.58–1.10 (m, 10H). ^13^C­{^1^H} NMR (125 MHz, MeOD) δ 164.8, 132.5, 121.1, 58.2, 55.0, 37.1, 33.9, 33.1, 26.3, 26.2, 26.0. HRMS (ESI) *m*/*z*: [M]^+^ Calcd for C_16_H_29_N_2_S 281.2051, Found 281.2060; [^79^Br]^−^ Calcd for ^79^Br 78.9183, Found 78.9170; [^81^Br]^−^ Calcd for ^81^Br 80.9163, Found 80.9154. IR (ATR, cm^–1^): 3323, 2926, 2851, 1661, 1624, 1565, 1448, 1362, 1340, 1310, 1243, 1102, 1088, 892, 640, 416.
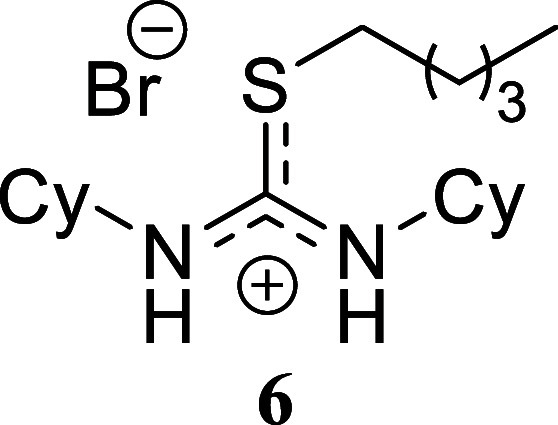


#### *N,N’*-Diciclohexyl-*S*-pentylthiouronium Bromide **(6)**

Yellowish oil (175.8 mg, 45%). Column chromatography eluent: petroleum ether/EtOAc gradient from 9:1 to pure EtOAc, followed by an EtOAc/MeOH gradient from 19:1 to 9:1. Rf 0.45 (1:9 v/v MeOH/DCM). ^1^H NMR (500 MHz, MeOD) δ 3.59 (dzddd, *J* = 10.5, 10.5, 3.8, 3.8 Hz, 2H), 2.89 (t, *J* = 7.3 Hz, 2H), 1.83–1.70 (m, 8H), 1.67–1.57 (m, 4H), 1.45–1.13 (m, 14H), 0.92 (t, *J* = 7.2 Hz, 3H). ^13^C­{^1^H} NMR (125 MHz, MeOD) δ 163.8 (from HMBC), 56.6, 35.0, 33.5, 32.1, 31.1, 27.0, 26.5, 23.5, 14.5. HRMS (ESI) *m*/*z*: [M]^+^ Calcd for C_18_H_35_N_2_S 311.2521, Found 311.2530; [^79^Br]^−^ Calcd for ^79^Br 78.9183, Found 78.9179; [^81^Br]^−^ Calcd for ^81^Br 80.9163, Found 80.9163. IR (ATR, cm^–1^): 2925, 2852, 1662, 1447, 1357, 1326, 1244, 1196, 1152, 1121, 1102, 1067, 889, 712.
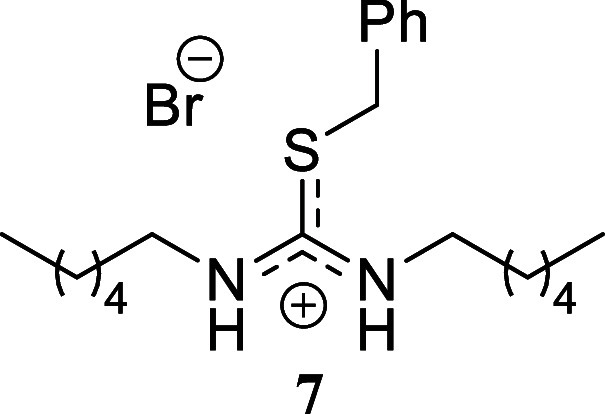


#### *S*-Benzyl-*N,N’*-dihexylthiouronium Bromide **(7)**

White crystals (378.1 mg, 91%), mp 57.8–59.2 °C. Column chromatography eluent: petroleum ether/EtOAc gradient from 9:1 to pure EtOAc, followed by an EtOAc/MeOH gradient from 19:1 to 9:1. Rf 0.42 (1:9 v/v MeOH/DCM). ^1^H NMR (500 MHz, MeOD) δ 7.42–7.39 (m, 2H), 7.38–7.34 (m, 2H), 7.33–7.29 (m, 1H), 4.39 (s, 2H), 3.34 (t, *J* = 7.4 Hz, 4H), 1.53 (tt, *J* = 7.4, 7.4 Hz, 4H), 1.34–1.25 (m, 12H), 0.91 (t, *J* = 7.0 Hz, 6H). ^13^C­{^1^H} NMR (125 MHz, MeOD) δ 163.5 (from HMBC), 136.9, 130.2, 130.1, 129.4, 46.6, 37.7, 32.7, 30.4, 27.6, 23.7, 14.5. HRMS (ESI) *m*/*z*: [M]^+^ Calcd for C_20_H_35_N_2_S 335.2521, Found 335.2531; [^79^Br]^−^ Calcd for ^79^Br 78.9183, Found 78.9179; [^81^Br]^−^ Calcd for ^81^Br 80.9163, Found 80.9154. IR (ATR, cm^–1^): 3328, 2956, 2928, 2870, 2857, 1612, 1573, 1478, 1461, 1230, 1290, 1249, 1220, 1076, 728, 699, 616, 587, 441.
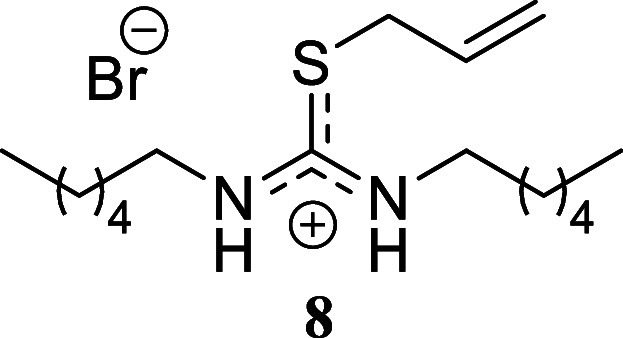


#### *S*-Allyl-*N,N’*-dihexylthiouronium Bromide **(8)**

Yellow crystals (359.5 mg, 98%), mp 56.2–58.0 °C. Column chromatography eluent: petroleum ether/EtOAc gradient from 9:1 to pure EtOAc, followed by an EtOAc/MeOH gradient from 19:1 to 9:1. Rf 0.42 (1:9 v/v MeOH/DCM). ^1^H NMR (500 MHz, MeOD) δ 5.91 (ddt, *J* = 17.0, 10.1, 6.9 Hz, 1H), 5.38 (ddt, *J* = 17.0, 1.2, 1.2 Hz, 1H), 5.29 (ddt, *J* = 10.1, 1.2, 1.2 Hz, 1H), 3.90 (ddd, *J* = 6.9, 1.2, 1.2 Hz, 2H), 3.52 (t, *J* = 7.2 Hz, 2H), 3.37 (t, *J* = 6.9 Hz, 2H), 1.69–1.61 (m, 4H), 1.41–1.27 (m, 12H), 0.95 (t, *J* = 6.7 Hz, 6H). ^13^C­{^1^H} NMR (125 MHz, MeOD) δ 167.2, 132.3, 121.1, 46.9, 45.5, 36.2, 32.6, 30.9, 29.1, 27.7, 27.4, 23.7, 14.5. HRMS (ESI) *m*/*z*: [M]^+^ Calcd for C_16_H_33_N_2_S 285.2364, Found 285.2375; [^79^Br]^−^ Calcd for ^79^Br 78.9183, Found 78.9179; [^81^Br]^−^ Calcd for ^81^Br 80.9163, Found 80.9162. IR (ATR, cm^–1^): 3327, 2956, 1929, 2870, 2857, 1612, 1574, 1478, 1461, 1300, 1290, 1249, 1220, 1076, 728, 616, 587, 441.
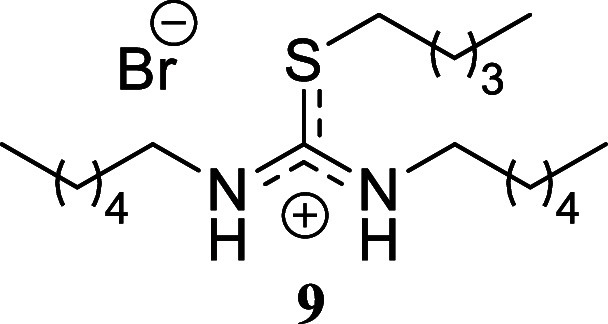


#### *N,N’*-Dihexyl-*S*-pentylthiouronium Bromide **(9)**

Colorless oil (175.2 mg, 44%). Column chromatography eluent: petroleum ether/EtOAc gradient from 9:1 to pure EtOAc, followed by an EtOAc/MeOH gradient from 19:1 to 9:1. Rf 0.58 (1:9 v/v MeOH/DCM). ^1^H NMR (500 MHz, MeOD) δ 3.27 (t, *J* = 7.1 Hz, 4H), 2.91 (t, *J* = 7.2 Hz, 2H), 1.63 (tt, *J* = 7.2, 7.2 Hz, 2H), 1.57–1.50 (m, 4H), 1.44–1.30 (m, 16H), 0.92 (t, *J* = 7.3 Hz, 3H), 0.91 (t, *J* = 7.0 Hz, 6H). ^13^C­{^1^H} NMR (125 MHz, MeOD) δ 157.0 (from HMBC), 47.5, 33.0, 32.8, 32.1, 31.5, 31.0, 28.1, 23.9, 23.5, 14.6, 14.5. HRMS (ESI) *m*/*z*: [M]^+^ Calcd for C_18_H_39_N_2_S 315.2834, Found 315.2841; [^79^Br]^−^ Calcd for ^79^Br 78.9183, Found 78.9171; [^81^Br]^−^ Calcd for ^81^Br 80.9163, Found 80.9175. IR (ATR, cm^–1^) 2956, 2927, 2857, 1655, 1565, 1459, 1340, 1259, 1196, 1103, 724, 594.
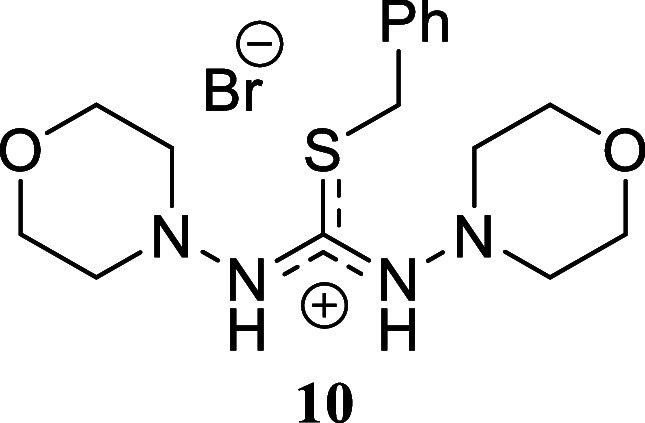


#### *S*-Benzyl-*N,N’*-dimorpholinothiouronium Bromide **(10)**

Yellow oil (220.7 mg, 53%). Column chromatography eluent: petroleum ether/EtOAc gradient from 9:1 to pure EtOAc, followed by an EtOAc/MeOH gradient from 19:1 to 9:1. Rf 0.70 (1:9 v/v MeOH/DCM). ^1^H NMR (500 MHz, MeOD) δ 7.39–7.20 (m, 5H), 4.18 (s, 2H), 3.76–3.70 (m, 4H), 3.65–3.60 (m, 4H), 3.27–3.21 (m, 4H), 2.70–2.63 (m, 4H). ^13^C­{^1^H} NMR (125 MHz, MeOD) δ 163.4, 140.1, 130.1, 129.7, 128.5, 67.7, 67.6, 57.0, 51.2, 38.1. HRMS (ESI) *m*/*z*: [M]^+^ Calcd for C_16_H_25_N_4_O_2_S 337.1698, Found 337.1703; [^79^Br]^−^ Calcd for ^79^Br 78.9183, Found 78.9177; [^81^Br]^−^ Calcd for ^81^Br 80.9163, Found 80.9155. IR (ATR, cm^–1^) 2962, 2839, 1584, 1547, 1454, 1262, 1212, 1196, 1105, 1070, 1033, 992, 771, 727, 702, 651, 619.
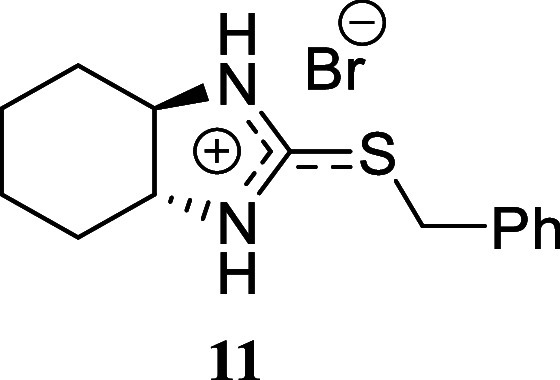


#### *trans*-2-(Benzylthio)-3a,4,5,6,7,7a-hexahydro-1*H*-benzo­[*d*]­imidazole-1-ium Bromide **(11)**

Yellowish oil (294.6 mg, 90%). Column chromatography eluent: petroleum ether/EtOAc gradient from 9:1 to pure EtOAc, followed by an EtOAc/MeOH gradient from 19:1 to 9:1. Rf 0.47 (1:9 v/v MeOH/DCM). ^1^H NMR (500 MHz, MeOD) δ 7.38–7.34 (m, 2H), 7.33–7.28 (m, 2H), 7.28–7.23 (m, 1H), 4.28 (d, *J* = 12.9 Hz, 1H), 4.21 (d, *J* = 12.9 Hz, 1H), 3.03–2.94 (m, 2H), 2.20–2.14 (m, 2H), 1.85–1.76 (m, 2H), 1.50–1.39 (m, 2H), 1.38–1.31 (m, 2H). ^13^C­{^1^H} NMR (125 MHz, MeOD) δ 169.2, 138.2, 130.1, 129.8, 128.7, 71.0, 35.9, 31.7, 26.0. HRMS (ESI) *m*/*z*: [M]^+^ Calcd for C_14_H_19_N_2_S 247.1269, Found 247.1275; [^79^Br]^−^ Calcd for ^79^Br 78.9183, Found 78.9175; [^81^Br]^−^ Calcd for ^81^Br 80.9163, Found 80.9160. IR (ATR, cm^–1^): 3223, 2933, 2859, 1703, 1606, 1527, 1453, 1373, 1348, 1335, 1305, 1260, 1229, 1201, 1140, 1104, 1057, 1037, 767, 697, 566, 539, 518, 503, 474.
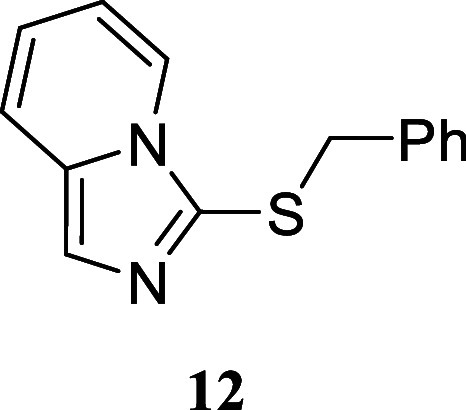


#### 3-(Benzylthio)­imidazo­[1,5-*a*]­pyridine **(12)**

Brown oil (71.9 mg, 30%). Column chromatography eluent: petroleum ether/EtOAc gradient from 9:1 to pure EtOAc. Rf 0.78 (1:9 v/v MeOH/DCM). ^1^H NMR (500 MHz, MeOD) δ 7.90 (dddd, *J* = 7.3, 1.1, 1.0, 1.0 Hz, 1H), 7.50 (d, *J* = 1.0 Hz, 1H), 7.49 (ddd, *J* = 9.1, 1.2, 1.1 Hz, 1H), 7.11–7.04 (m, 3H), 6.94–6.90 (m, 2H), 6.80 (ddd, *J* = 9.1, 6.5, 1.0 Hz, 1H), 6.54 (ddd, *J* = 7.3, 6.5, 1.2 Hz, 1H), 4.05 (s, 2H). ^13^C­{^1^H} NMR (125 MHz, MeOD) δ 139.2, 134.5, 129.9, 129.6, 128.6, 123.6, 121.9, 121.8, 119.3, 114.7, 41.8. HRMS (ESI) *m*/*z*: [M + H]^+^ Calcd for C_14_H_13_N_2_S 241.0799, Found 241.0813. IR (ATR, cm^–1^): 3100, 3075, 3050, 3025, 2925, 1638, 1500, 1450, 1400, 1350, 1250, 1013, 925, 800, 763, 738, 700, 663, 563, 475, 425.
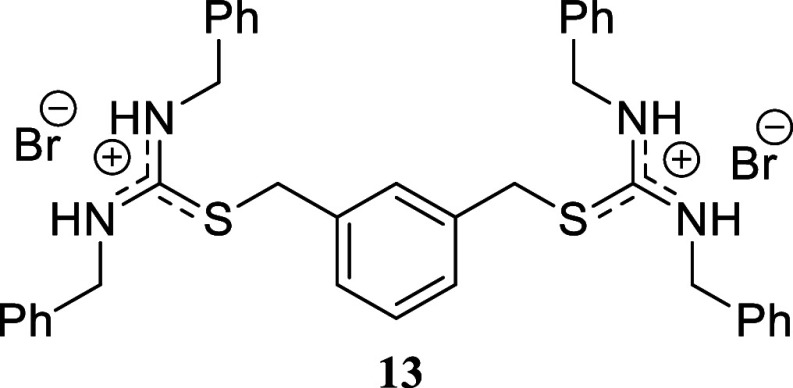


#### *S,S’*-(1,3-Phenylene*bis*(methylene))*bis*(*N,N’*-dibenzylthiouronium) Dibromide **(13)**

White crystals (178.6 mg, 92%), mp 100.7–102.1 °C. Column chromatography eluent: petroleum ether/EtOAc gradient from 9:1 to pure EtOAc, followed by an EtOAc/MeOH gradient from 19:1 to 9:1. Rf 0.52 (1:9 v/v MeOH/DCM). ^1^H NMR (500 MHz, MeOD) δ 7.34–7.24 (m, 16H), 7.10 (br s, 8H), 4.64 (s, 8H), 4.47 (s, 4H). ^13^C­{^1^H} NMR (125 MHz, MeOD) δ 166.9, 136.9, 131.1, 131.0, 130.4, 130.0, 129.2, 128.7, 49.3 (from HMBC), 37.4. HRMS (ESI) *m*/*z*: [M + H]^+^ Calcd for C_38_H_39_N_4_S_2_ 615.2616, Found 615.2637; [M + H – Bn]^+^ Calcd for C_31_H_33_N_4_S_2_ 525.2147, Found 525.2164; [M + 2H]^2+^ Calcd for [C_38_H_40_N_4_S_2_]^2+^ 308.1347, Found 308.1361; [^79^Br]^−^ Calcd for ^79^Br 78.9183, Found 78.9170; [^81^Br]^−^ Calcd for ^81^Br 80.9163, Found 80.9150. IR (ATR, cm^–1^): 2990, 1602, 1520, 1495, 1453, 1349, 1222, 1068, 1028, 956, 695, 456.
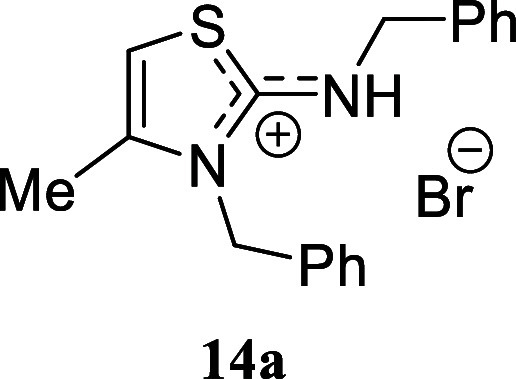


#### 3-Benzyl-2-(benzylamino)-4-methyl-3*H*-thiazol-1-ium Bromide **(14a)**

White crystals (306.5 mg, 82%), mp 202.1–203.5 °C. Column chromatography eluent: petroleum ether/EtOAc gradient from 9:1 to pure EtOAc, followed by an EtOAc/MeOH gradient from 19:1 to 9:1. Rf 0.50 (1:9 v/v MeOH/DCM). ^1^H NMR (500 MHz, MeOD) δ 7.46–7.41 (m, 2H), 7.41–7.38 (m, 1H), 7.36–7.32 (m, 3H), 7.30–7.26 (m, 2H), 7.13–7.08 (m, 2H), 6.78 (q, *J* = 1.3 Hz, 1H), 5.43 (s, 2H), 4.62 (s, 2H), 2.26 (d, *J* = 1.3 Hz, 3H). ^13^C­{^1^H} NMR (125 MHz, MeOD) δ 171.1, 141.0, 135.8, 134.4, 130.6, 130.2, 129.8, 129.7, 129.0, 127.0, 103.7, 51.9, 50.2, 14.2. HRMS (ESI) *m*/*z*: [M]^+^ Calcd for C_18_H_19_N_2_S 295.1269, Found 295.1283; [^79^Br]^−^ Calcd for ^79^Br 78.9183, Found 78.9173; [^81^Br]^−^ Calcd for ^81^Br 80.9163, Found 80.9155. IR (ATR, cm^–1^): 3061, 2255, 1588, 1577, 1496, 1480, 1454, 1380, 1357, 1323, 1243, 1197, 1162, 965, 840, 785, 756, 742, 708, 699, 666, 617, 456, 418.
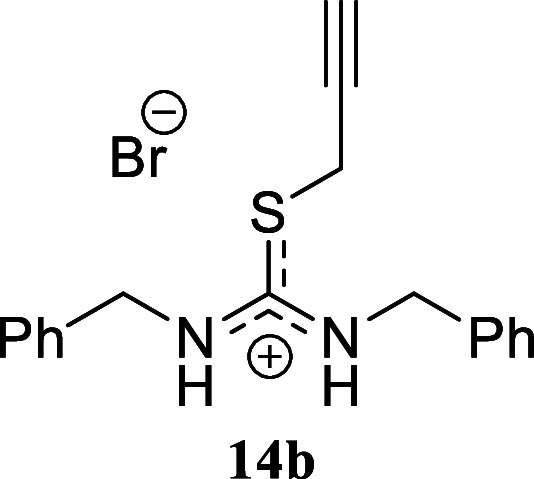


#### *N,N’*-Dibenzyl-*S*-(prop-2-yn-1-Yl)­thiouronium Bromide **(14b)**

Pinkish-brown crystals (337.3 mg, 90%), mp 104.2–105.3 °C. The solid was collected by filtration and washed with cold EtOH. Rf 0.58 (1:9 v/v MeOH/DCM). ^1^H NMR (500 MHz, MeOD) δ 7.49–7.29 (m, 8H), 7.27–7.18 (m, 2H), 4.76 (s, 4H), 4.16 (d, *J* = 2.6 Hz, 2H), 2.98 (t, *J* = 2.6 Hz, 1H). ^13^C­{^1^H} NMR (125 MHz, MeOD) δ 167.8, 137.1, 135.5, 130.2, 130.1, 129.7, 129.3, 129.2, 128.5, 77.9, 76.1, 49.5 (from HMBC), 22.2. HRMS (ESI) *m*/*z*: [M]^+^ Calcd for C_18_H_19_N_2_S 295.1269, Found 295.1284; [^79^Br]^−^ Calcd for ^79^Br 78.9183, Found 78.9176; [^81^Br]^−^ Calcd for ^81^Br 80.9163, Found 80.9154. IR (ATR, cm^–1^): 3283, 3110, 3069, 2961, 2868, 1616, 1535, 1496, 1484, 1455, 1433, 1418, 1327, 1352, 1301, 1227, 1200, 1168, 1065, 1023, 950, 742, 716, 692, 648, 611, 457.
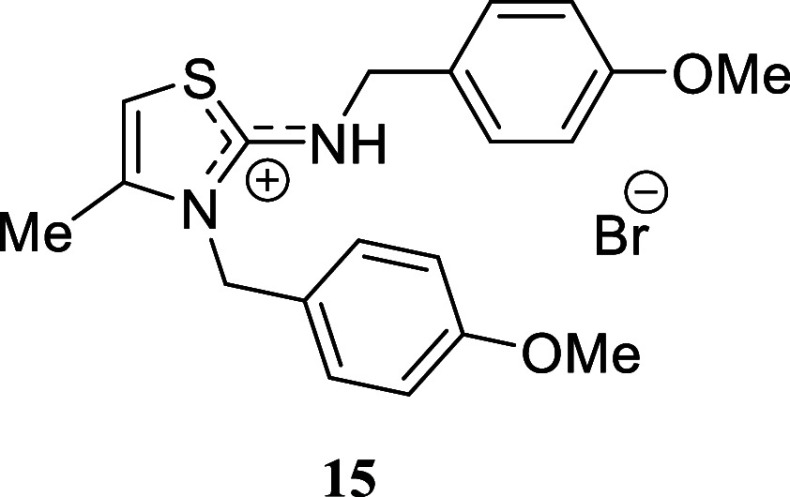


#### 3-(4-Methoxybenzyl)-2-((4-methoxybenzyl)­amino)-4-methyl-3*H*-thiazol-1-ium Bromide **(15)**

White crystals (422.4 mg, 97%), mp 190.7–191.0 °C. Column chromatography eluent: petroleum ether/EtOAc gradient from 9:1 to pure EtOAc, followed by an EtOAc/MeOH gradient from 19:1 to 9:1. Rf 0.50 (1:9 v/v MeOH/DCM). ^1^H NMR (400 MHz, MeOD) δ 7.23 (d, *J* = 8.3 Hz, 2H), 7.05 (d, *J* = 8.3 Hz, 2H), 6.96 (d, *J* = 8.8 Hz, 2H), 6.90 (d, *J* = 8.7 Hz, 2H), 6.74 (br s, 1H), 5.35 (s, 2H), 4.54 (s, 2H), 3.80 (s, 3H), 3.78 (s, 3H), 2.26 (d, *J* = 1.1 Hz, 3H). ^13^C­{^1^H} NMR (100 MHz, MeOD) δ 170.6, 161.45, 161.41, 140.9, 130.6, 128.63, 128.61, 127.6, 126.2, 115.9, 115.4, 103.6, 56.03, 55.95, 51.4, 49.8, 14.3. HRMS (ESI) *m*/*z*: [M]^+^ Calcd for C_20_H_23_N_2_O_2_S 355.1480, Found 355.1492; [^79^Br]^−^ Calcd for ^79^Br 78.9183, Found 78.9182; [^81^Br]^−^ Calcd for ^81^Br 80.9163, Found 80.9155. IR (ATR, cm^–1^): 3070, 2783, 1610, 1594, 1511, 1357, 1295, 1247, 1181, 1174, 1124, 1031, 866, 809, 762, 748, 684, 516.
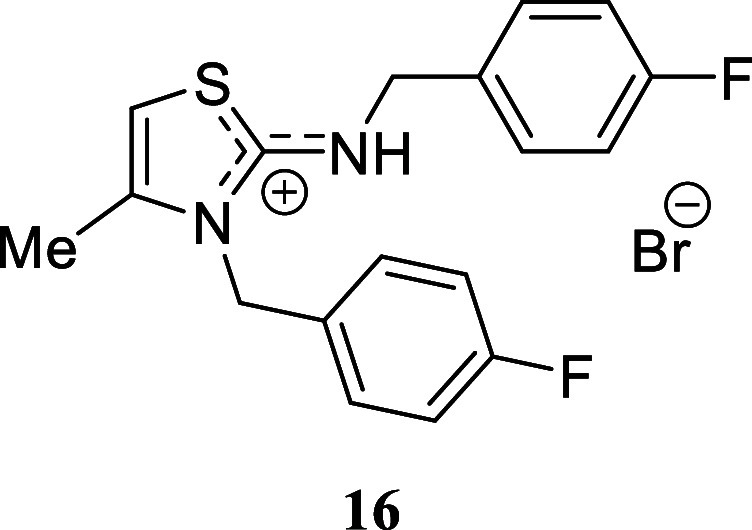


#### 3-(4-Fluorobenzyl)-2-((4-fluorobenzyl)­amino)-4-methyl-3*H*-thiazol-1-ium Bromide **(16)**

White crystals (386.7 mg, 94%), mp 161.9–163.0 °C. Column chromatography eluent: petroleum ether/EtOAc gradient from 9:1 to pure EtOAc, followed by an EtOAc/MeOH gradient from 19:1 to 9:1. Rf 0.68 (1:9 v/v MeOH/DCM). ^1^H NMR (400 MHz, MeOD) δ 7.40–7.31 (m, 2H), 7.19–7.14 (m, 4H), 7.12–7.06 (m, 2H), 6.78 (br s, 1H), 5.46–5.37 (m, 2H), 4.60 (s, 2H), 2.26 (d, *J* = 1.1 Hz, 3H). ^13^C­{^1^H} NMR (125 MHz, MeOD) δ 170.9, 164.3 (d, ^1^ *J*_C–F_ = 245.8 Hz), 164.2 (d,^1^ *J*_C–F_ = 245.7 Hz), 140.9, 131.9 (d, ^4^ *J*_C–F_ = 2.9 Hz), 131.2 (d, ^3^ *J*_C–F_ = 8.4 Hz), 130.5 (d, ^4^ *J*_C–F_ = 3.4 Hz), 129.2 (d, ^3^ *J*_C–F_ = 8.4 Hz), 117.4 (d, ^2^ *J*_C–F_ = 22.2 Hz), 116.9 (d, ^2^ *J*_C–F_ = 22.1 Hz), 103.7, 51.3, 49.6 (from HMBC), 14.2. ^19^F­{^1^H} NMR (376 MHz, CDCl_3_) δ −115.56, −115.59. HRMS (ESI) *m*/*z*: [M]^+^ Calcd for C_18_H_17_N_2_SF_2_ 331.1081, Found 331.1096; [^79^Br]^−^ Calcd for ^79^Br 78.9183, Found 78.9175; [^81^Br]^−^ Calcd for ^81^Br 80.9163, Found 80.9153. IR (ATR, cm^–1^): 3047, 2913, 2796, 2161, 1608, 1582, 1508, 1474, 1417, 1381, 1349, 1222, 1158, 1012, 835, 818, 510, 492, 475.
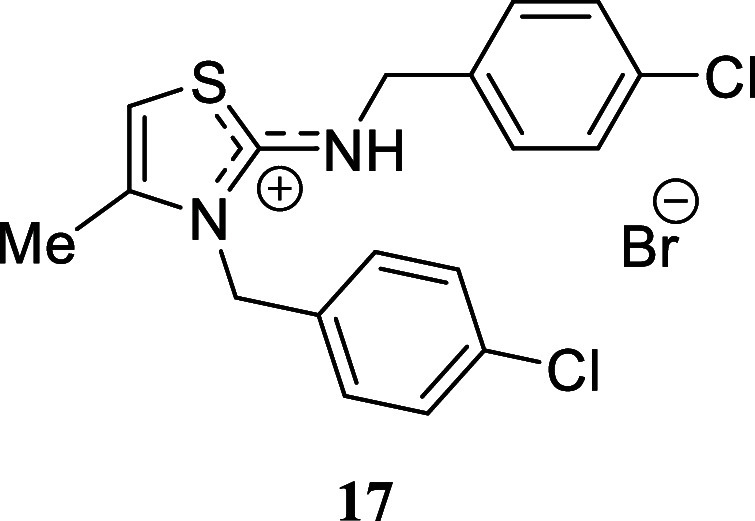


#### 3-(4-Chlorobenzyl)-2-((4-chlorobenzyl)­amino)-4-methyl-3*H*-thiazol-1-ium Bromide **(17)**

Yellow crystal (383.1 mg, 86%), mp 200.1–202.0 °C. Column chromatography eluent: petroleum ether/EtOAc gradient from 9:1 to pure EtOAc, followed by an EtOAc/MeOH gradient from 19:1 to 9:1. Rf 0.61 (1:9 v/v MeOH/DCM). ^1^H NMR (500 MHz, MeOD) δ 7.47–7.42 (m, 2H), 7.39–7.35 (m, 2H), 7.29–7.24 (m, 2H), 7.12–7.07 (m, 2H), 6.79 (q, *J* = 1.3 Hz, 1H), 5.38 (s, 2H), 4.61 (s, 2H), 2.27 (d, *J* = 1.3 Hz, 3H). ^13^C­{^1^H} NMR (125 MHz, MeOD) δ 171.0, 141.0, 135.6, 135.5, 134.6, 133.3, 130.8, 130.6, 130.2, 128.9, 104.0, 51.2, 49.8, 14.2. HRMS (ESI) *m*/*z*: [M]^+^ Calcd for C_18_H_17_N_2_SCl_2_ 363.0489, Found 363.0498; [^79^Br]^−^ Calcd for ^79^Br 78.9183, Found 78.9175; [^81^Br]^−^ Calcd for ^81^Br 80.9163, Found 80.9153. IR (ATR, cm^–1^): 3061, 2968, 2911, 2764, 1609, 1592, 1576, 1490, 1408, 1382, 1336, 1117, 1094, 1012, 870, 795, 770, 655, 623, 485, 445, 423.
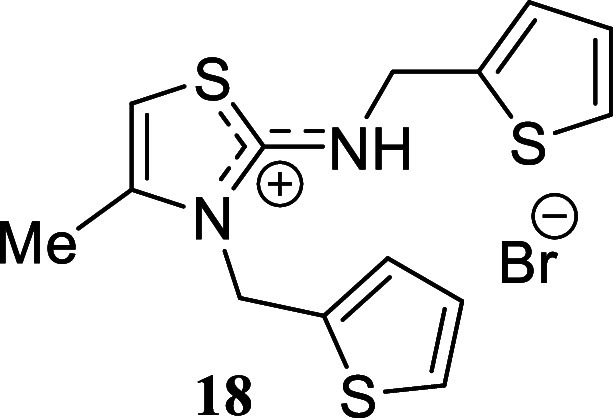


#### 4-Methyl-3-(thiophen-2-ylmethyl)-2-((thiophen-2-Ylmethyl)­amino)-3*H*-thiazol-1-ium Bromide **(18)**

Amorphous solid (371.9 mg, 96%). Column chromatography eluent: petroleum ether/EtOAc gradient from 9:1 to pure EtOAc, followed by an EtOAc/MeOH gradient from 19:1 to 9:1. Rf 0.71 (1:9 v/v MeOH/DCM). ^1^H NMR (500 MHz, MeOD) δ 7.47 (dd, *J* = 5.1, 1.2 Hz, 1H), 7.44 (dd, *J* = 5.1, 1.2 Hz, 1H), 7.19 (ddt, *J* = 3.5, 1.2, 1.2 Hz, 1H), 7.10 (ddt, *J* = 3.5, 1.2, 1.2 Hz, 1H), 7.05 (dd, *J* = 5.1, 3.5 Hz, 1H), 7.02 (dd, *J* = 5.1, 3.5 Hz, 1H), 6.74 (q, *J* = 1.3 Hz, 1H), 5.56 (d, *J* = 1.2 Hz, 2H), 4.89 (d, *J* = 1.2 Hz, 2H), 2.38 (d, *J* = 1.3 Hz, 3H). ^13^C­{^1^H} NMR (125 MHz, MeOD) δ 170.0, 140.3, 138.4, 136.7, 129.4, 128.5, 128.2, 127.8, 127.7, 103.5, 47.3, 45.9, 14.3. HRMS (ESI) *m*/*z*: [M]^+^ Calcd for C_14_H_15_N_2_S_3_ 307.0397, Found 307.0403; [^79^Br]^−^ Calcd for ^79^Br 78.9183, Found 78.9180; [^81^Br]^−^ Calcd for ^81^Br 80.9163, Found 80.9160. IR (ATR, cm^–1^): 2920, 1610, 1579, 1435, 1365, 1321, 1221, 1156, 851, 702.
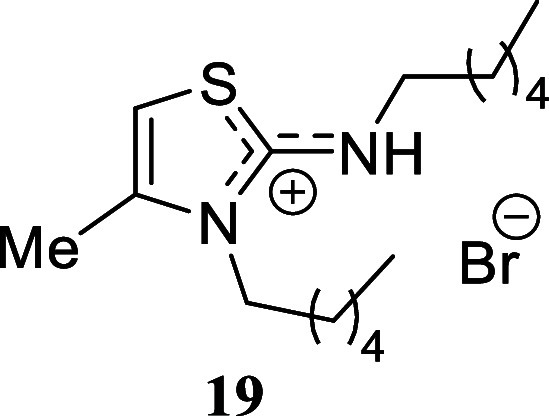


#### 3-Hexyl-2-(hexylamino)-4-methyl-3*H*-thiazol-1-ium Bromide **(19)**

Yellowish-brown crystals (333.8 mg, 92%), mp 50.2–51.4 °C. Column chromatography eluent: petroleum ether/EtOAc gradient from 9:1 to pure EtOAc, followed by an EtOAc/MeOH gradient from 19:1 to 9:1. Rf 0.45 (1:9 v/v MeOH/DCM). ^1^H NMR (400 MHz, CDCl_3_) δ 6.25 (br s, 1H), 4.30 (t, *J* = 7.4 Hz, 2H), 3.34 (t, *J* = 7.3 Hz, 2H), 2.25 (d, *J* = 1.0 Hz, 3H), 1.79 (tt, *J* = 7.4, 7.4 Hz, 2H), 1.75–1.66 (m, 2H), 1.44 (tt, *J* = 7.3, 7.3 Hz, 2H), 1.38–1.32 (m, 2H), 1.31–1.24 (m, 8H), 0.850 (t, *J* = 6.9 Hz, 3H), 0.846 (t, *J* = 7.1 Hz, 3H). ^13^C­{^1^H} NMR (100 MHz, CDCl_3_) δ 166.4, 138.1, 99.3, 49.3, 46.9, 31.4, 31.3, 28.0, 27.9, 26.5, 25.9, 22.43, 22.36, 14.5, 13.9. HRMS (ESI) *m*/*z*: [M]^+^ Calcd for C_16_H_31_N_2_S 283.2208, Found 283.2212; [^79^Br]^−^ Calcd for ^79^Br 78.9183, Found 78.9181; [^81^Br]^−^ Calcd for ^81^Br 80.9163, Found 80.9159. IR (ATR, cm^–1^): 2956, 2926, 2857, 1610, 1589, 1460, 1429, 1355, 1131, 841, 757, 726, 669.
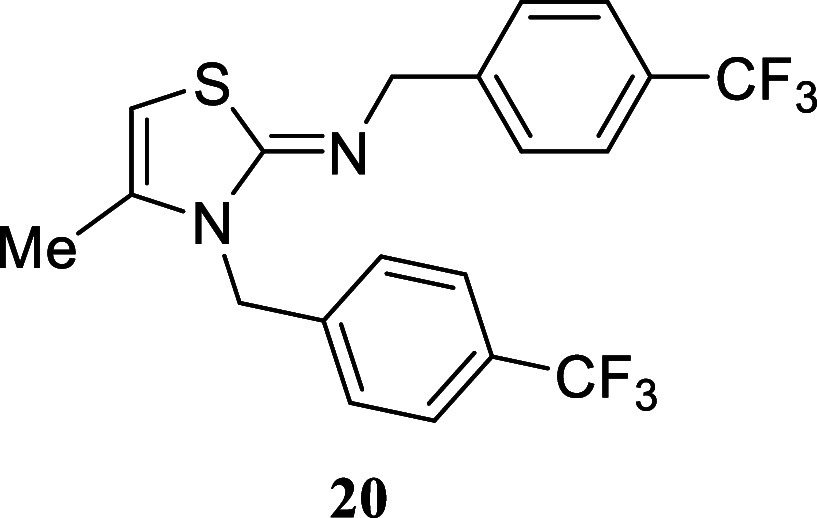


#### 4-Methyl-*N*,3-*bis*(4-(trifluoromethyl)­benzyl)­thiazol-2­(3*H*)-imine **(20)**

Yellowish crystals (198.0 mg, 46%), mp 72.0–73.6 °C. Column chromatography eluent: petroleum ether/EtOAc gradient from 9:1 to pure EtOAc. Rf 0.79 (1:19 v/v MeOH/DCM). ^1^H NMR (400 MHz, MeOD) δ 7.62 (d, *J* = 8.1 Hz, 2H), 7.49 (d, *J* = 8.1 Hz, 2H), 7.37–7.32 (m, 4H), 5.84 (q, *J* = 1.2 Hz, 1H), 5.17 (s, 2H), 4.33 (s, 2H), 2.05 (d, *J* = 1.2 Hz, 3H). ^13^C­{^1^H} NMR (100 MHz, MeOD) δ 164.4, 146.5 (q, ^4^ *J*_C–F_ = 1.2 Hz), 143.7 (q, ^4^ *J*_C–F_ = 1.2 Hz), 137.2, 130.7 (q, ^2^ *J*_C–F_ = 32.3 Hz), 129.9 (q, ^2^ *J*_C–F_ = 32.2 Hz), 129.0, 128.2, 126.7 (q, ^3^ *J*_C–F_ = 3.8 Hz), 126.1 (q, ^3^ *J*_C–F_ = 3.9 Hz), 126.0 (q, ^1^ *J*_C–F_ = 270.9 Hz), 125.8 (q, ^1^ *J*_C–F_ = 271.1 Hz), 94.4, 58.0, 47.6, 14.7. ^19^F­{^1^H} NMR (376 MHz, CDCl_3_) δ −63.77, −63.87. HRMS (ESI) *m*/*z*: [M + H]^+^ Calcd for C_20_H_17_N_2_F_6_S 431.1017, Found 431.1026. IR (ATR, cm^–1^): 2793, 1625, 1603, 1583, 1417, 1400, 1320, 1307, 1157, 1118, 1109, 1064, 1047, 1016, 870, 819, 753, 730, 684, 583.
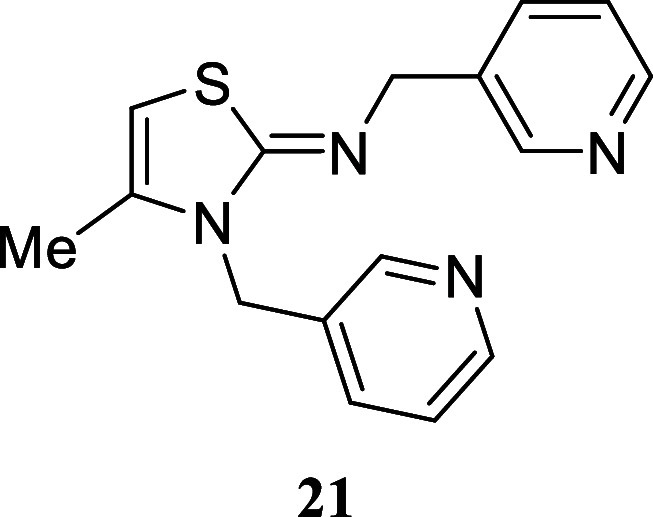


#### 4-Methyl-*N*,3-*bis­(*pyridin-3-ylmethyl)­thiazol-2­(3*H*)-imine **(21)**

Orange oil (58.6 mg, 20%). Column chromatography eluent: petroleum ether/EtOAc gradient from 9:1 to pure EtOAc. Rf 0.50 (1:9 v/v MeOH/DCM). ^1^H NMR (400 MHz, MeOD) δ 8.46–8.41 (m, 3H), 8.37 (dd, *J* = 5.0, 1.6 Hz, 1H), 7.72–7.64 (m, 2H), 7.40 (ddd, *J* = 7.9, 5.0, 0.8 Hz, 1H), 7.40 (ddd, *J* = 7.8, 5.0, 0.9 Hz, 1H), 5.88 (q, *J* = 1.3 Hz, 1H), 5.15 (s, 2H), 4.30 (s, 2H), 2.10 (d, *J* = 1.3 Hz, 3H). ^13^C­{^1^H} NMR (100 MHz, MeOD) δ 164.2, 149.4, 149.1, 148.8, 148.4, 138.4, 137.6, 137.1, 136.8, 135.6, 125.4, 125.2, 94.6, 55.7, 45.6, 14.7. HRMS (ESI) *m*/*z*: [M + H]^+^ Calcd for C_16_H_16_N_4_S 296.1096, Found 296.1098. IR (ATR, cm^–1^): 3032, 1617, 1591, 1574, 1478, 1422, 1355, 1324, 1224, 1186, 1101, 1027, 862, 786, 710, 632, 611, 530.
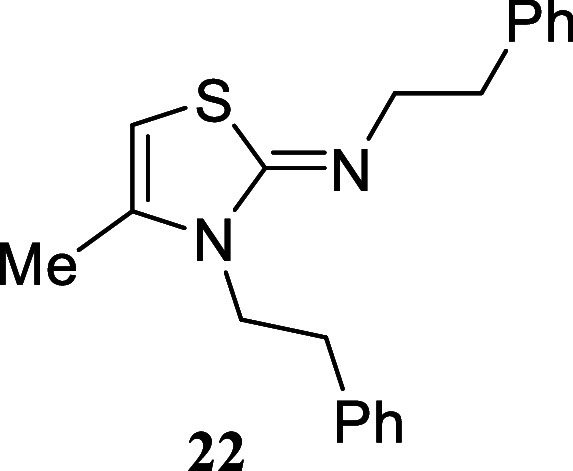


#### 4-Methyl-*N*,3-diphenethylthiazol-2­(3*H*)-imine **(22)**

Yellow oil (309.4 mg, 96%). Column chromatography eluent: petroleum ether/EtOAc gradient from 9:1 to pure EtOAc. Rf 0.50 (1:9 v/v MeOH/DCM). ^1^H NMR (500 MHz, MeOD) δ 7.30–7.13 (m, 8H), 7.11–7.06 (m, 2H), 5.61 (m, 1H), 3.91 (t, *J* = 6.8 Hz, 2H), 3.38 (t, *J* = 7.4 Hz, 2H), 2.96 (t, *J* = 7.4 Hz, 2H), 2.89 (t, *J* = 6.8 Hz, 2H), 1.66 (d, *J* = 1.3 Hz, 3H). ^13^C­{^1^H} NMR (125 MHz, MeOD) δ 163.7, 141.9, 140.4, 137.6, 130.3, 130.1, 129.6, 129.5, 127.7, 127.2, 93.4, 57.4, 46.8, 37.8, 34.4, 14.5. HRMS (ESI) *m*/*z*: [M + H]^+^ Calcd for C_20_H_23_N_2_S 323.1582, Found 323.1600. IR (ATR, cm^–1^): 3061, 3026, 2921, 2856, 1618, 1593, 1496, 1453, 1432, 1401, 1356, 1195, 1155, 1079, 1030, 748, 698, 610, 568, 530, 496.
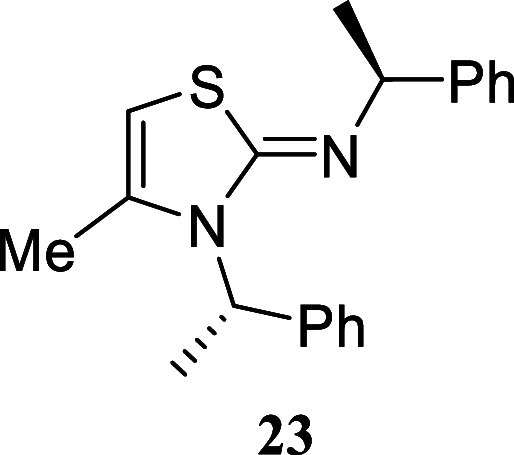


#### 4-Methyl-*N*,3-*bis*((*S*)-1-phenylethyl)­thiazol-2­(3*H*)-imine **(23)**

Colorless oil (258.7 mg, 80%). $\alpha_{[D]}^{20}$ -129.1 (*c* 0.12, MeOH). Column chromatography eluent: petroleum ether/EtOAc gradient from 9:1 to pure EtOAc. Rf 0.50 (1:9 v/v MeOH/DCM). ^1^H NMR (500 MHz, MeOD) δ 7.35–7.29 (m, 4H), 7.28–7.21 (m, 5H), 7.19–7.14 (m, 1H), 6.20 (q, *J* = 7.2 Hz, 1H), 5.61 (q, *J* = 1.1 Hz, 1H), 4.13 (q, *J* = 6.6 Hz, 1H), 1.85 (d, *J* = 7.2 Hz, 3H), 1.74 (d, *J* = 1.1 Hz, 3H), 1.44 (d, *J* = 6.6 Hz, 3H). ^13^C­{^1^H} NMR (125 MHz, MeOD) δ 163.1, 147.6, 142.7, 137.5, 129.7, 129.3, 128.2, 127.7, 127.6, 127.5, 95.0, 64.7, 53.8, 25.4, 17.9, 16.3. HRMS (ESI) *m*/*z*: [M + H]^+^ Calcd for C_20_H_23_N_2_S 323.1582, Found 323.1586. IR (ATR, cm^–1^): 2968, 2923, 1617, 1579, 1493, 1449, 1408, 1311, 1170, 762, 698.
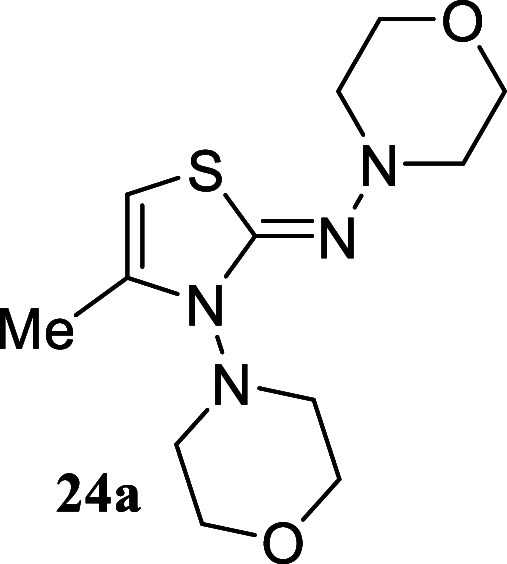


#### 4-Methyl-*N*,3-dimorpholinothiazol-2­(3*H*)-imine **(24a)**

White crystals (51.2 mg, 18%), mp 123.2–125.1 °C. Column chromatography eluent: petroleum ether/EtOAc gradient from 9:1 to pure EtOAc. Rf 0.42 (1:19 v/v MeOH/DCM). ^1^H NMR (500 MHz, MeOD) δ 5.48 (br s, 1H), 4.18 (ddd, *J* = 11.5, 11.5, 2.9 Hz, 2H), 3.87 (d, *J* = 10.7 Hz, 2H), 3.76 (br s, 4H), 3.65 (ddd, *J* = 11.6, 11.6, 2.3 Hz, 2H), 2.75 (br s, 4H), 2.67 (d, *J* = 10.9 Hz, 2H), 2.02 (d, *J* = 1.1 Hz, 3H). ^13^C­{^1^H} NMR (125 MHz, MeOD) δ 166.0, 138.2, 92.7, 68.4, 68.0, 56.7, 51.1, 14.6. HRMS (ESI) *m*/*z*: [M + H]^+^ Calcd for C_12_H_21_N_4_O_2_S 285.1385, Found 285.1387. IR (ATR, cm^–1^): 3080, 2917, 2858, 1574, 1527, 1452, 1431, 1318, 1265, 1109, 1035, 871, 714, 659, 638.
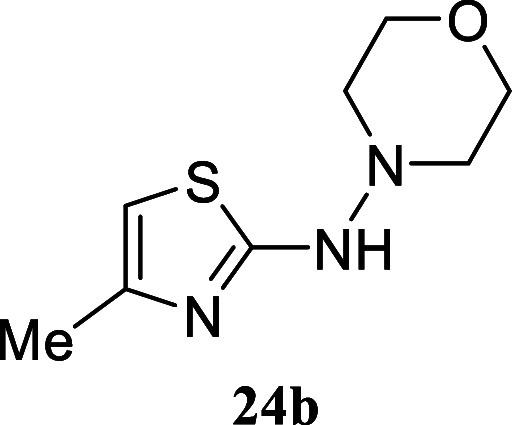


#### *N*-(4-Methylthiazol-2-yl)­morpholin-4-amine **(24b)**

Orange crystals (50.0 mg, 25%), mp 126.5–127.5 °C. Column chromatography eluent: petroleum ether/EtOAc gradient from 9:1 to pure EtOAc. Rf 0.50 (1:9 v/v MeOH/DCM). ^1^H NMR (500 MHz, MeOD) δ 6.20 (q, *J* = 1.1 Hz, 1H), 3.75 (dd, *J* = 4.5, 4.5 Hz, 4H), 2.87 (dd, *J* = 4.5, 4.5 Hz, 4H), 2.15 (d, *J* = 1.1 Hz, 3H). ^13^C­{^1^H} NMR (125 MHz, MeOD) δ 174.9, 149.7, 103.6, 68.3, 56.8, 17.4. HRMS (ESI) *m*/*z*: [M + H]^+^ Calcd for C_8_H_14_N_3_OS 200.0858, Found 200.0871. IR (ATR, cm^–1^): 3096, 2967, 2918, 2857, 2828, 1566, 1442, 1342, 1291, 1260, 1104, 1073, 1023, 993, 951, 915, 876, 853, 725, 658, 725, 622, 610, 513.
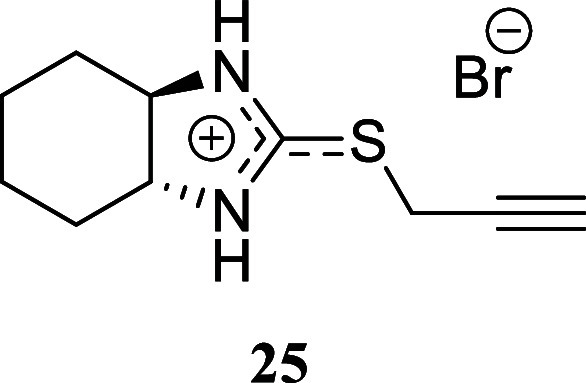


#### *trans*-2-(Prop-2-yn-1-ylthio)-3a,4,5,6,7,7a-hexahydro-1*H*-benzo­[*d*]­imidazole-1-ium Bromide **(25)**

Brown crystals (165.8 mg, 60%), mp 175.4–176.9 °C. Column chromatography eluent: petroleum ether/EtOAc gradient from 9:1 to pure EtOAc, followed by an EtOAc/MeOH gradient from 19:1 to 9:1. Rf 0.37 (1:19 v/v MeOH/DCM). ^1^H NMR (500 MHz, MeOD) δ 3.90 (dd, *J* = 16.6, 2.7 Hz, 1H), 3.83 (dd, *J* = 16.6, 2.7 Hz, 1H), 3.15–3.11 (m, 2H), 2.74 (t, *J* = 2.7 Hz, 1H), 2.19 (dddd, *J* = 13.4, 3.7, 1.7, 1.7 Hz, 2H), 1.88–1.78 (m, 2H), 1.56–1.43 (m, 2H), 1.43–1.33 (m, 2H). ^13^C­{^1^H} NMR (125 MHz, MeOD) δ 169.1, 79.1, 73.9, 70.2, 31.2, 25.7, 19.9. HRMS (ESI) *m*/*z*: [M]^+^ Calcd for C_10_H_15_N_2_S 195.0956, Found 195.0964; [^79^Br]^−^ Calcd for ^79^Br 78.9183, Found 78.9177; [^81^Br]^−^ Calcd for ^81^Br 80.9163, Found 80.9159. IR (ATR, cm^–1^): 3210, 3067, 3011, 2938, 2905, 2860, 1674, 1523, 1506, 1373, 1357, 1258, 1227, 1142, 1100, 710, 691, 641, 615.
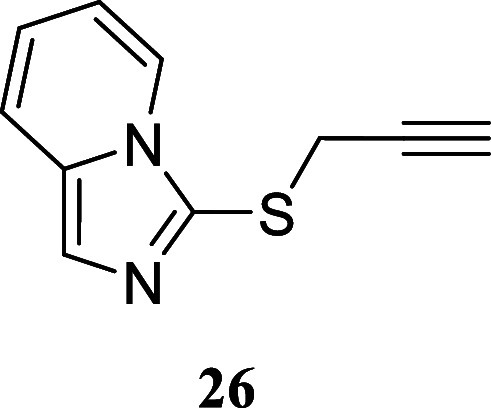


#### 3-(Prop-2-Yn-1-ylthio)­imidazo­[1,5-*a*]­pyridine **(26)**

Brown oil (133.7 mg, 71%). Column chromatography eluent: petroleum ether/EtOAc gradient from 9:1 to pure EtOAc. Rf 0.72 (1:9 v/v MeOH/DCM). ^1^H NMR (500 MHz, MeOD) δ 8.47 (dddd, *J* = 7.2, 1.0, 1.0, 1.0 Hz, 1H), 7.53 (ddd, *J* = 9.1, 1.1, 1.1 Hz, 1H), 7.51 (d, *J* = 0.9 Hz, 1H), 6.89 (ddd, *J* = 9.1, 6.5, 0.9 Hz, 1H), 6.76 (ddd, *J* = 7.2, 6.5, 1.2 Hz, 1H), 3.62 (d, *J* = 2.7 Hz, 2H), 2.49 (t, *J* = 2.7 Hz, 1H). ^13^C­{^1^H} NMR (125 MHz, MeOD) δ 135.0, 129.1, 125.9, 124.3, 122.3, 119.5, 115.1, 80.2, 74.3, 24.8. HRMS (ESI) *m*/*z*: [M + H]^+^ Calcd for C_10_H_9_N_2_S 189.0486, Found 189.0492. IR (ATR, cm^–1^): 3289, 2916, 1633, 1506, 1398, 1352, 1244, 1228, 1015, 795, 738, 667, 646, 564, 448, 423.
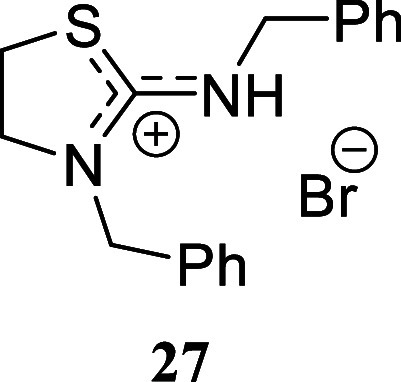


#### 3-Benzyl-2-(benzylamino)-4,5-dihydro-3*H*-thiazol-1-ium Bromide **(27)**

Amorphous solid (189.4 mg, 52%). Column chromatography eluent: petroleum ether/EtOAc gradient from 9:1 to pure EtOAc, followed by an EtOAc/MeOH gradient from 19:1 to 9:1. Rf 0.50 (1:9 v/v MeOH/DCM). ^1^H NMR (500 MHz, MeOD) δ 7.50–7.26 (m, 10H), 4.82 (s, 2H), 4.61 (s, 2H), 3.95 (t, *J* = 7.6 Hz, 2H), 3.51 (t, *J* = 7.6 Hz, 2H). ^13^C­{^1^H} NMR (125 MHz, MeOD) δ 172.1 (from HMBC), 137.7, 135.2, 130.4, 130.0, 129.7, 129.3, 128.92, 128.91, 55.9, 54.2, 51.8, 29.3. HRMS (ESI) *m*/*z*: [M]^+^ Calcd for C_17_H_19_N_2_S 283.1269, Found 283.1272; [^79^Br]^−^ Calcd for ^79^Br 78.9183, Found 78.9181; [^81^Br]^−^ Calcd for ^81^Br 80.9163, Found 80.9160. IR (ATR, cm^–1^) 2994, 2949, 2907, 2828, 1623, 1495, 1438, 1351, 1228, 1147, 747, 719, 697, 666, 626, 608, 553, 529, 459.
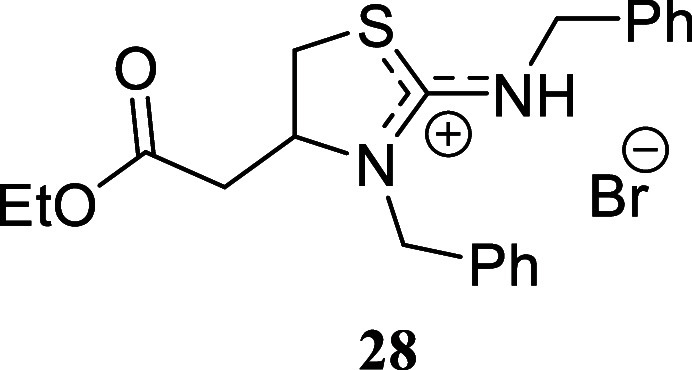


#### 3-Benzyl-2-(benzylamino)-4-(2-ethoxy-2-oxoethyl)-4,5-dihydro-3*H*-thiazol-1-ium Bromide **(28)**

Yellow oil (269.0 mg, 60%). Column chromatography eluent: petroleum ether/EtOAc gradient from 9:1 to pure EtOAc, followed by an EtOAc/MeOH gradient from 19:1 to 9:1. Rf 0.63 (1:9 v/v MeOH/DCM). ^1^H NMR (500 MHz, MeOD) δ 7.35–7.23 (m, 9H), 7.22–7.17 (m, 1H), 4.99 (d, *J* = 15.7 Hz, 1H), 4.43 (s, 2H), 4.28 (d, *J* = 15.7 Hz, 1H), 4.14–4.08 (m, 1H), 4.08 (qd, *J* = 7.1, 1.2 Hz, 2H), 3.43 (ddd, *J* = 11.3, 6.8, 0.4 Hz, 1H), 3.11 (dd, *J* = 11.3, 3.3 Hz, 1H), 2.70 (dd, *J* = 15.7, 4.1 Hz, 1H), 2.63 (dd, *J* = 15.7, 8.6 Hz, 1H), 1.21 (t, *J* = 7.1 Hz, 3H). ^13^C­{^1^H} NMR (125 MHz, MeOD) δ 172.3, 163.2, 141.8, 138.7, 129.8, 129.41, 129.40, 128.8, 128.6, 127.9, 62.1, 59.5, 59.2, 49.0, 36.6, 33.3, 14.6. HRMS (ESI) *m*/*z*: [M]^+^ Calcd for C_21_H_25_N_2_O_2_S 369.1637, Found 369.1653; [^79^Br]^−^ Calcd for ^79^Br 78.9183, Found 78.9174; [^81^Br]^−^ Calcd for ^81^Br 80.9163, Found 80.9152. IR (ATR, cm^–1^): 3029, 2981, 1727, 1628, 1601, 1518, 1495, 1452, 1398, 1375, 1312, 1232, 1183, 1026, 698, 718, 670, 458.
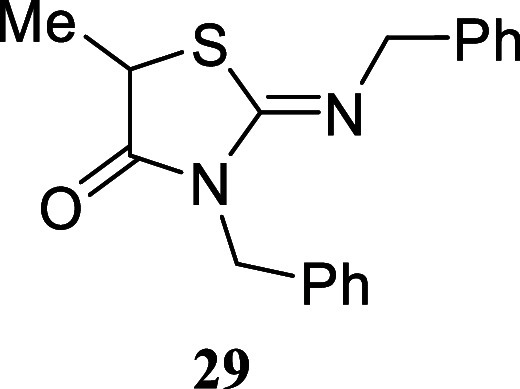


#### 3-Benzyl-2-(benzylimino)-5-methylthiazolidin-4-one **(29)**

Colorless oil (74.5 mg, 24%). Column chromatography eluent: petroleum ether/EtOAc gradient from 9:1 to pure EtOAc. Rf 0.93 (1:9 v/v MeOH/DCM). ^1^H NMR (400 MHz, CDCl_3_) δ 7.46–7.39 (m, 2H), 7.34–7.22 (m, 8H), 4.98 (d, *J* = 14.0 Hz, 1H), 4.92 (d, *J* = 14.0 Hz, 1H), 4.49 (s, 2H), 4.12 (q, *J* = 7.2 Hz, 1H), 1.66 (d, *J* = 7.2 Hz, 3H). ^13^C­{^1^H} NMR (100 MHz, CDCl_3_) δ 175.0, 151.7, 139.4, 136.4, 128.8, 128.4, 128.3, 127.6, 127.3, 126.8, 55.5, 46.1, 42.5, 19.6. HRMS (ESI) *m*/*z*: [M + H]^+^ Calcd for C_18_H_19_N_2_OS 311.1218, Found 311.1223. IR (ATR, cm^–1^): 3032, 2933, 1715, 1638, 1583, 1516, 1450, 1423, 1385, 1339, 1270, 1205, 1154, 1062, 1026, 965, 734, 697, 509.
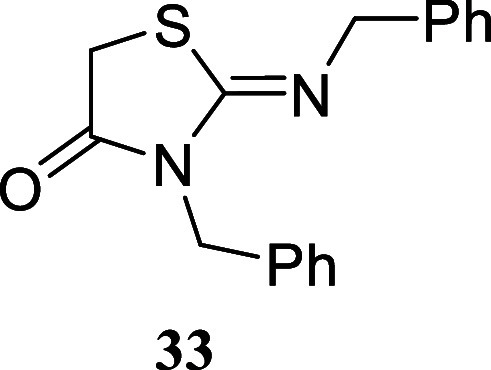


#### 3-Benzyl-2-(benzylimino)­thiazolidin-4-one **(33)** ^32^

White crystals (163.1 mg, 55%), mp 54.0–55.8 °C. Column chromatography eluent: petroleum ether/EtOAc gradient from 9:1 to pure EtOAc. Rf 0.95 (1:9 v/v MeOH/DCM). ^1^H NMR (500 MHz, DMSO-*d*_6_) δ 7.36–7.16 (m, 10H), 4.85 (s, 2H), 4.46 (s, 2H), 4.14 (s, 2H). ^13^C­{^1^H} NMR (125 MHz, DMSO-*d*_6_) δ 171.8, 153.5, 139.4, 136.5, 128.3, 128.2, 127.6, 127.3, 127.1, 126.6, 54.4, 45.2, 32.4. HRMS (ESI) *m*/*z*: [M + H]^+^ Calcd for C_17_H_17_N_2_OS 297.1062, Found 297.1064. IR (ATR, cm^–1^): 3063, 3031, 2976, 2869, 1714, 1643, 1604, 1584, 1496, 1450, 1421, 1376, 1348, 1328, 1317, 1293, 1227, 1027, 757, 722, 702, 691, 665, 606, 582, 558, 513, 493, 454, 432.

### Computational Details

Density functional theory (DFT) calculations were performed with Gaussian 16 (Revision B.01). Geometry optimizations were carried out using the *meta*-hybrid M06–2X functional,^33^ with all elements described using the 6–31+G­(d,p) basis set. Solvent effects (methanol) were included self-consistently in these optimizations through the SMD method.^34^ All stationary points were characterized at this level of theory by analytical frequency calculations as either minima (all positive eigenvalues) or transition states (one imaginary eigenvalue), while intrinsic reaction coordinate (IRC)^35^ calculations and subsequent geometry optimizations were used to confirm the minima linked by each transition state. Those analytical frequency calculations were also used to obtain the corrections required to obtain the free energies at 298 K and 1 atm. In addition, single-point dispersion corrections were computed using Grimme’s D3 (zero damping) parameter set.^36^ Molecular geometries were rendered with CYLView v1.0.^37^

## Supplementary Material



## Data Availability

The data underlying this study are available in the published article and its Suporting Information.

## Notes

Spectroscopic data for all compounds (PDF)

## References

[1] Proskurjakov S. Y., Filimonova M. V., Borovaya O. N., Kucherenko N. G., Trishkina A. I., Shteyn L. V., Skvortsov V. G., Ul’yanova L. P., Shevchenko L. I., Verkhovsky Y. G. (2010). Effect
of NO Inhibitors on Hypovolemic Shock-Induced Hypotension. Bull. Exp. Biol. Med..

[2] Tabari M. A., Vahdati S. A. F., Samakkhah S. A., Araghi A., Youssefi M. R. (2022). Therapeutic
Efficacy of Triclabendazole in Comparison to Combination of Triclabendazole
and Levamisole in Sheep Naturally Infected with Fasciola Sp. J. Parasit Dis..

[3] Veeraselvam M., Sridhar R., Perumal P., Jayathangaraj M. G. (2014). Chemical
Immobilization of Sloth Bears (*Melursus ursinus*)
with Ketamine Hydrochloride and Xylazine Hydrochloride: Hematology
and Serum Biochemical Values. Vet Med. Int..

[4] Nguyen, Q. P. B. ; Kim, T. H. Isothiouronium Organocatalysts Through Hydrogen Bonding. In Recent Advances in Organocatalysis, Karame, I. ; Srour, H. , Eds.; InTech, 2016. DOI:10.5772/62991.

[5] Foreiter M. B., Gunaratne H. Q. N., Nockemann P., Seddon K. R., Stevenson P. J., Wassell D. F. (2013). Chiral Thiouronium Salts: Synthesis, Characterisation
and Application in NMR Enantio-Discrimination of Chiral Oxoanions. New J. Chem..

[6] Nishizawa S., Cui Y.-Y., Minagawa M., Morita K., Kato Y., Taniguchi S., Kato R., Teramae N. (2002). Conversion of Thioureas
to Fluorescent Isothiouronium-Based Photoinduced Electron Transfer
Sensors for Oxoanion Sensing. J. Chem. Soc.,
Perkin Trans. 1.

[7] Lee H., Kang S., Kim T. H. (2018). Isothiouronium Salts as Catalysts
for the Direct Reductive Amination of Aldehydes with a Hantzsch Ester. Bull. Korean Chem. Soc..

[8] Kang S., Yeo H. M., Kim T. H. (2022). *S* -Benzyl- *N,N′* -diphenyl Isothiouronium
Iodide as an Efficient
Organocatalyst for the Transfer Hydrogenation of 1,4-Benzoxazines. Chemistry Select.

[9] Kang S., Kim T. H. (2021). S-Benzyl-N,N’-Diphenyl
Substituted Isothiouronium
Iodide as a Highly Efficient Organocatalyst for Transfer Hydrogenation
of 2-Substituted Quinolines. Tetrahedron Lett..

[10] Nguyen Q. P. B., Kim J. N., Kim T. H. (2012). S-Benzyl
Isothiouronium Chloride
as a Recoverable Organocatalyst for the Reduction of Conjugated Nitroalkenes
with Hantzsch Ester. Tetrahedron.

[11] Yeo H. M., Kang S., Kim T. H. (2021). Isothiouronium Salt-Based Chiral
Proline Amide as Efficient Bifunctional Organocatalyst for Direct
Asymmetric Aldol Reactions in Aqueous Medium. Tetrahedron Lett..

[12] Fujisaki S., Fujiwara I., Norisue Y., Kajigaeshi S. (1985). A Facile One-Pot
Synthesis of Sulfides from Alkyl Halides and Alcohols Using Tetramethylthiourea. Bull. Chem. Soc. Jpn.

[13] Biancalana S., Hudson D., Songster M. F., Thompson S. A. (2000). Fmoc Chemistry Compatible
Thio-Ligation Assembly of Proteins. Lett. Pept.
Sci..

[14] Magné V., Ball L. T. (2019). Synthesis of Air-Stable, Odorless Thiophenol Surrogates
via Ni-Catalyzed CS Cross-Coupling. Chem. –
Eur. J..

[15] Wu S., Melchiorre P. (2024). Photochemical
Synthesis of Thioesters from Aryl Halides
and Carboxylic Acids. Angew. Chem., Int. Ed..

[16] Tiefenbrunner I., Brutiu B. R., Stopka T., Maulide N. (2023). Isothiouronium-Mediated
Conversion of Carboxylic Acids to Cyanomethyl Thioesters. J. Org. Chem..

[17] Castagnolo D., Pagano M., Bernardini M., Botta M. (2009). Domino Alkylation-Cyclization
Reaction of Propargyl Bromides with Thioureas/Thiopyrimidinones: A
New Facile Synthesis of 2-Aminothiazoles and 5H-Thiazolo­[3,2-a]­Pyrimidin-5-Ones. Synlett.

[18] Puig R., Fullana-I-Palmer P., Baquero G., Riba J.-R., Bala A. (2013). A Cumulative
Energy Demand Indicator (CED), Life Cycle Based, for Industrial Waste
Management Decision Making. Waste Manage.

[19] Jin Y., Li J., Peng L., Gao C. (2015). Discovery of Neat Silica Gel as a
Catalyst: An Example of S → O Acetyl Migration Reaction. Chem. Commun..

[20] Stephen A., Hashmi K., Bats J. W., Choi J.-H., Schwarz L. (1998). Isomerizations
on Silica Gel: Synthesis of Allenyl Ketones and the First Nazarov
Cyclizations of Vinyl Allenyl Ketones. Tetrahedron
Lett..

[21] Dhoro F., Tius M. A. (2005). Interrupted Nazarov Cyclization on
Silica Gel. J. Am. Chem. Soc..

[22] dos
Passos Gomes G., Morrison A. E., Dudley G. B., Alabugin I. V. (2019). Optimizing
Amine-Mediated Alkyne–Allene Isomerization to Improve Benzannulation
Cascades: Synergy between Theory and Experiments. Eur. J. Org. Chem..

[23] Wang Y., Hoye T. R. (2018). Isomerizations of
Propargyl 3-Acylpropiolates via Reactive
Allenes. Org. Lett..

[24] Yang H. B., Wei Y. B., Shi M. (2013). Construction
of Spiro­[Indoline]­Oxindoles
through One-Pot Thermal-Induced [3 + 2] Cycloaddition/Silica Gel-Promoted
Fragmentation Sequence between Isatin Ketonitrones and Electron-Deficient
Alkynes. Tetrahedron.

[25] Shao L., Li Y., Shi M. (2007). Silica Gel
Triggered Transformations of 3-Methylenecyclopropylmethyl
Sulfonates to 3-Methylenecyclobutyl Analogues: Experimental and Computational
Studies. Chem. – Eur. J..

[26] Rimola A., Costa D., Sodupe M., Lambert J.-F., Ugliengo P. (2013). Silica Surface
Features and Their Role in the Adsorption of Biomolecules: Computational
Modeling and Experiments. Chem. Rev..

[27] Rimola A., Fabbiani M., Sodupe M., Ugliengo P., Martra G. (2018). How Does Silica
Catalyze the Amide Bond Formation under Dry Conditions? Role of Specific
Surface Silanol Pairs. ACS Catal..

[28] Li D., Yuan X. A., He X., Liu P., Bi S. (2023). Theoretical
Studies on the Mechanisms and Origins of Substituent Effects in CuBr-Catalyzed
Three-Component Tandem Reactions of Terminal Propargyl Alcohols, Aldehydes
and Amines. Mol. Catal..

[29] Hammond G. S. (1955). A Correlation
of Reaction Rates. J. Am. Chem. Soc..

[30] Leffler J. E. (1953). Parameters
for the Description of Transition States. Science.

[31] Kraemer N., Naredla R. R., Hoye T. R. (2022). In Situ Allene Formation via Alkyne
Tautomerization to Promote [4 + 2]-Cycloadditions with a Pendant Alkyne
or Nitrile. Org. Lett..

[32] Mamaghani M., Pourranjbar M., Hossein Nia R. (2014). Facile and Regioselective Synthesis
of Thiazolidin-4-one Derivatives Catalyzed by Basic Ionic Liquid [bmim]­OH
under Ultrasonic Irradiation. J. Sulfur Chem..

[33] Zhao Y., Truhlar D. G. (2008). The M06 Suite of
Density Functionals for Main Group
Thermochemistry, Thermochemical Kinetics, Noncovalent Interactions,
Excited States, and Transition Elements: Two New Functionals and Systematic
Testing of Four M06-Class Functionals and 12 Other Functionals. Theor. Chem. Acc..

[34] Marenich A. V., Cramer C. J., Truhlar D. G. (2009). Universal
Solvation Model Based on
Solute Electron Density and on a Continuum Model of the Solvent Defined
by the Bulk Dielectric Constant and Atomic Surface Tensions. J. Phys. Chem. B.

[35] Fukui K. (1981). The Path of
Chemical Reactions — The IRC Approach. Acc. Chem. Res..

[36] Grimme S., Antony J., Ehrlich S., Krieg H. (2010). A Consistent and Accurate
Ab Initio Parametrization of Density Functional Dispersion Correction
(DFT-D) for the 94 Elements H-Pu. J. Chem. Phys..

[37] Legault, C. Y. CYLview 1.0.; Université de Sherbrooke. 2009.

