# Supplementary material for: Microwave-Assisted One-Pot Synthesis of Isothiouronium Salts: Experimental and DFT Insights into Silica-Promoted Cyclization toward Thiazolidinium and Thiazole Frameworks
Source: J Org Chem. 2025 Sep 5;90(37):12916–30. doi: 10.1021/acs.joc.5c01131 (PMC12455660; doi:10.1021/acs.joc.5c01131)
Supplement: Supplementary file 1 [file jo5c01131_si_001.pdf]

Supporting Information

Microwave-Assisted One-Pot Synthesis of Isothiouronium Salts: Experimental and DFT Insights into Silica-Promoted Cyclization toward Thiazolidinium and Thiazole Frameworks

Pablo Macías-Benítez,[†] Andrés G. Algarra,[‡] F. Javier Moreno-Dorado[†] and Francisco M. Guerra[†*]

[†]*Departamento de Química Orgánica e Instituto de Biomoléculas (INBIO), Facultad de Ciencias, Universidad de Cádiz, Polígono Río San Pedro s/n, 11510 Puerto Real, Cádiz, SPAIN*

[‡]*Departamento de Ciencia de los Materiales e Ingeniería Metalúrgica y Química Inorgánica e Instituto de Biomoléculas (INBIO), Facultad de Ciencias, Universidad de Cádiz, Polígono Río San Pedro s/n, 11510 Puerto Real, Cádiz, SPAIN.*

*E-mail: francisco.guerra@uca.es

Table of Contents

1. Instrumentation and Materials S2
2. MW Methods S3
3. NMR Spectra S4
4. HRMS-ESI of bis-thiouronium salt **13** S38
5. DFT calculation data S39

Instrumentation and Materials

Reagents were purchased from PanReac AppliChem, Sigma-Aldrich, or TCI, and were used without further purification.

Thin-layer chromatography (TLC) was empoyed to monitor reacitons using commercial silica gel plates precoated with silica gel F254. Visualization was achieved by fluorescence quenching or by spraying with ethanolic solutions of cerium ammonium molybdate or anisaldehyde as developing agents.

Column chromatography was carried out using 230-400 mesh silica gel. High-performance liquid chromatography (HPLC) purifications were conducted on a Merck-Hitachi L6270 chromatograph equipped with a silica gel column (LiChrosorb Si 60, 10 μm particle size).

NMR spectra were recorded on Bruker Avance Neo 400 and Bruker Avance Neo 500 instruments. Spectra were calibrated using the residual undeuterated solvent as interna reference for ^{1}H-NMR, and the central peak of $CDCl_3$ for ^{13}C-NMR.

IR spectra were recorded on a Perkin Elmer Spectrum BX spectrophotometer (ATR).

Mass spectra were acquired using a Bruker Scion GC-TQ gras chromatograpm coupled to a Bruker TQ mass spectrometer. High-resolution mass spectra (HRMS) were obtained on a Waters SYNAPT 2G instrument equipped with an APGC interface and QTOF analyzer, or an a Xaters XEVO G2 system using electrospray ionization (ESI).

Microwave-assisted reactions were conducted in a SynthWave MA167 reactor (Milestone) pressurized with nitrogen, with a maximum pressure limited to 45 bar. Reactions were carried out in 50 mL glass vials immersed in 200 mL of water as thermal transfer medium, under magnetic stirring.

1. MW Methods.

Table S1. **Method A**

Nº	t	T_1 – vessel (ºC)	T_2 – system (ºC)	P (bar)	E (W)
1	00:00:10	100	50	35.0	1500
2	00:01:00	100	50	45.0	1500
3	00:09:00	100	50	45.0	1500

Table S2. **Method B (Optimal Conditions – Step 1)**

Nº	t	T_1 – vessel (ºC)	T_2 – system (ºC)	P (bar)	E (W)
1	00:00:10	160	50	35.0	1500
2	00:01:00	160	50	45.0	1500
3	00:09:00	160	50	45.0	1500

Table S3. **Method C (Optimal Conditions – Step 2)**

Nº	t	T_1 – vessel (ºC)	T_2 – system (ºC)	P (bar)	E (W)
1	00:00:10	100	50	35.0	1500
2	00:01:00	100	50	45.0	1500
3	00:14:00	100	50	45.0	1500

2. NMR Spectra

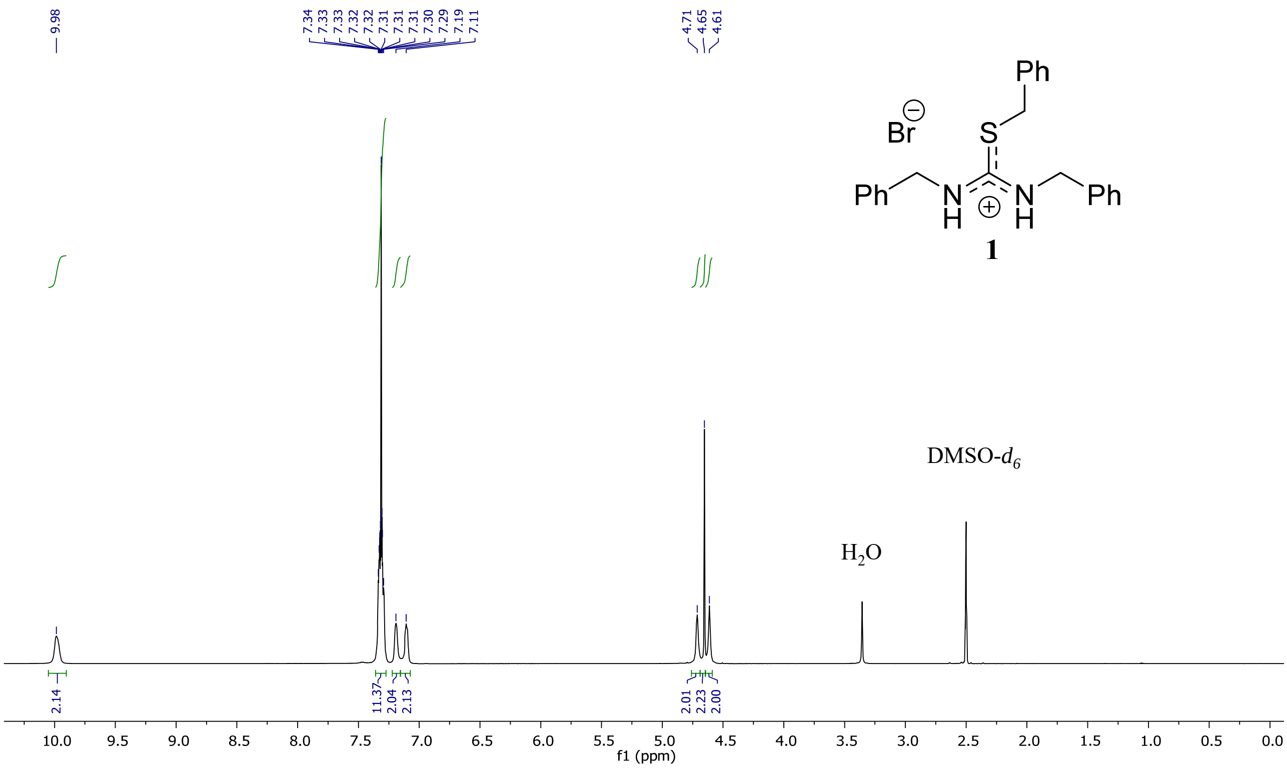

Figure S1. [1]H Spectrum of **1** in DMSO-d_6 (500 MHz)

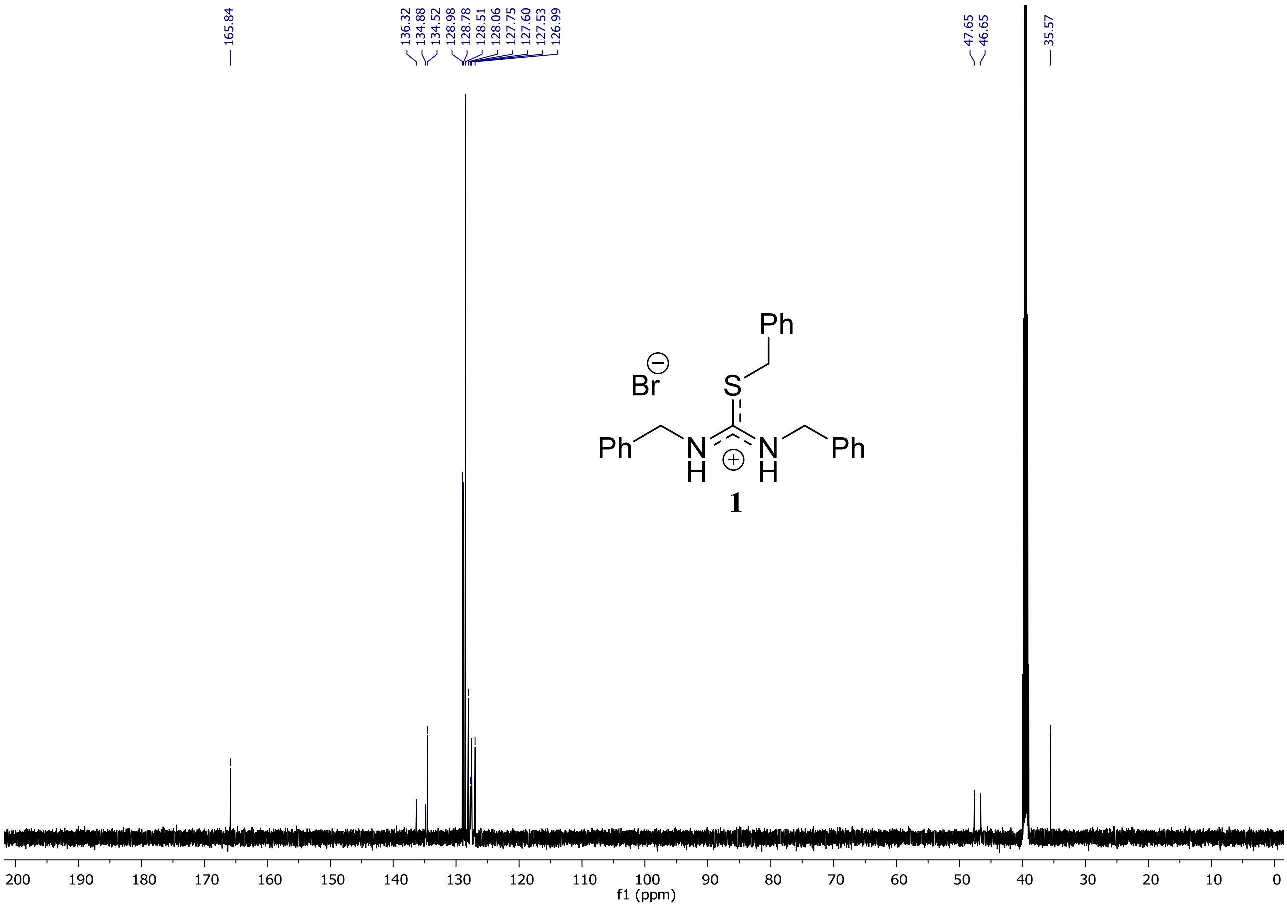

Figure S2. $^{13}C\{^1H\}$ Spectrum of **1** in DMSO-d_6 (125 MHz)

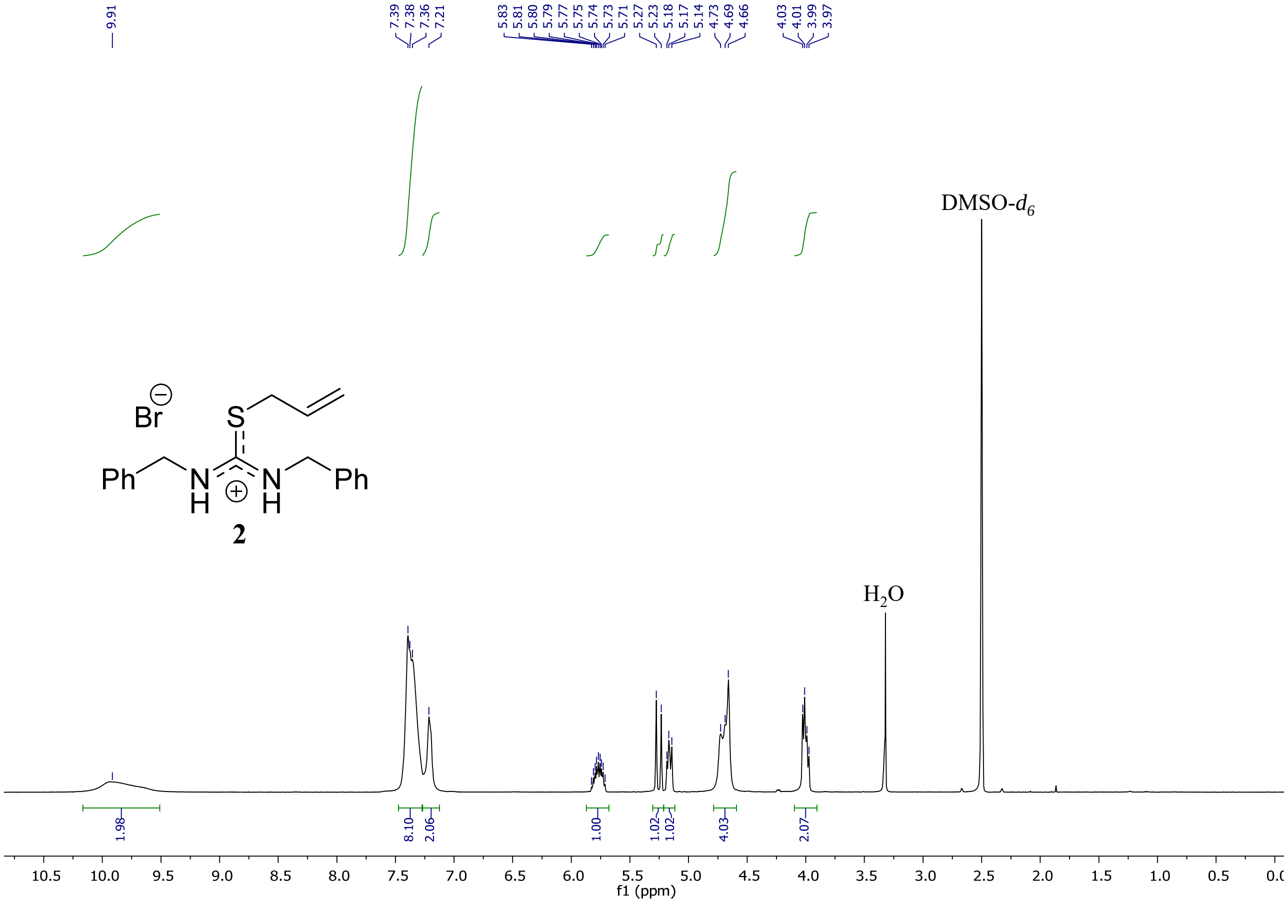

Figure S3. ^1H Spectrum of **2** in DMSO-d_6 (400 MHz)

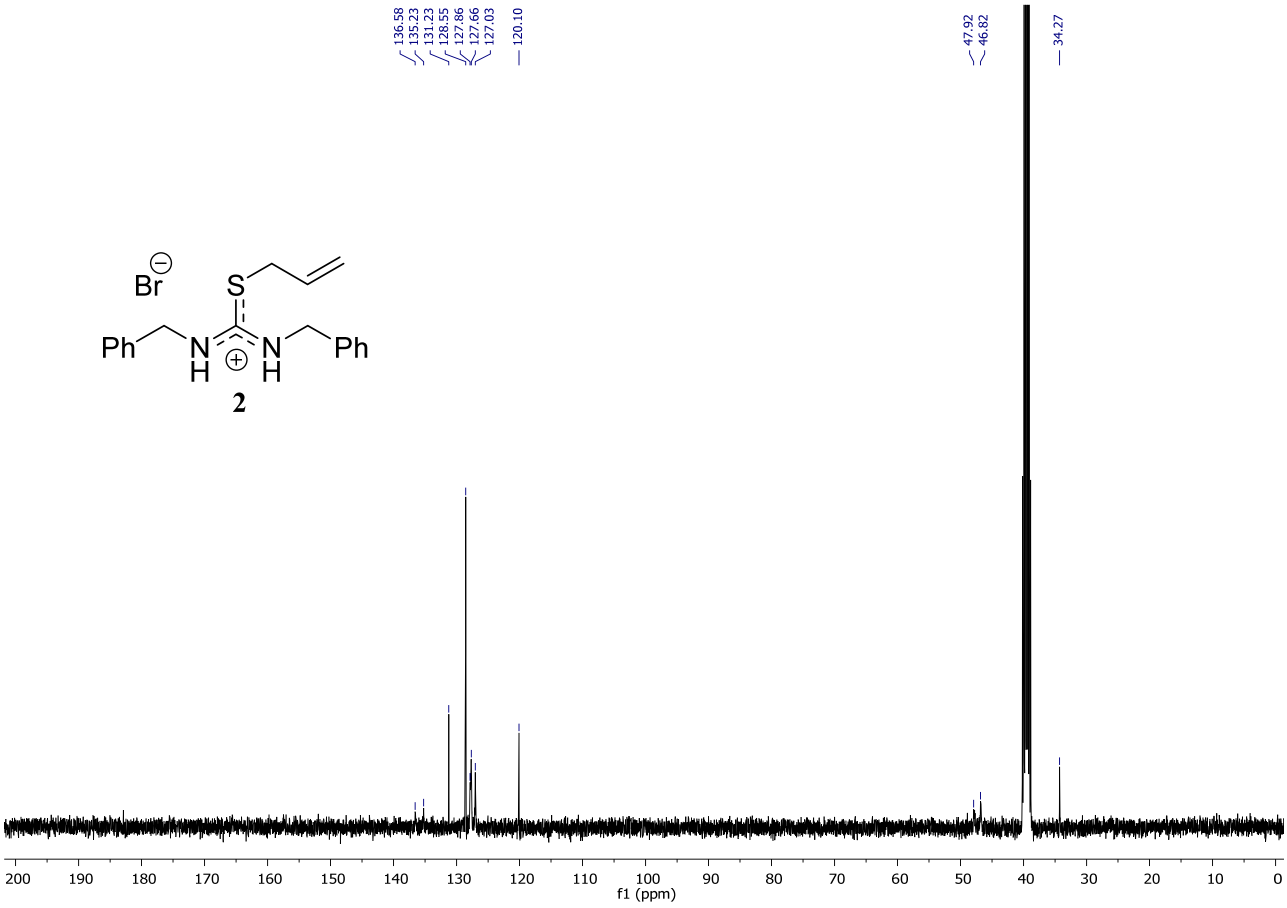

Figure S4. $^{13}C\{^1H\}$ Spectrum of **2** in DMSO-d_6 (100 MHz)

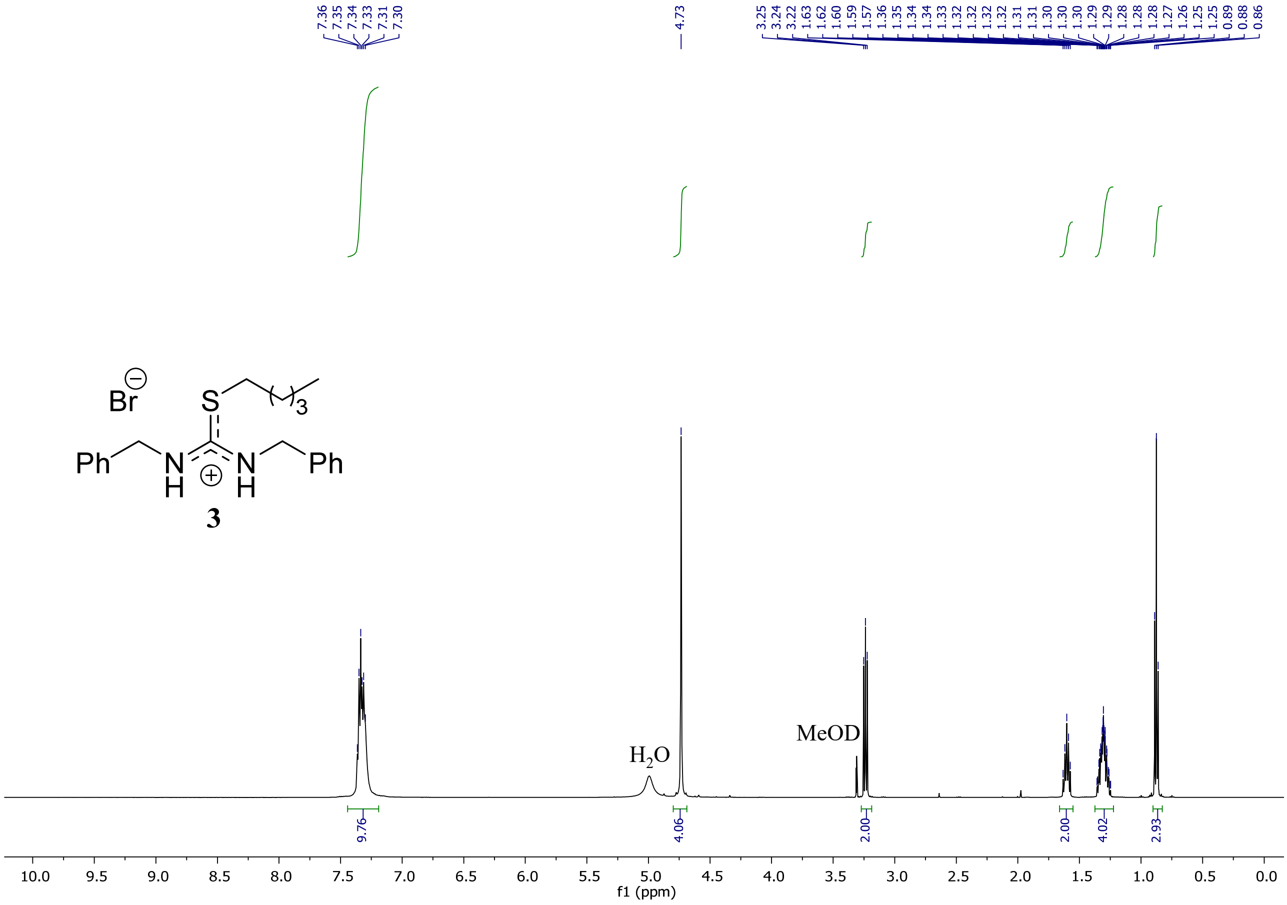

Figure S5. [1]H Spectrum of **3** in MeOD (500 MHz)

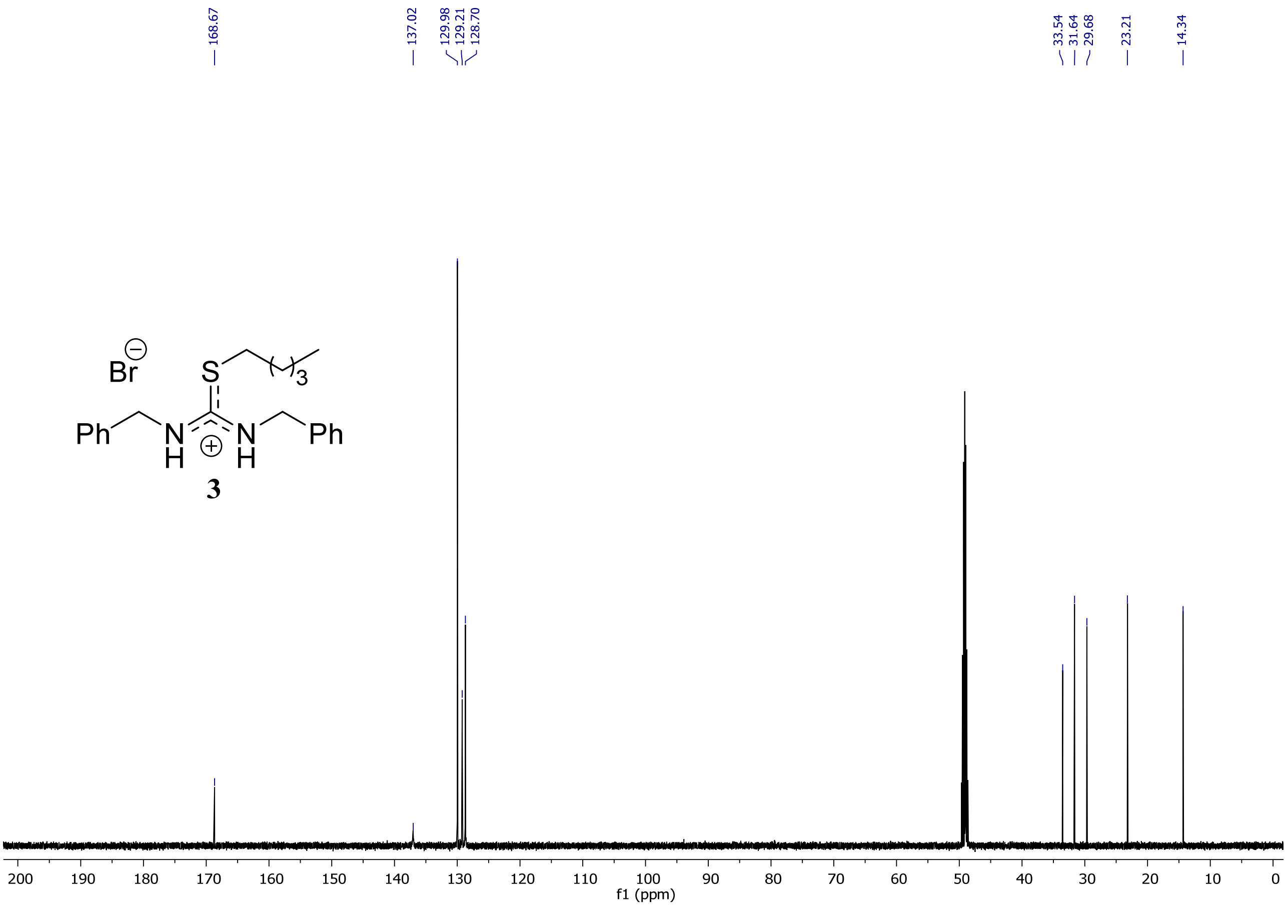

Figure S6. ^{13}C{^{1}H} Spectrum of **3** in MeOD (125 MHz)

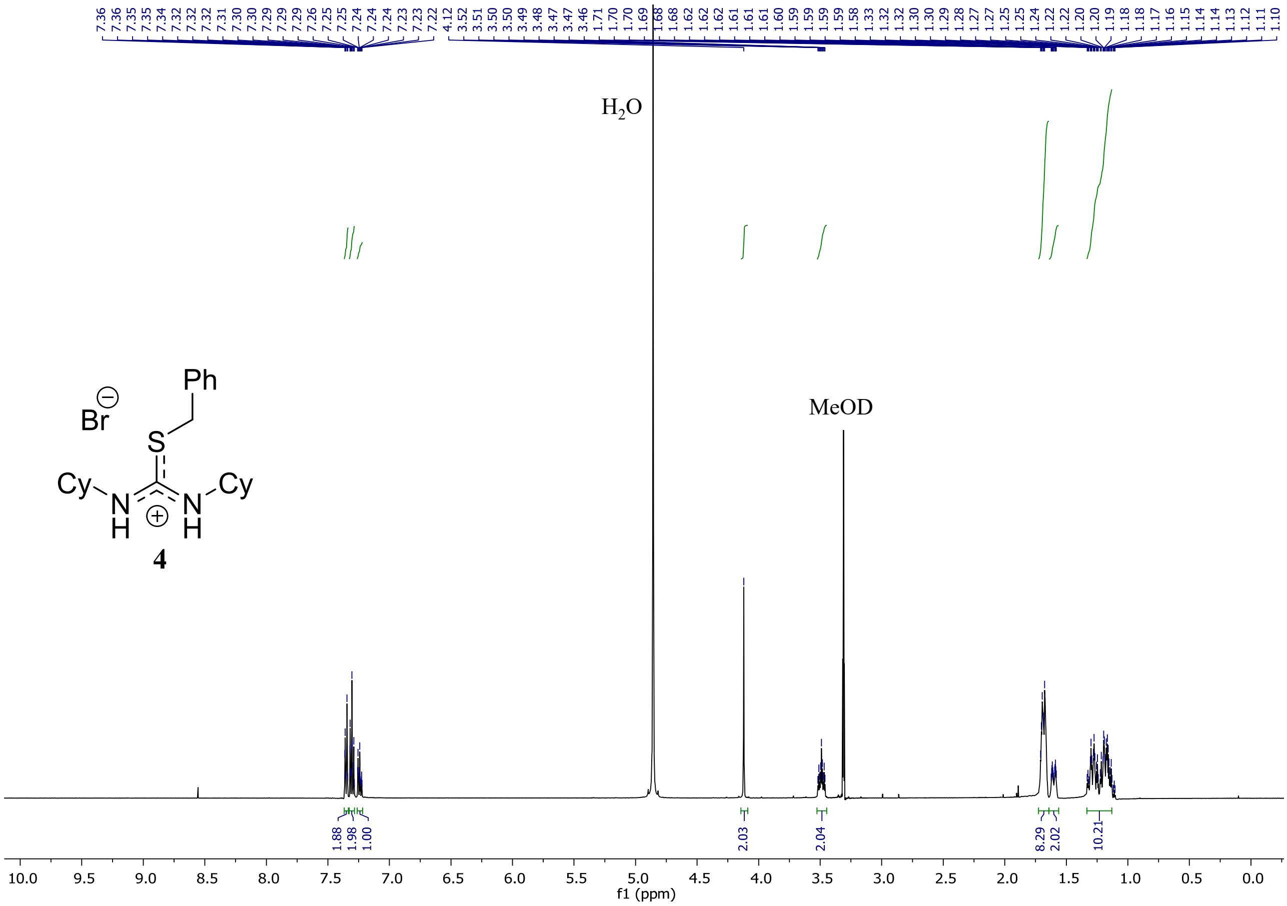

Figure S7. [1]H Spectrum of **4** in MeOD (500 MHz)

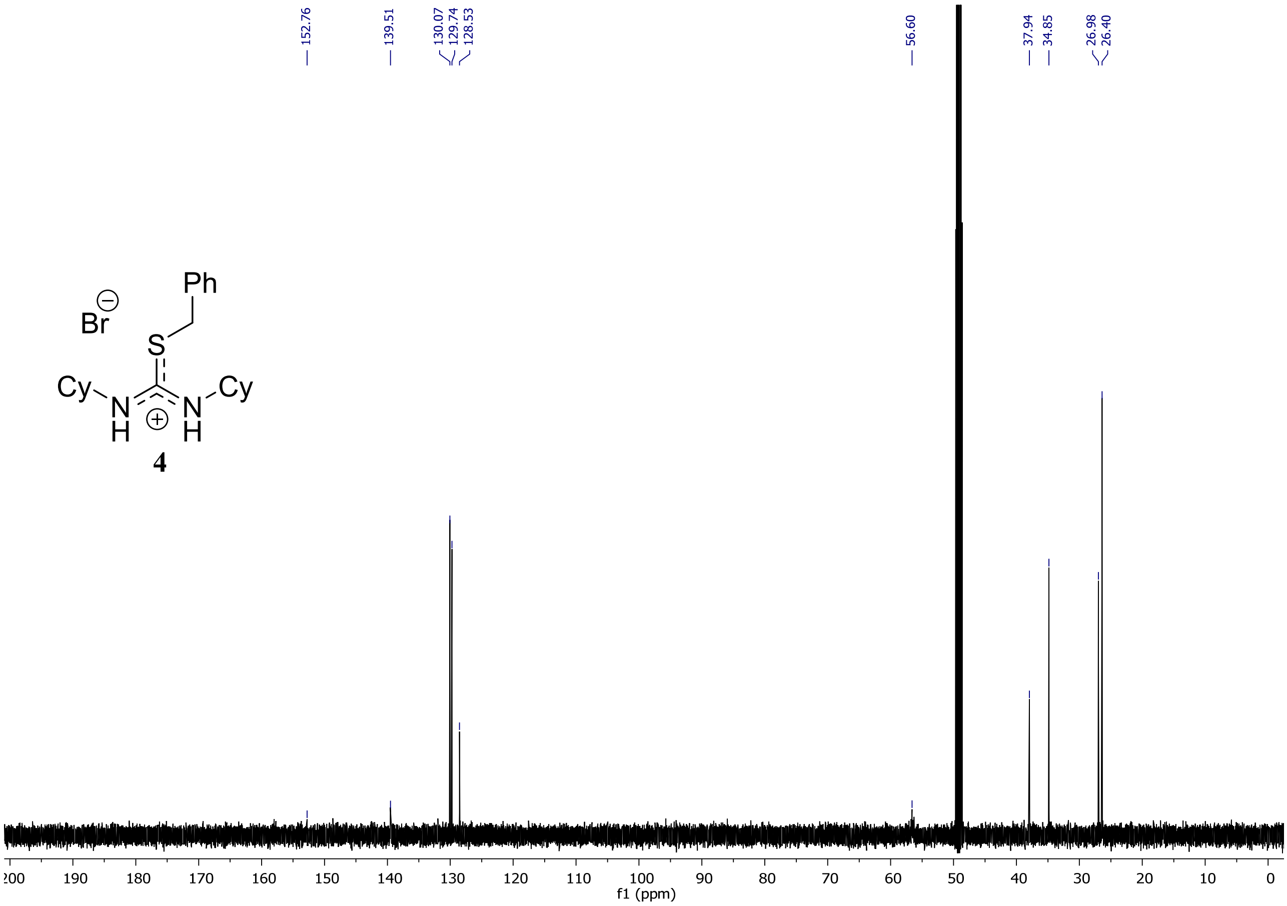

Figure S8. $^{13}C\{^{1}H\}$ Spectrum of **4** in MeOD (125 MHz)

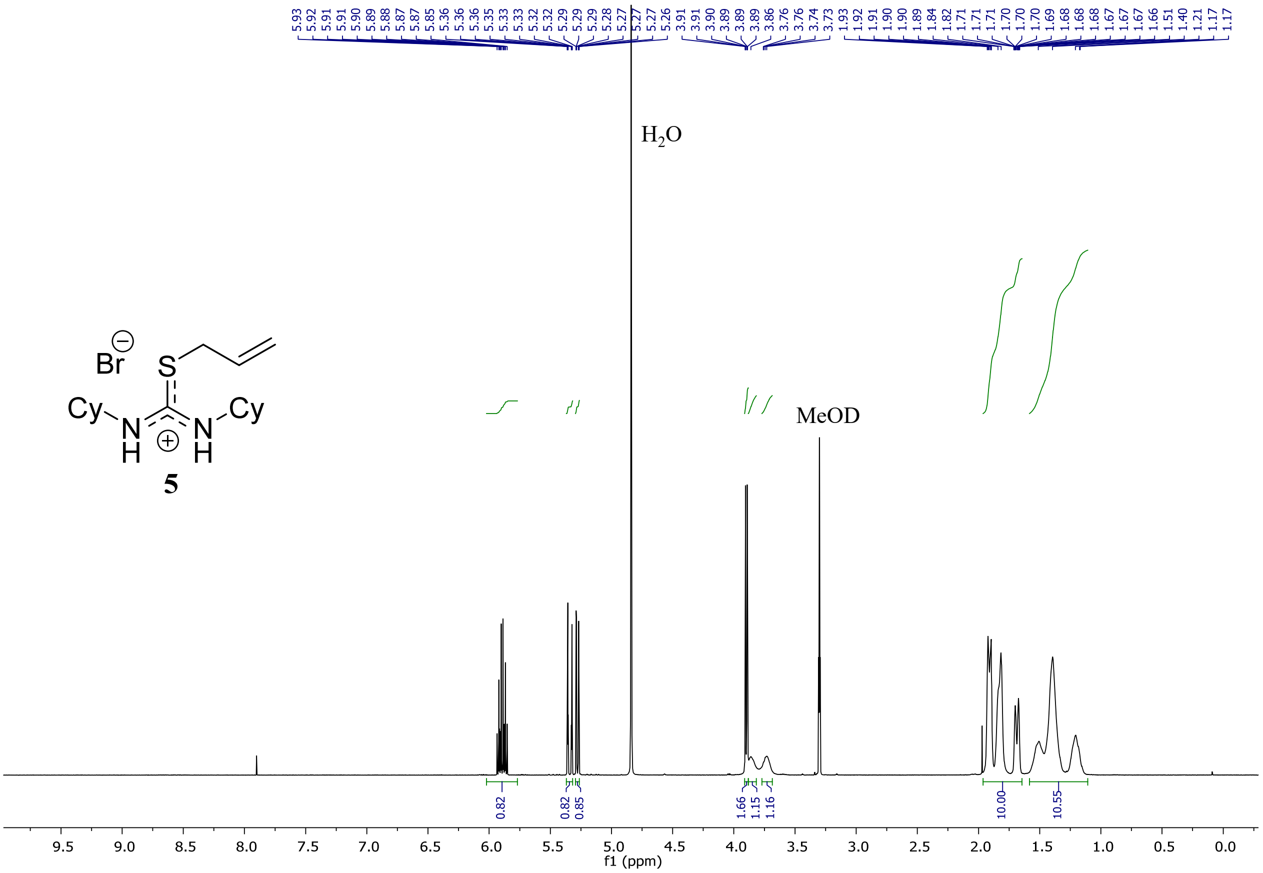

Figure S9. ^{1}H Spectrum of **5** in MeOD (500 MHz)

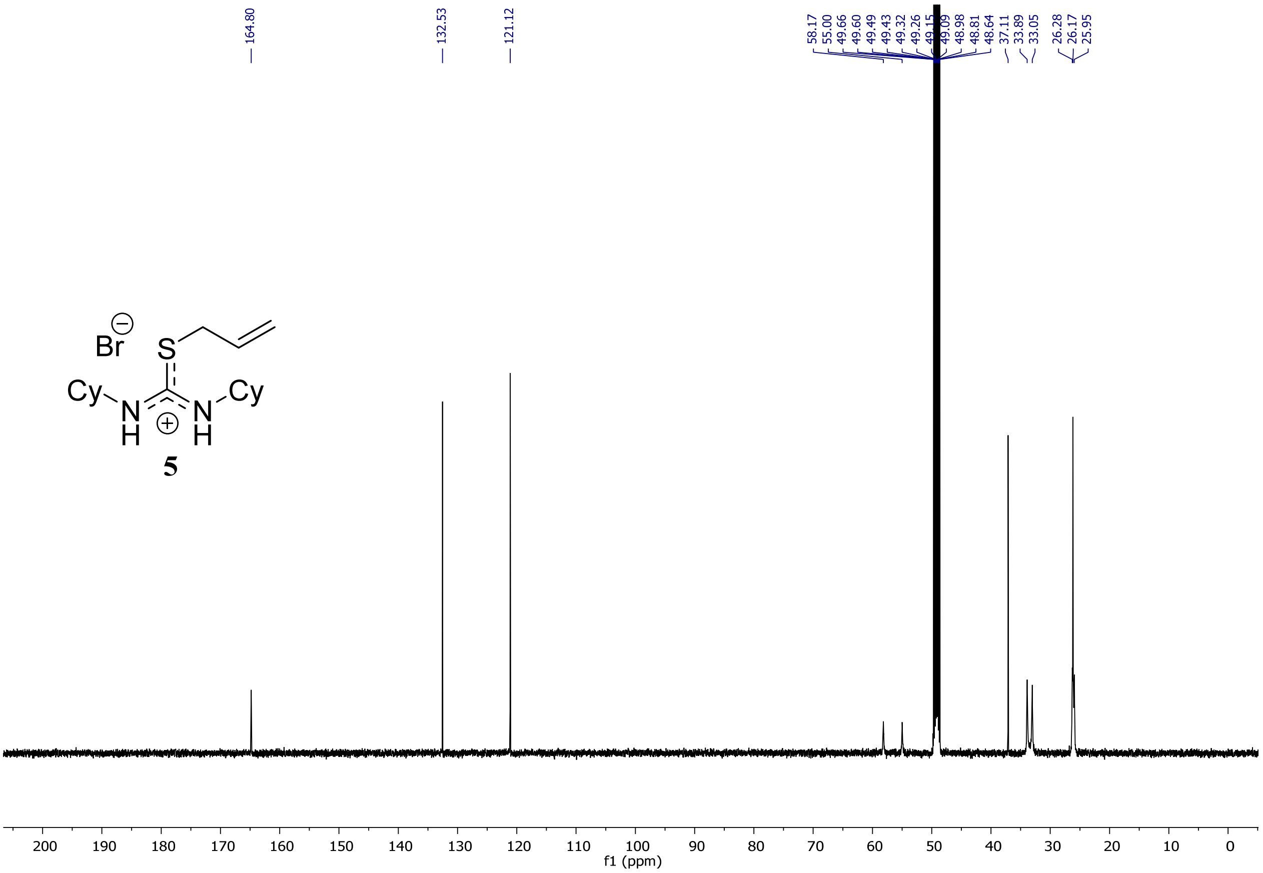

Figure S10. $^{13}C\{^{1}H\}$ Spectrum of **5** in MeOD (125 MHz)

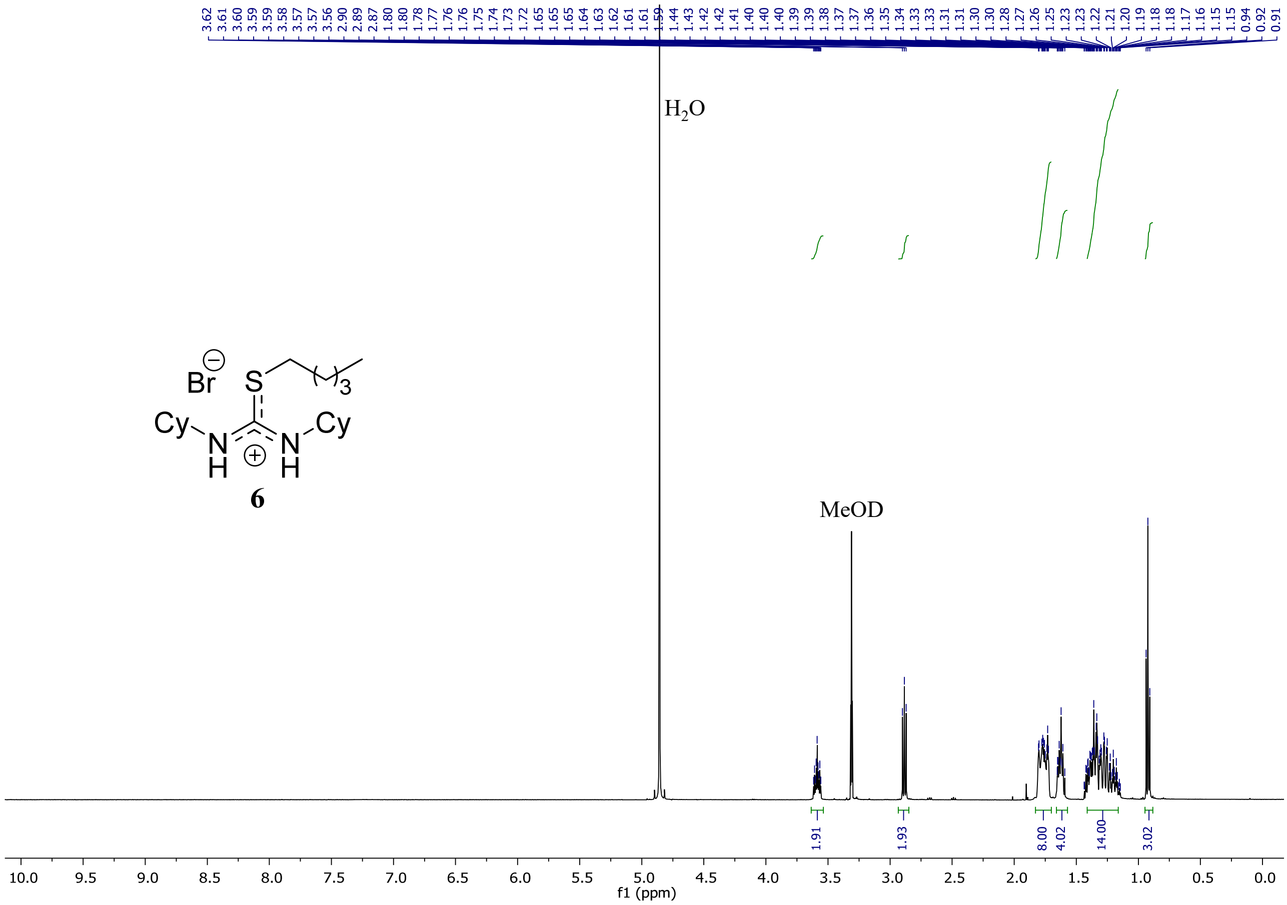

Figure S11. [1]H Spectrum of **6** in MeOD (500 MHz)

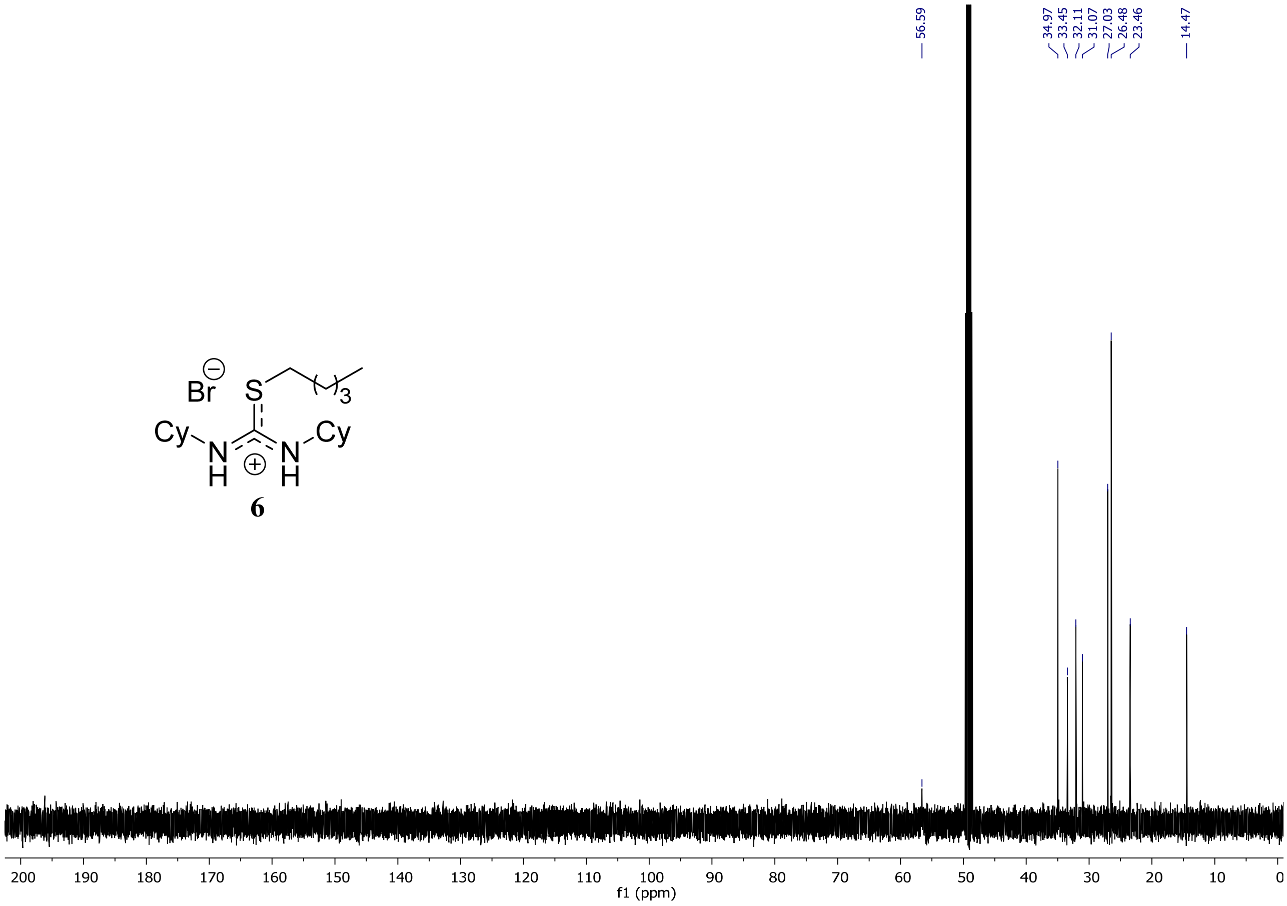

Figure S12. ^{13}C{^{1}H} Spectrum of **6** in MeOD (125 MHz)

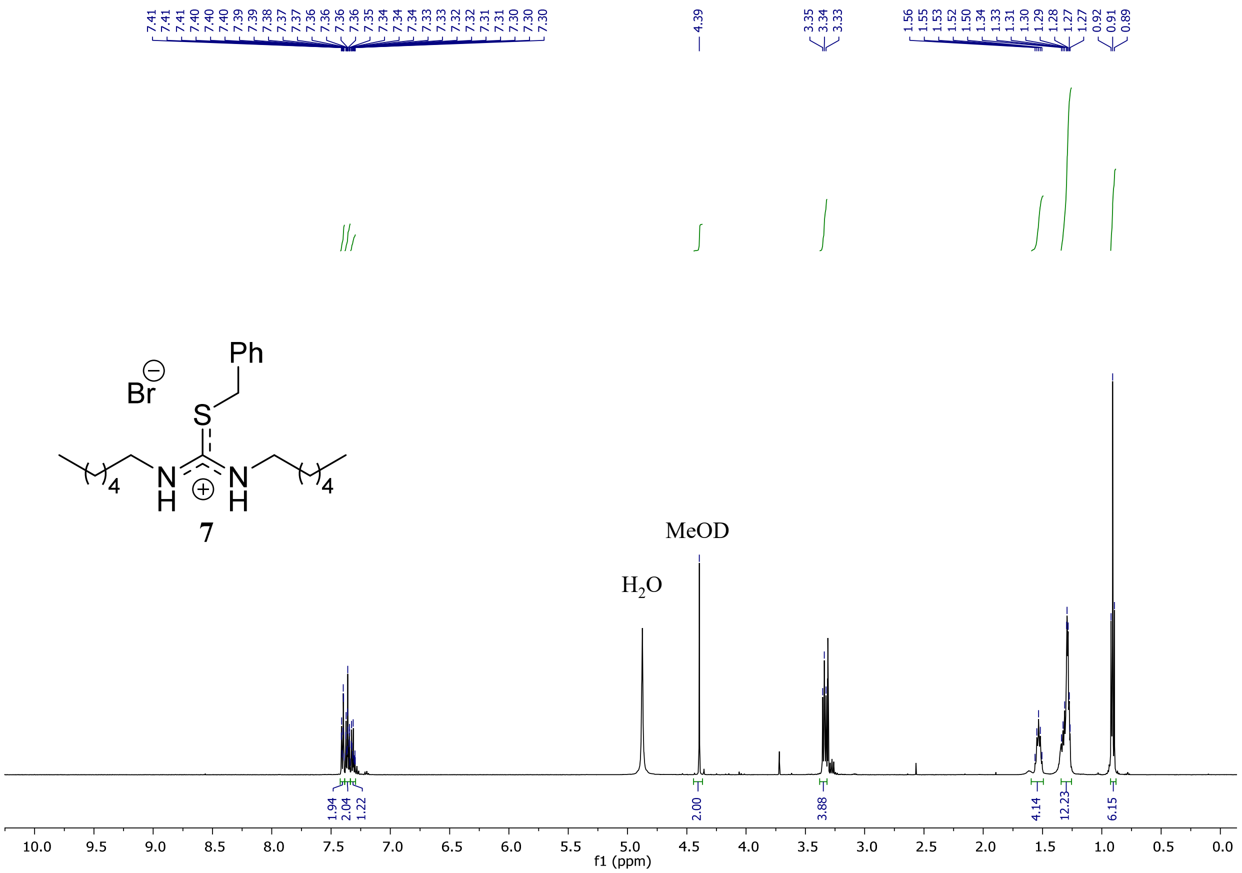

Figure S13. ^{1}H Spectrum of **7** in MeOD (500 MHz)

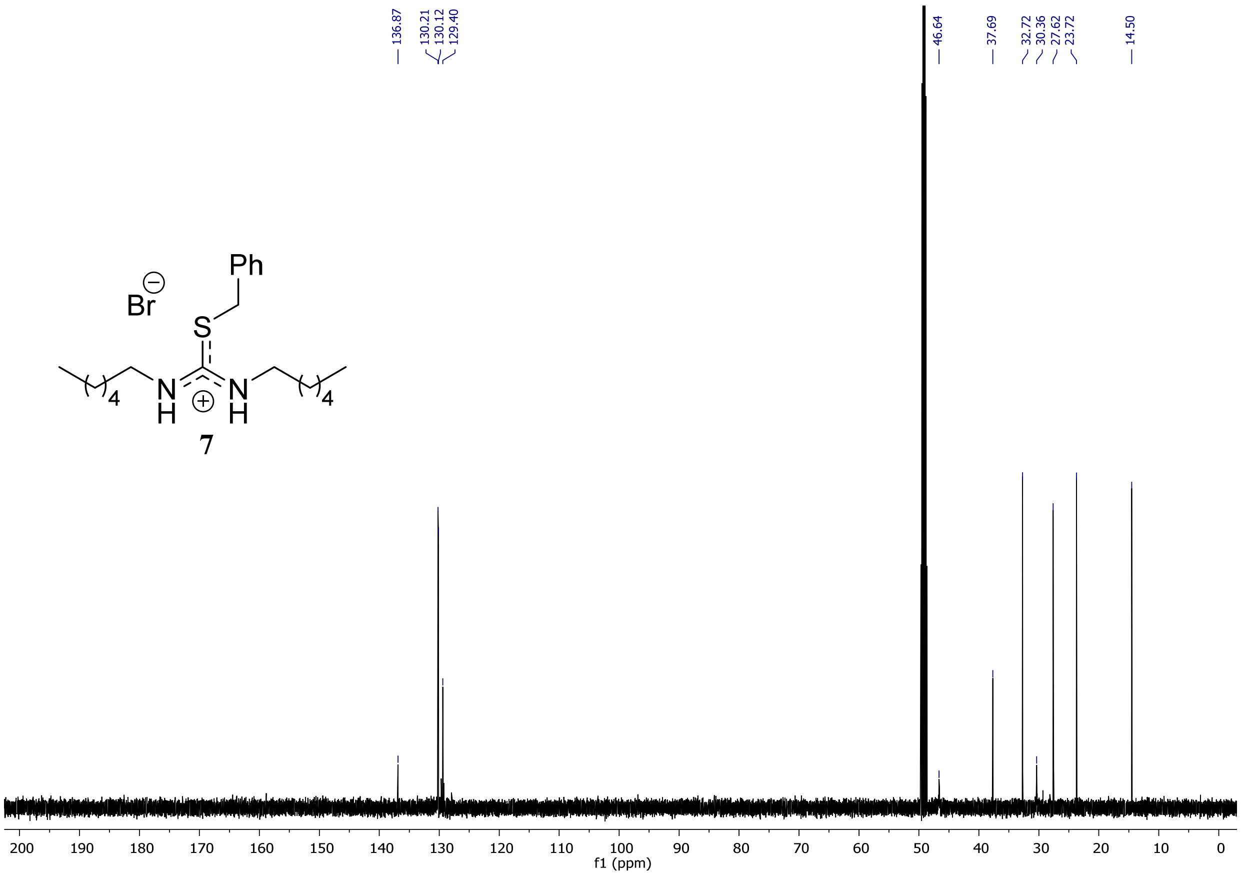

Figure S14. $^{13}C\{^{1}H\}$ Spectrum of **7** in MeOD (125 MHz)

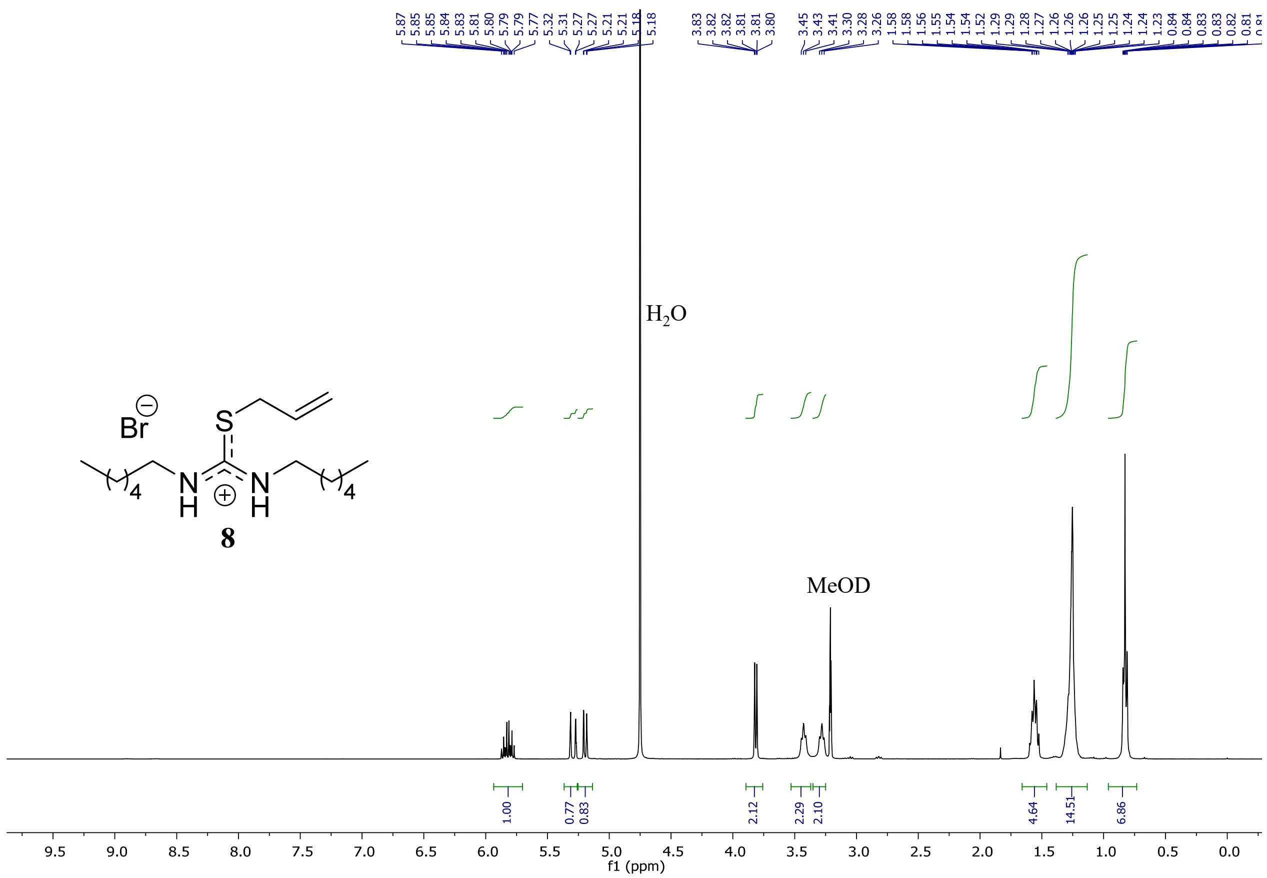

Figure S15. ^{1}H Spectrum of **8** in MeOD (500 MHz)

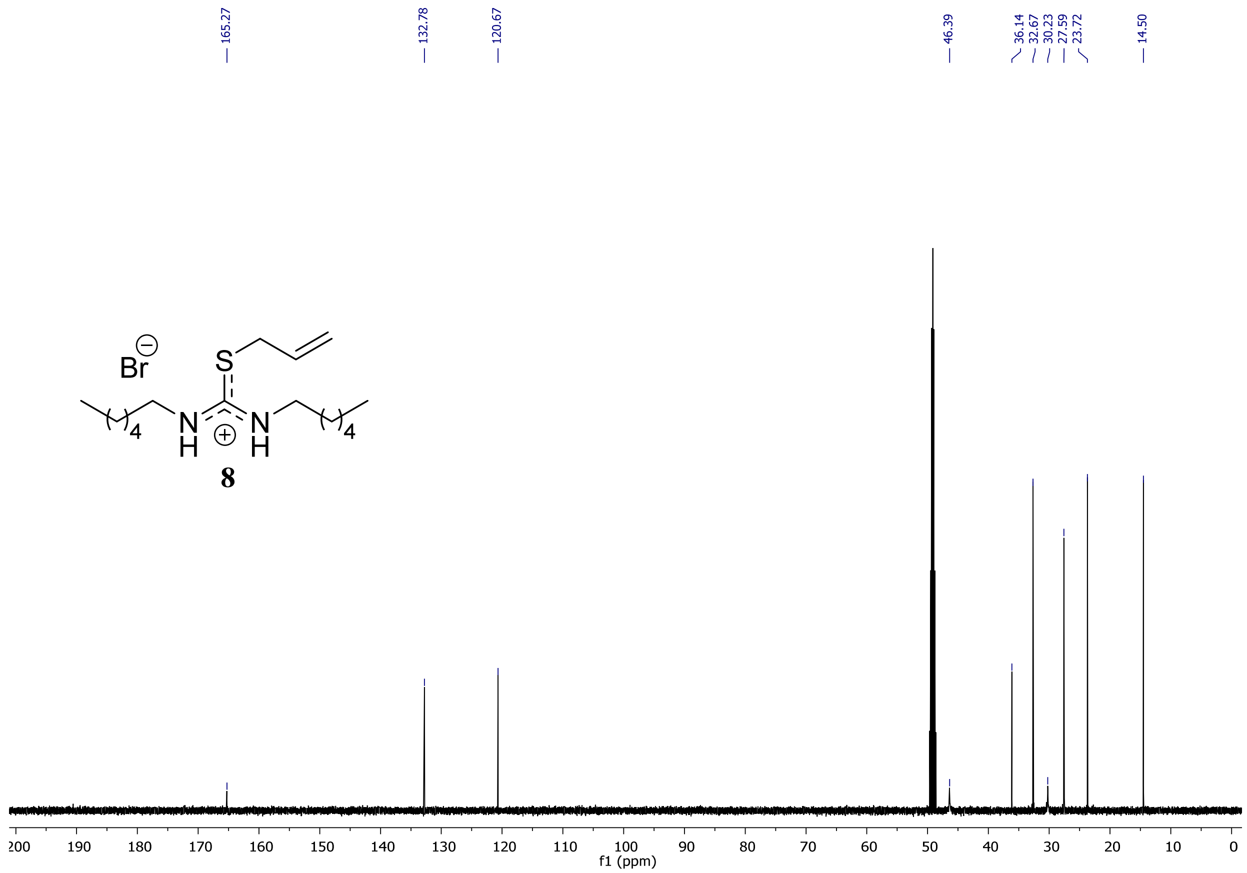

Figure S16. $^{13}C\{^{1}H\}$ Spectrum of **8** in MeOD (125 MHz)

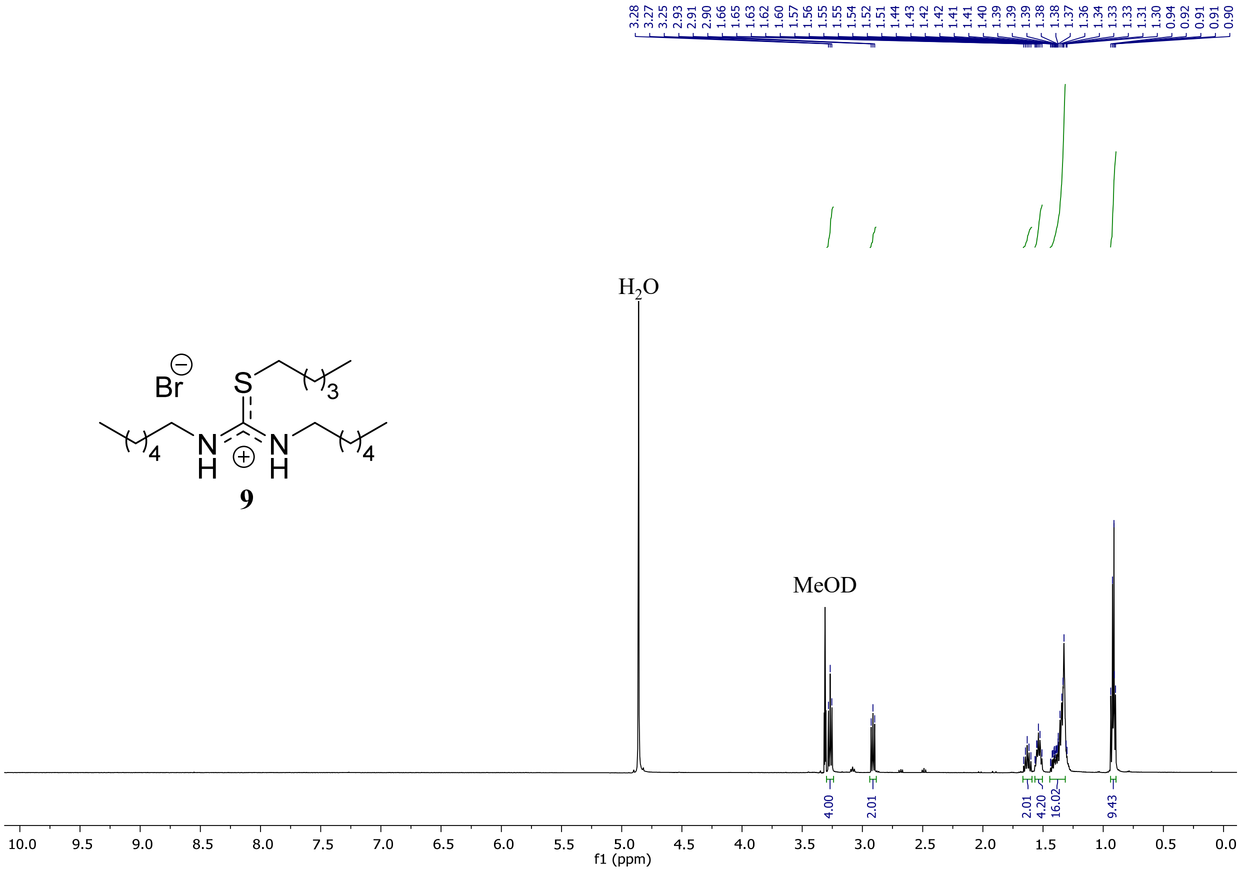

Figure S17. ^{1}H Spectrum of **9** in MeOD (500 MHz)

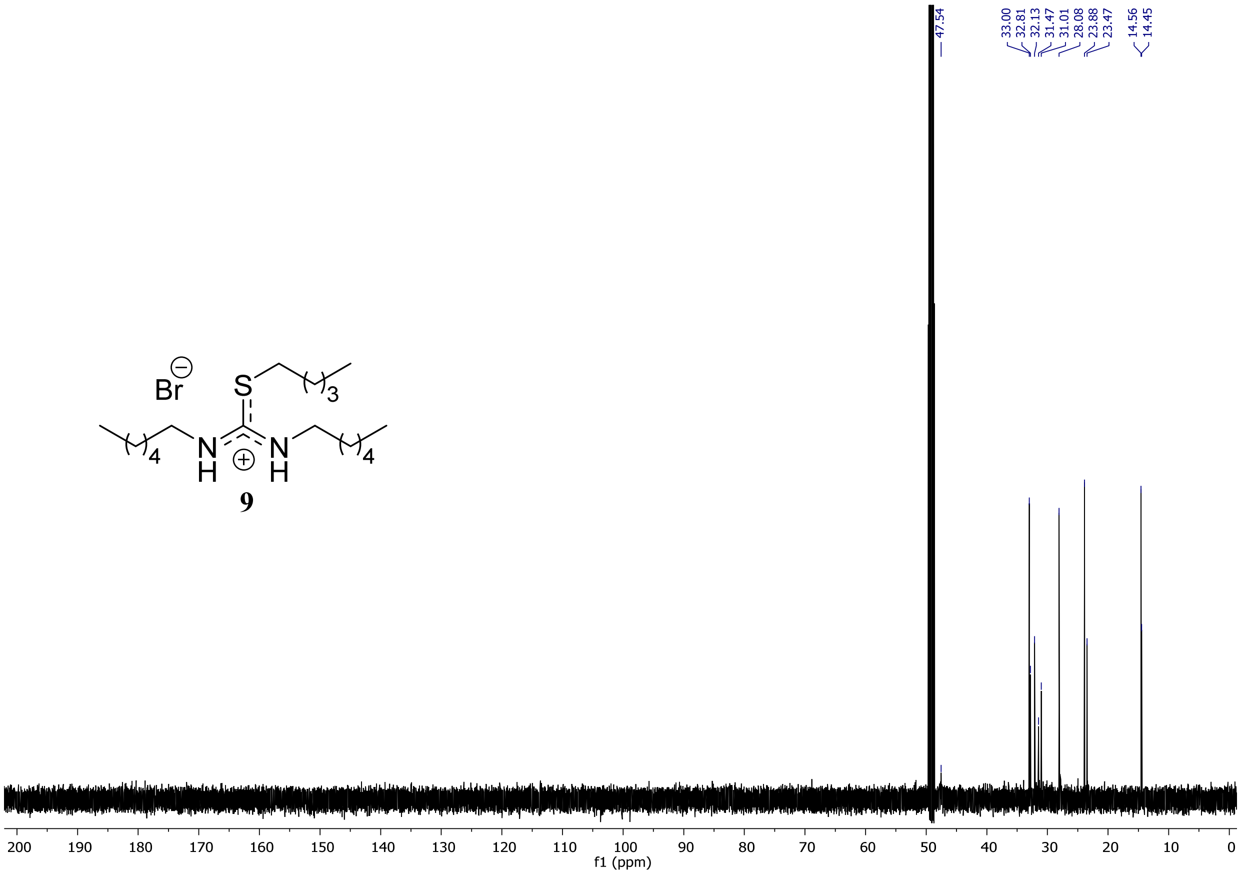

Figure S18. $^{13}C\{^{1}H\}$ Spectrum of **9** in MeOD (125 MHz)

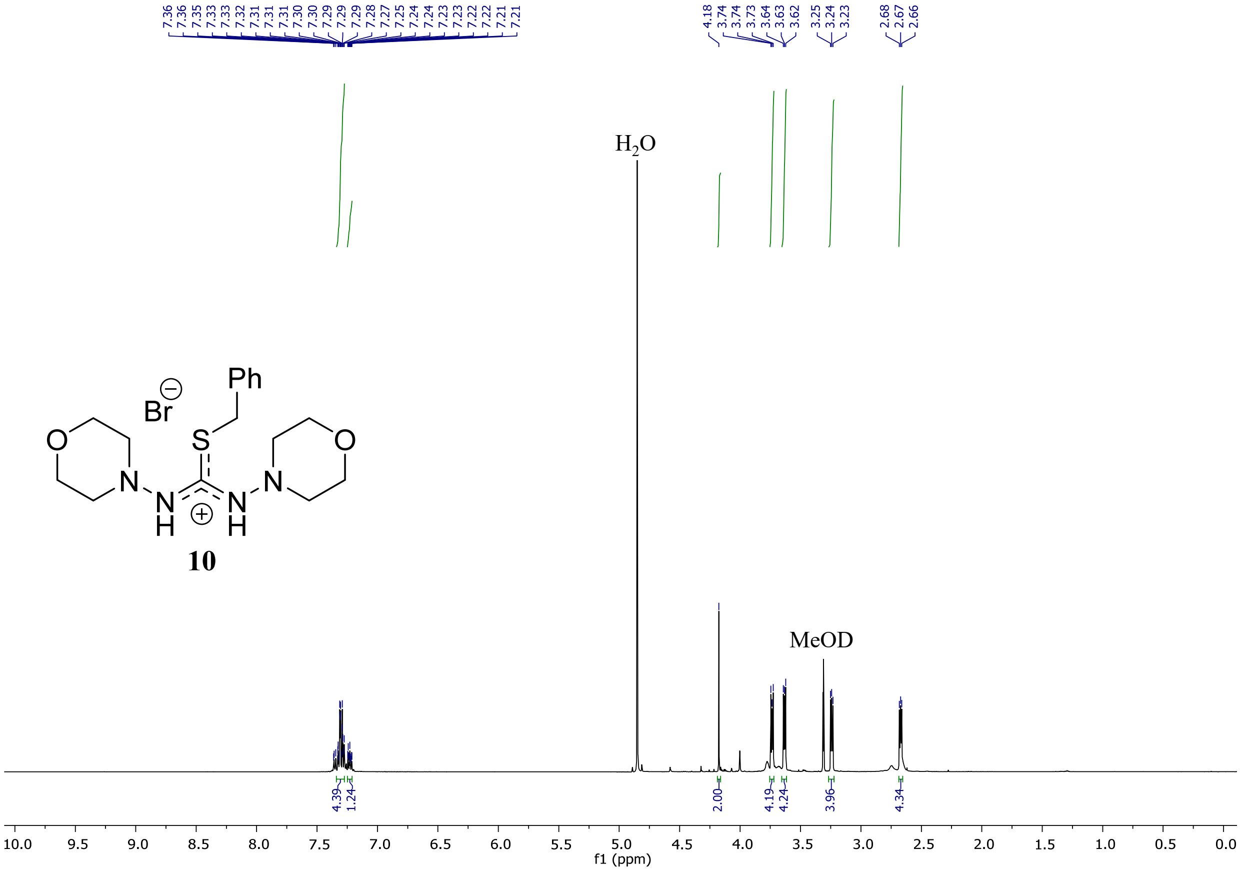

Figure S19. ^{1}H Spectrum of **10** in MeOD (500 MHz)

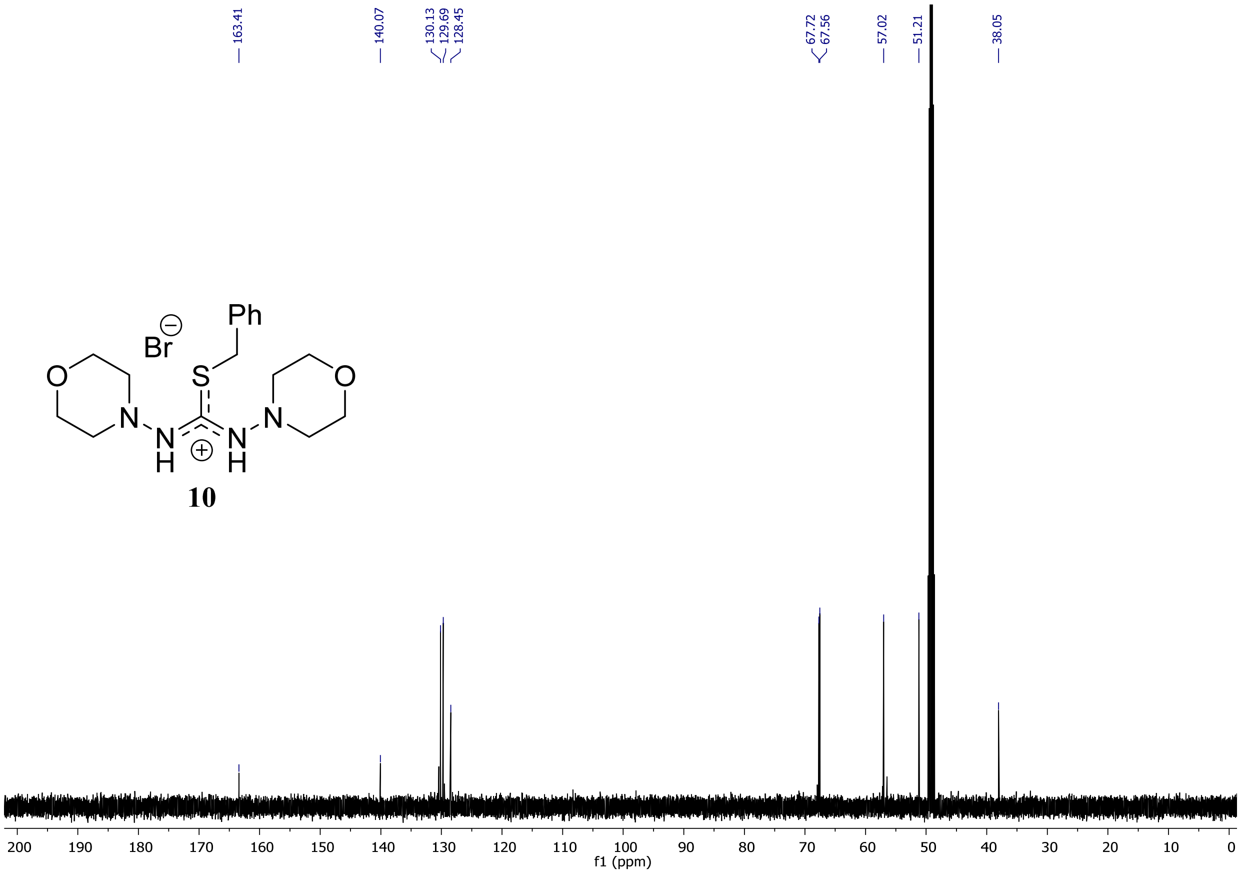

Figure S20. $^{13}C\{^{1}H\}$ Spectrum of **10** in MeOD (125 MHz)

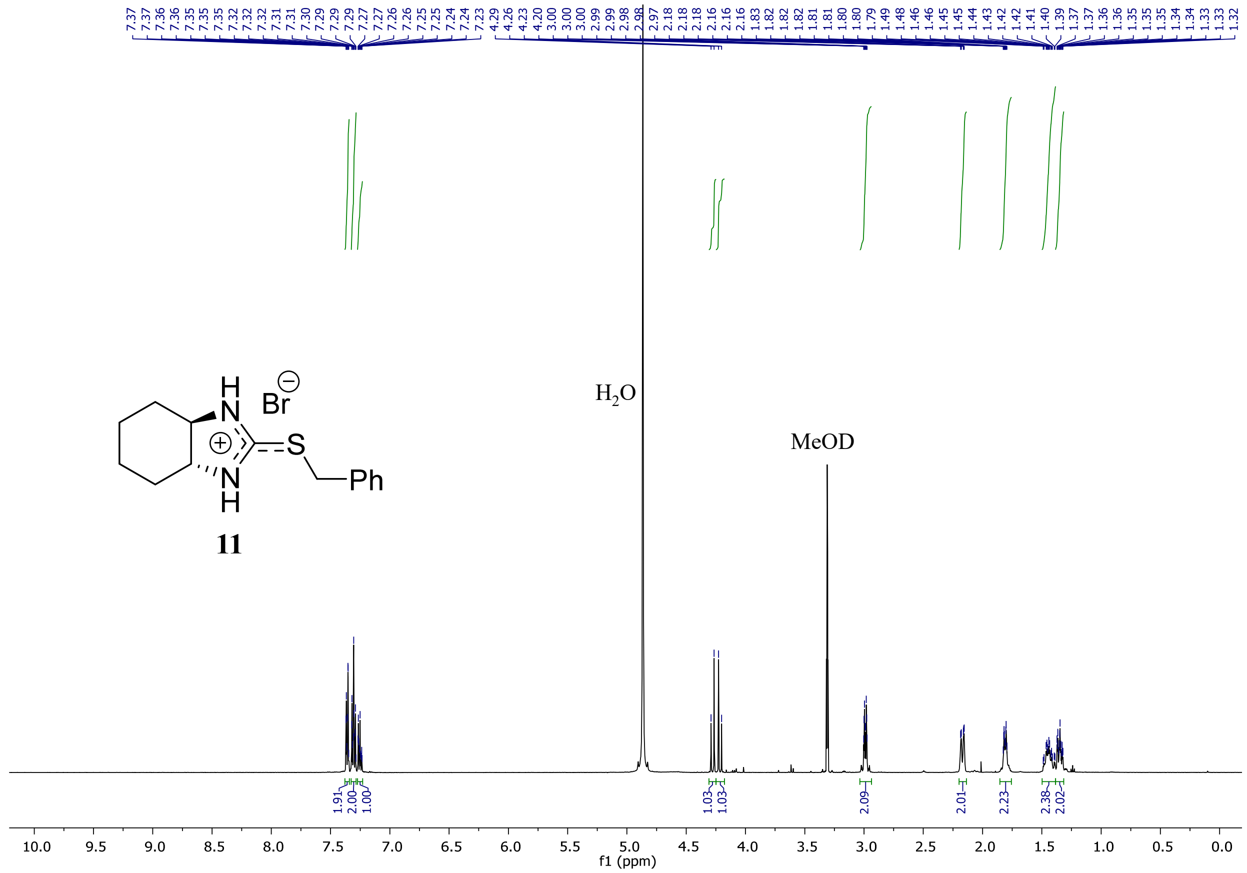

Figure S21. ^{1}H Spectrum of **11** in MeOD (500 MHz)

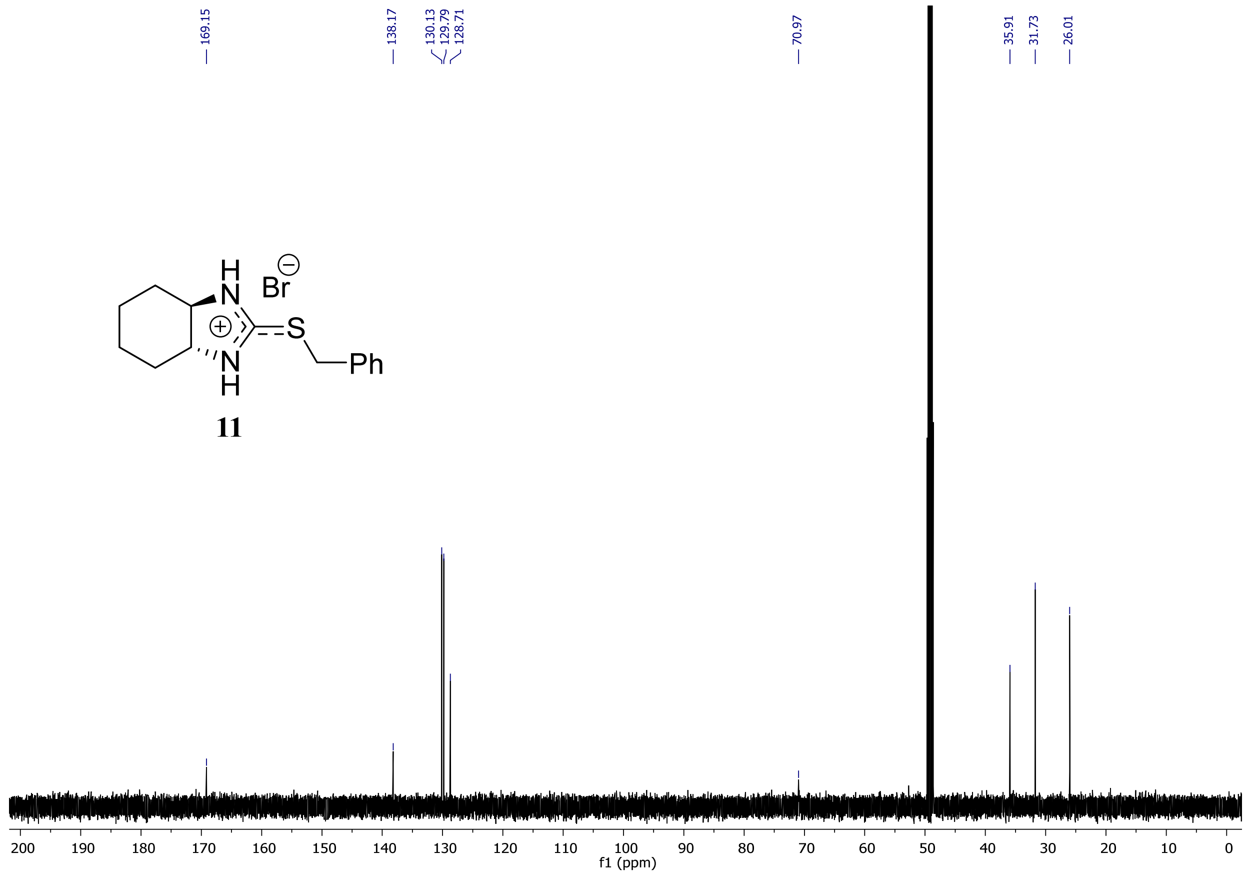

Figure S22. $^{13}C\{^{1}H\}$ Spectrum of **11** in MeOD (125 MHz)

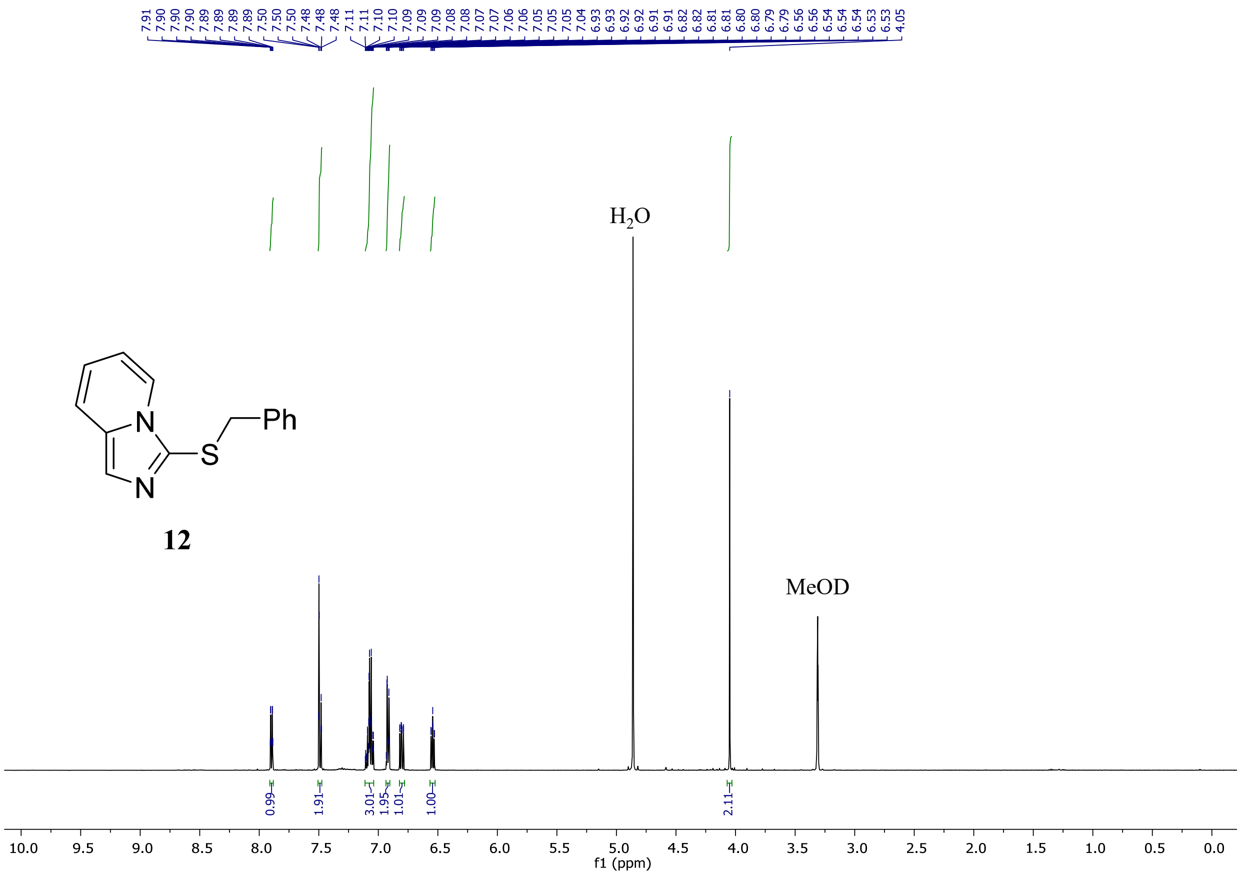

Figure S23. [1]H Spectrum of **12** in MeOD (500 MHz)

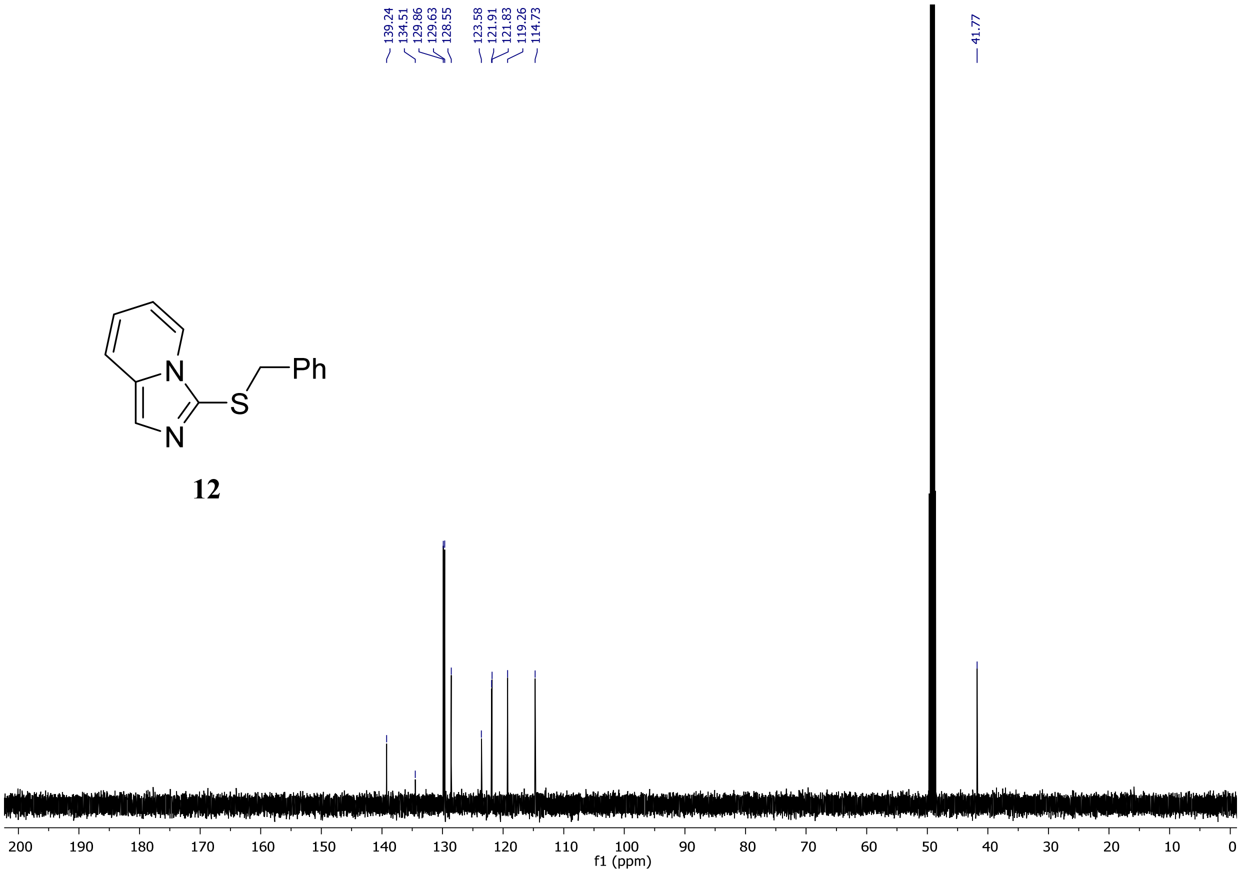

Figure S24. $^{13}C\{^1H\}$ Spectrum of **12** in MeOD (125 MHz)

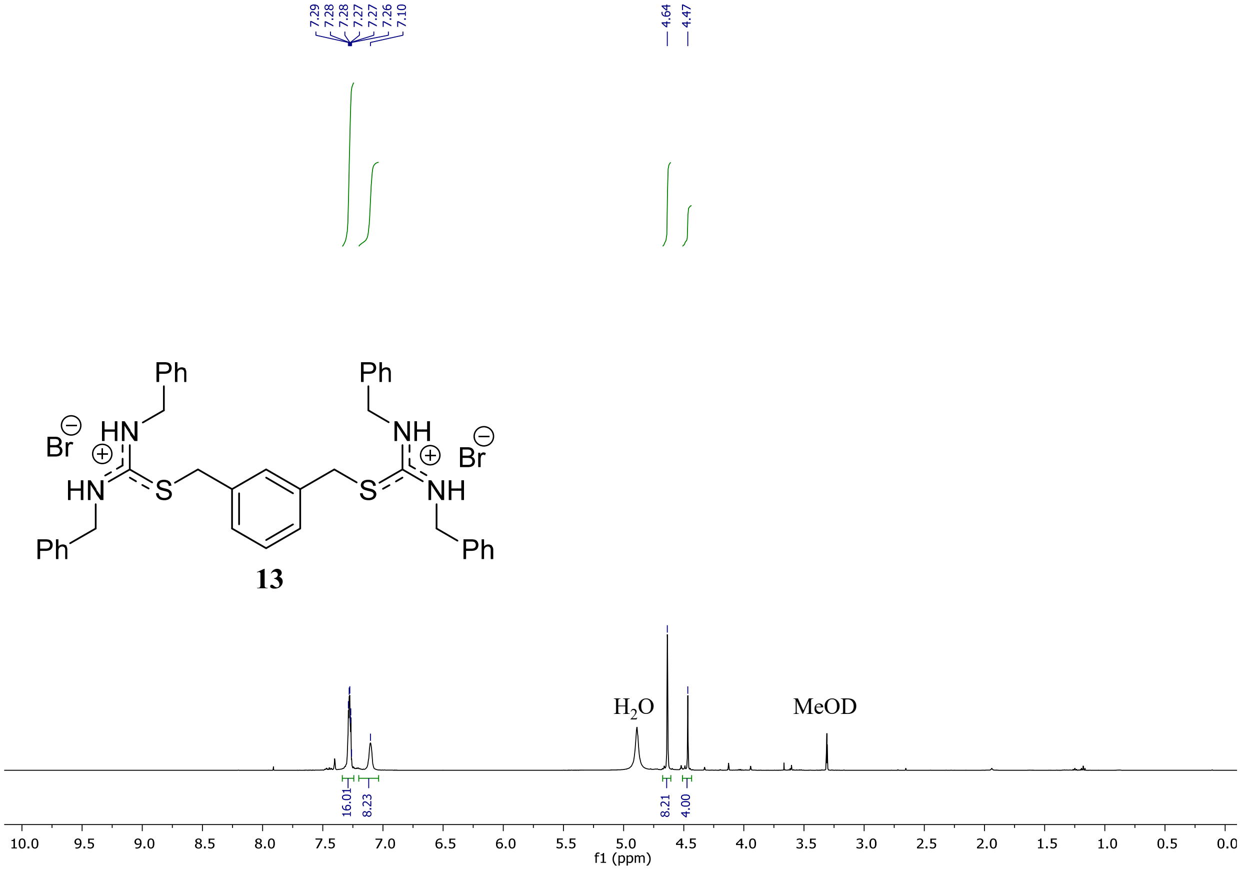

Figure S25. ^{1}H Spectrum of **13** in MeOD (500 MHz)

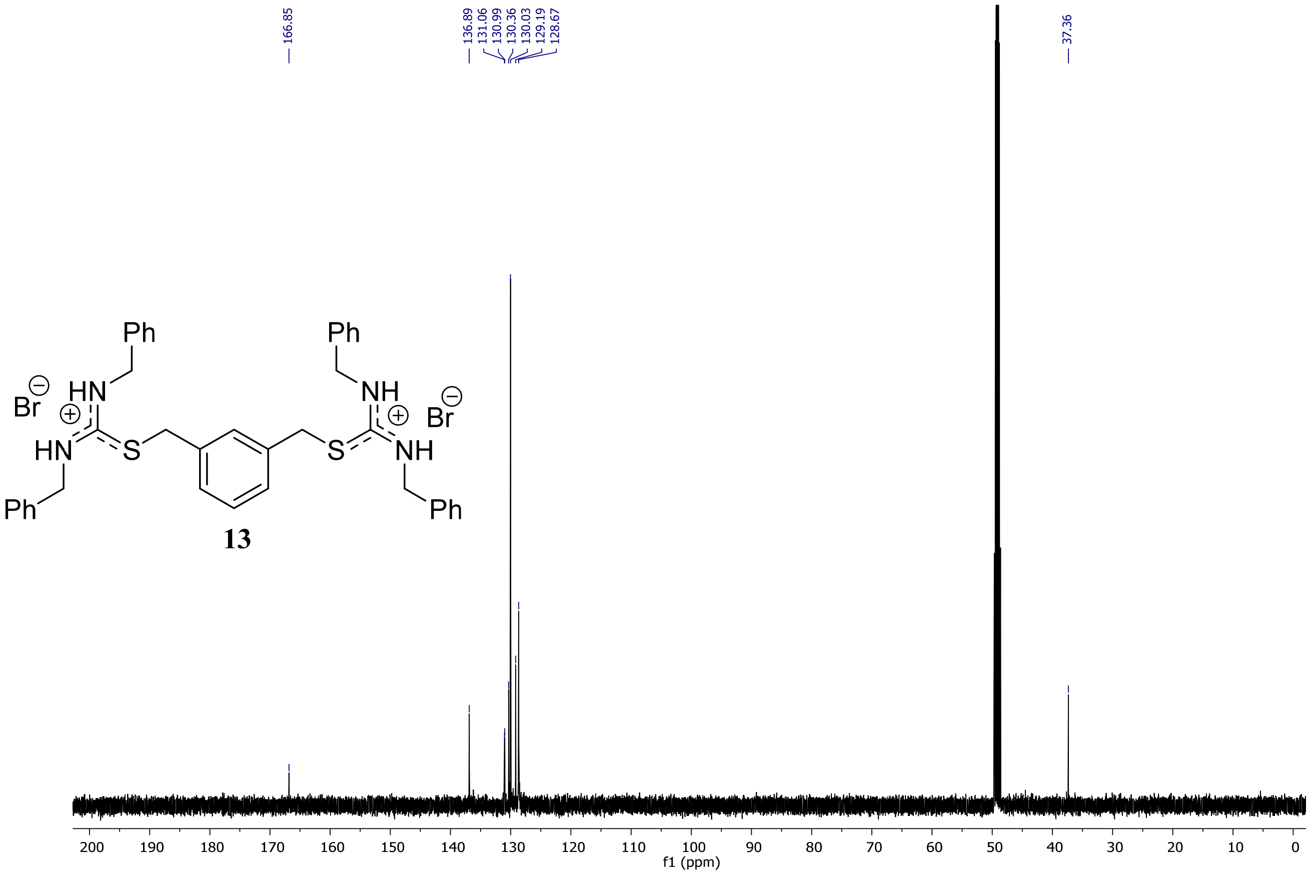

Figure S26. ^{13}C{^{1}H} Spectrum of **13** in MeOD (125 MHz)

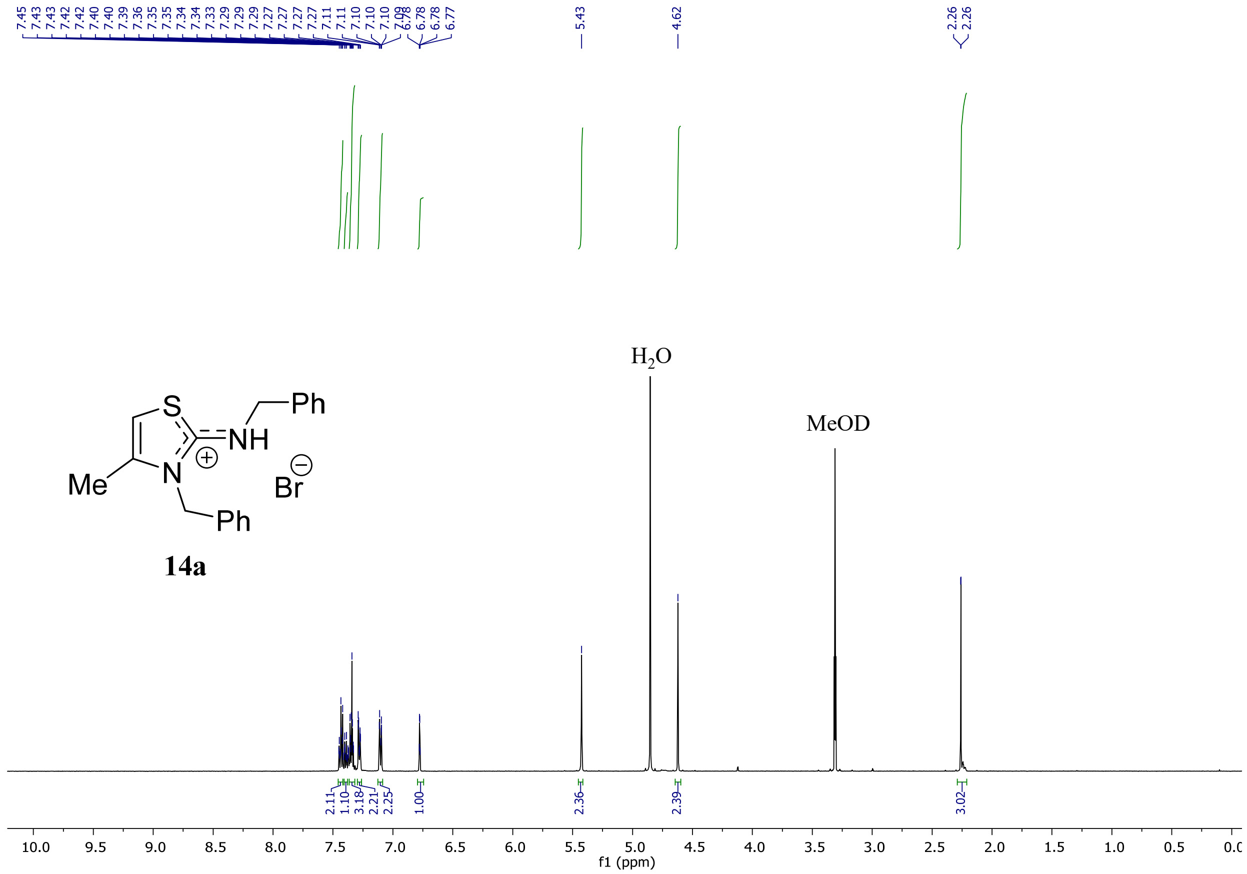

Figure S27. ^{1}H Spectrum of **14a** in MeOD (500 MHz)

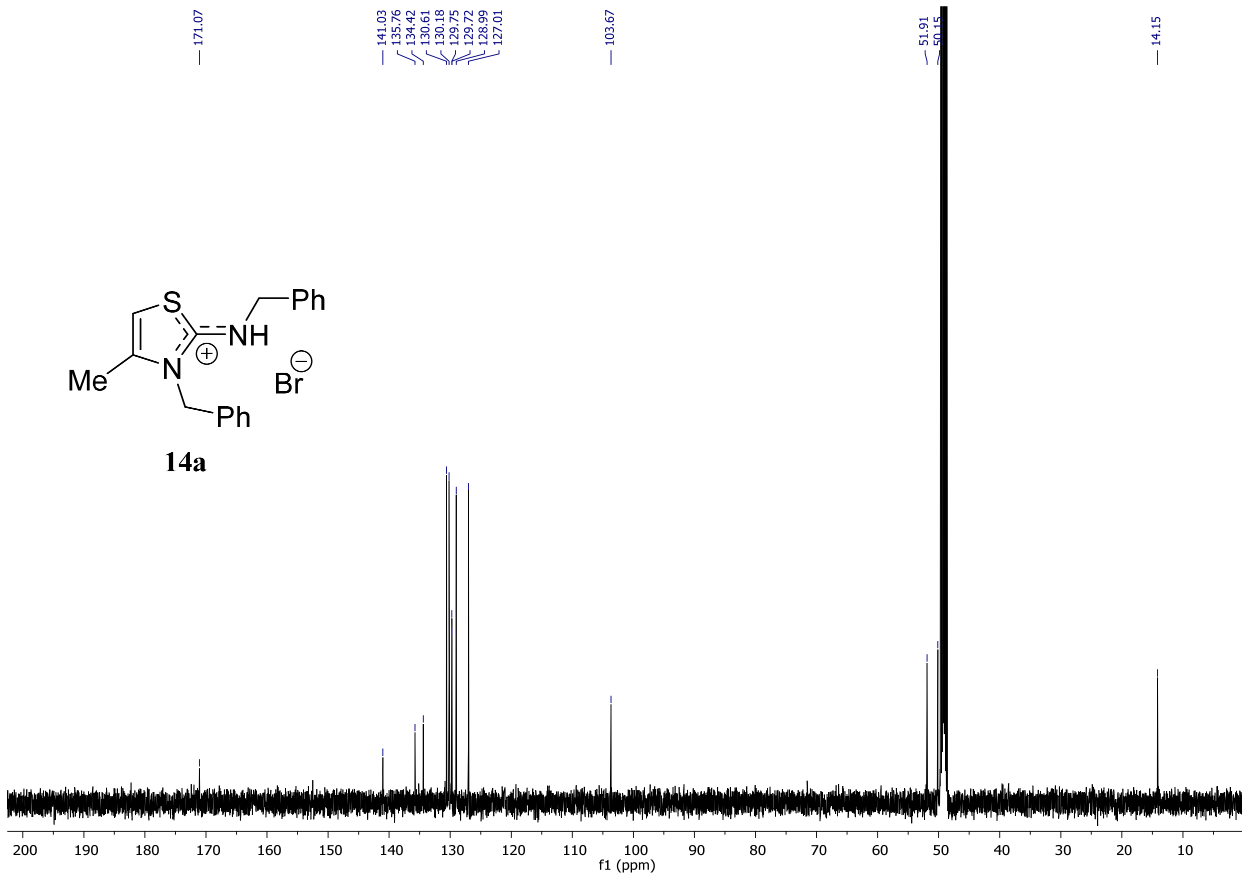

Figure S28. ^{13}C{^{1}H} Spectrum of **14a** in MeOD (125 MHz)

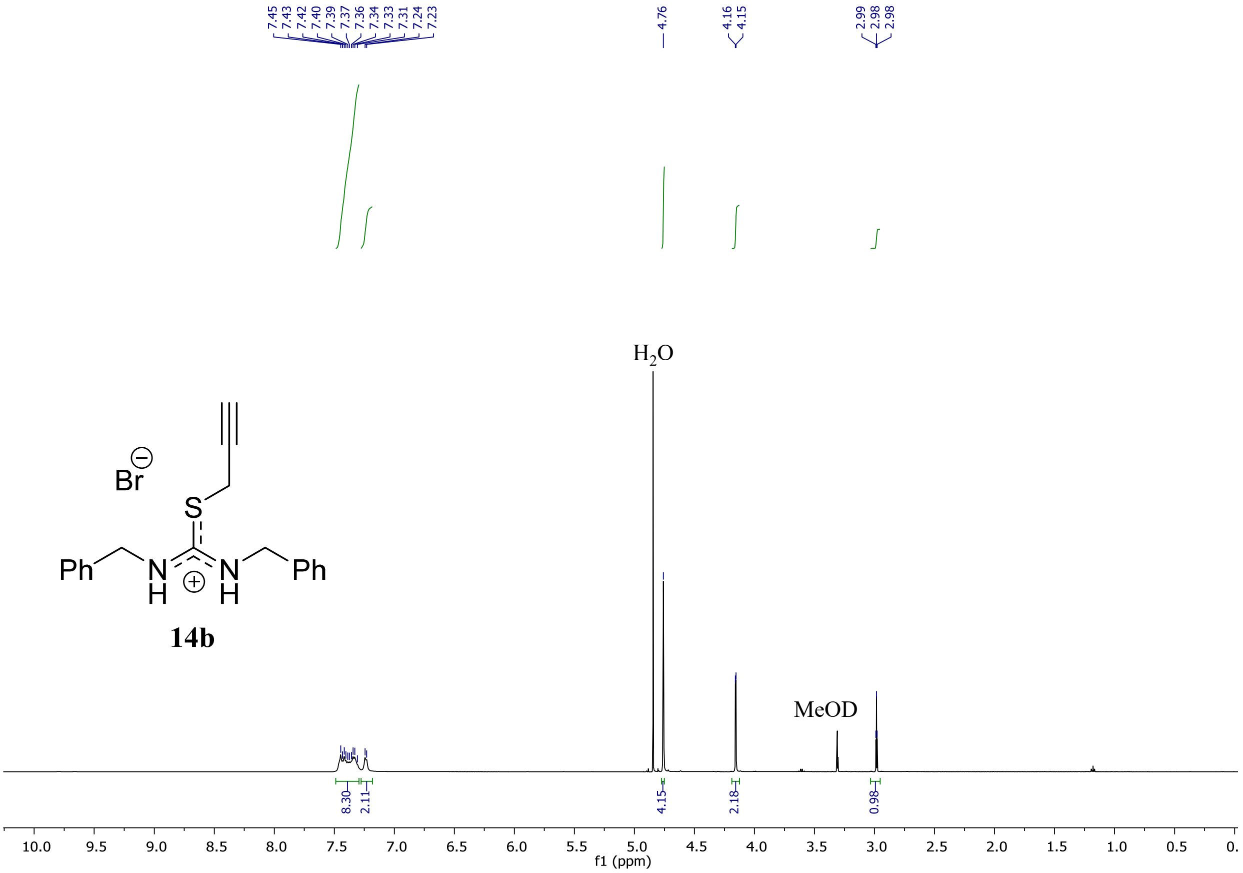

Figure S29. ^{1}H Spectrum of **14b** in MeOD (500 MHz)

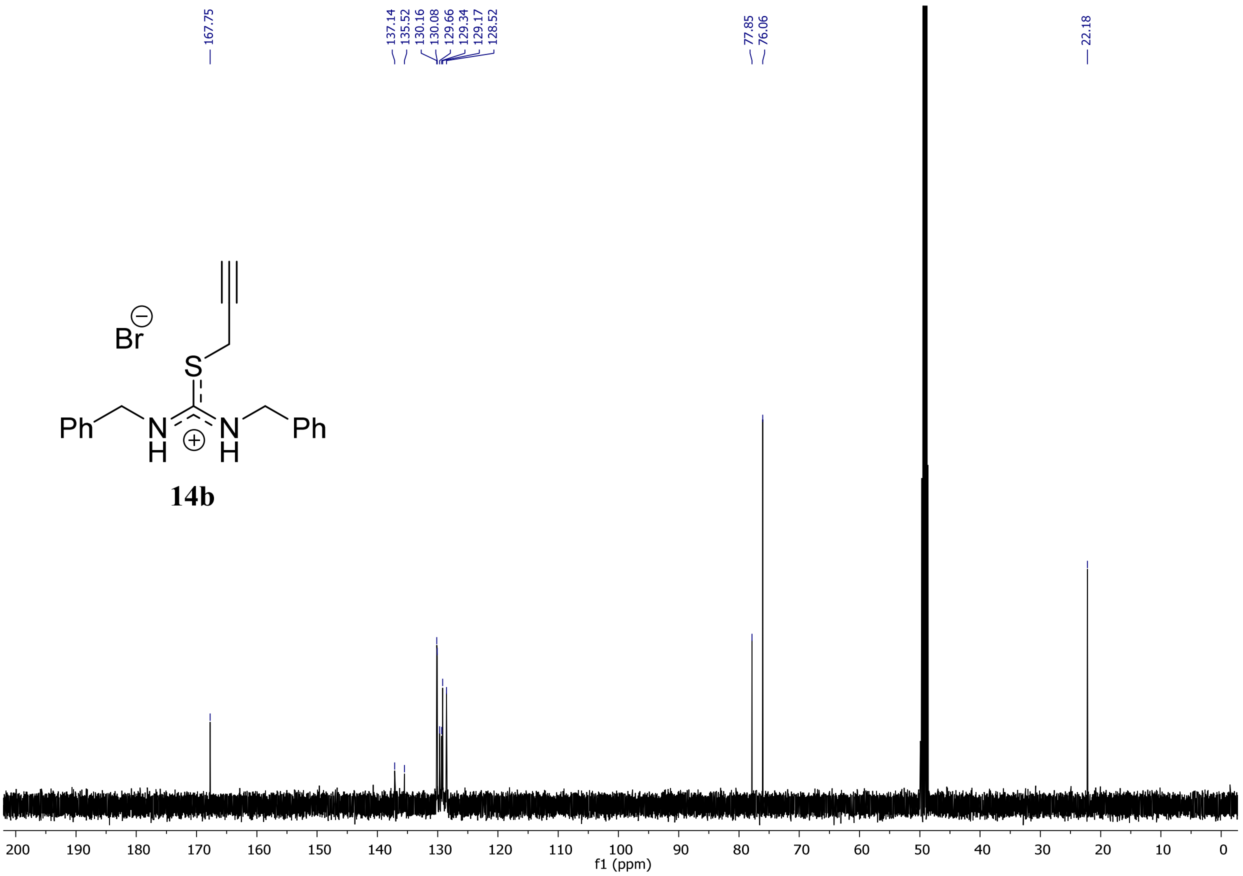

Figure S30. $^{13}C\{^{1}H\}$ Spectrum of **14b** in MeOD (125 MHz)

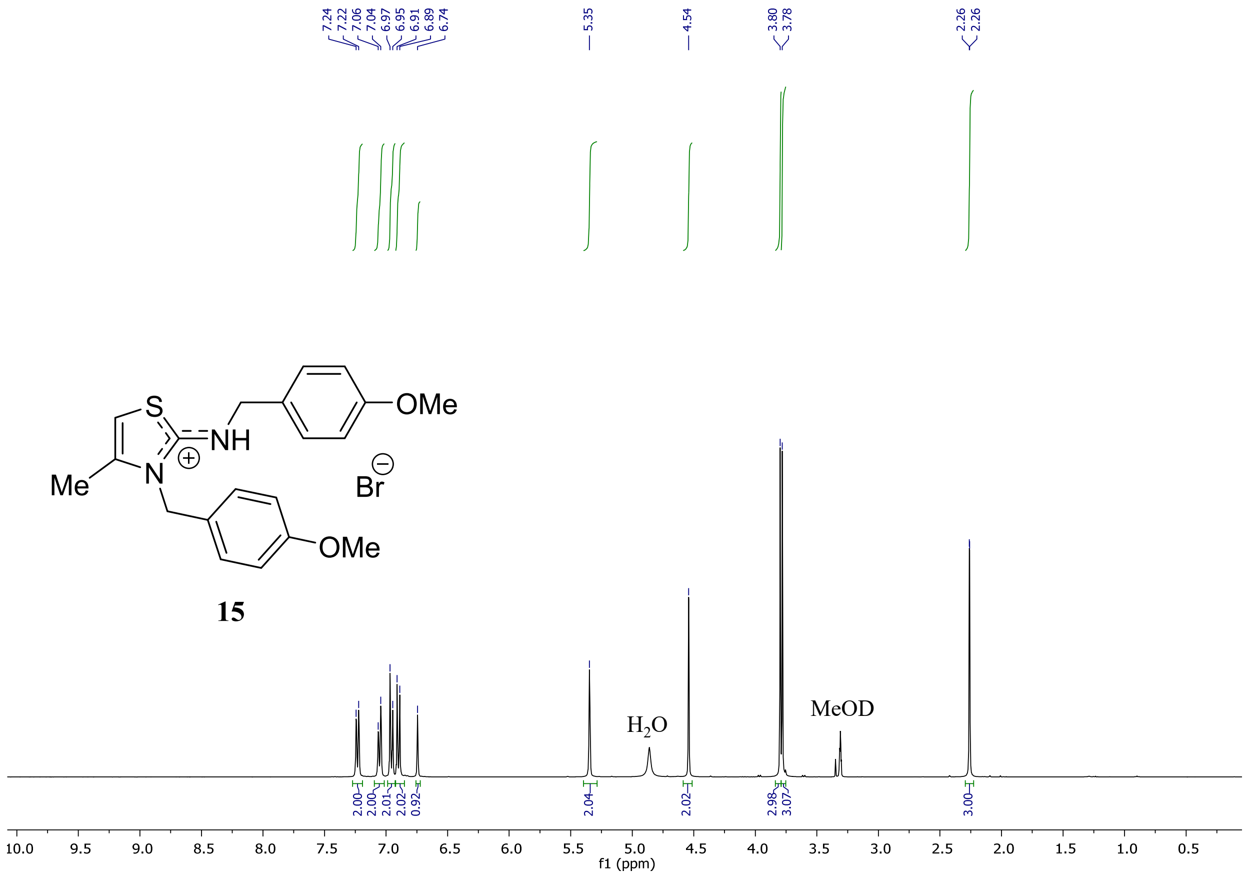

Figure S31. ^{1}H Spectrum of **15** in MeOD (400 MHz)

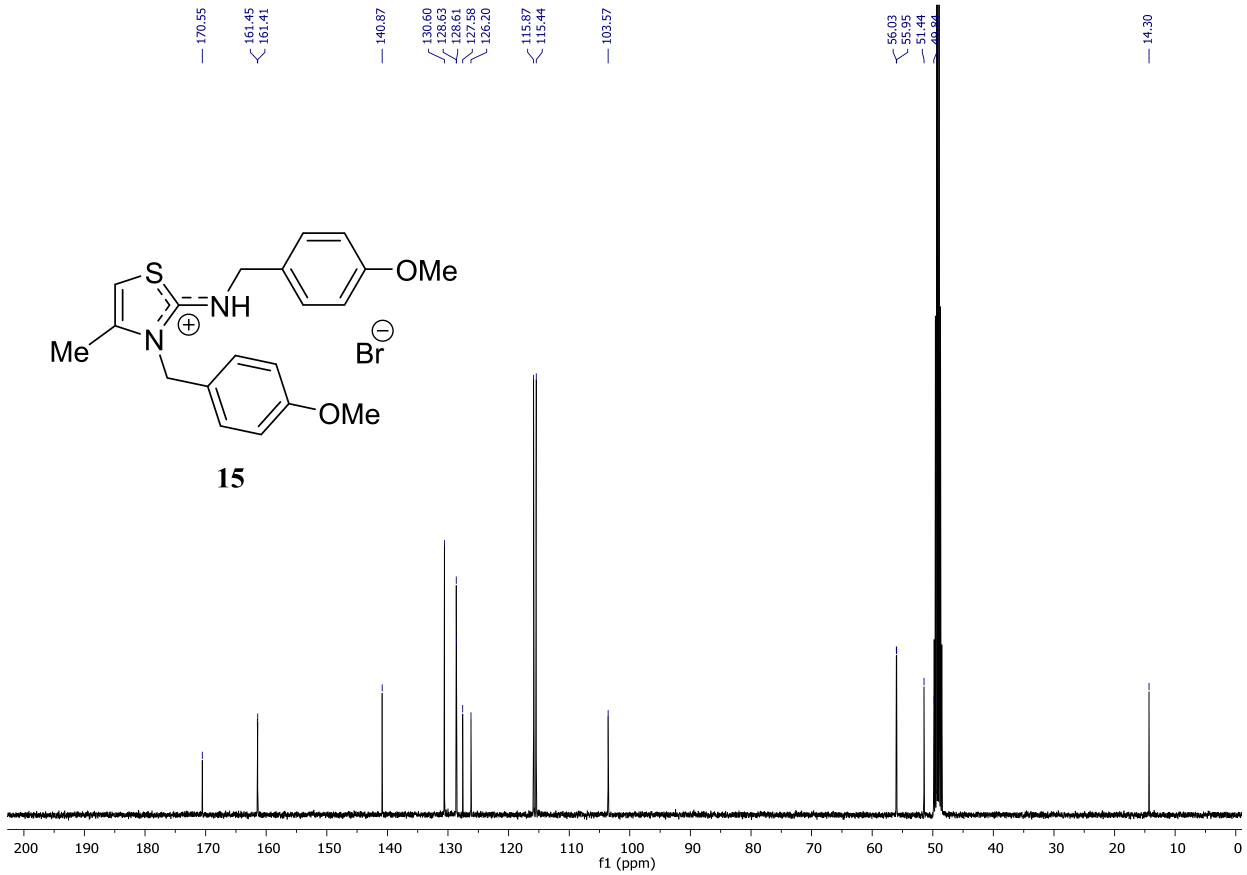

Figure S32. $^{13}C\{^{1}H\}$ Spectrum of **15** in MeOD (100 MHz)

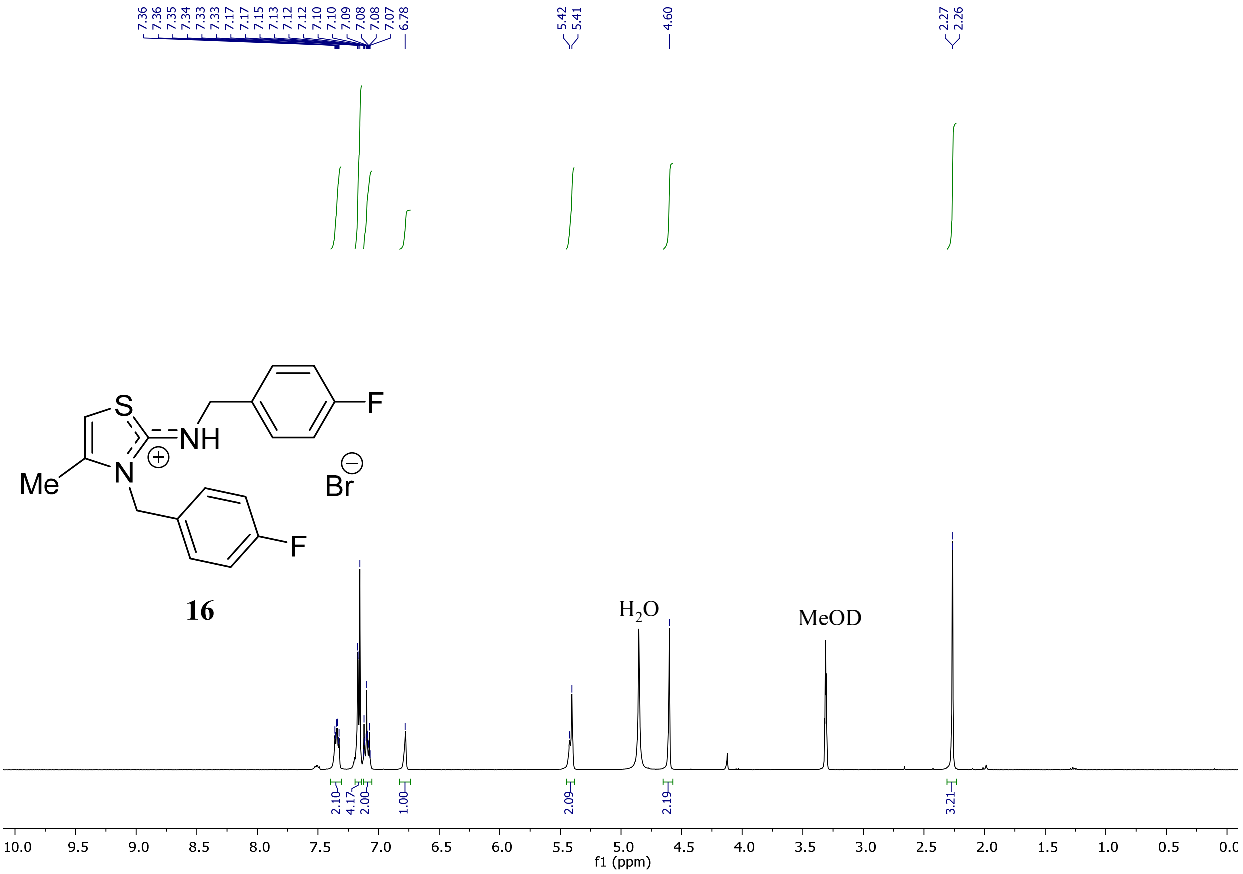

Figure S33. ^{1}H Spectrum of **16** in MeOD (400 MHz)

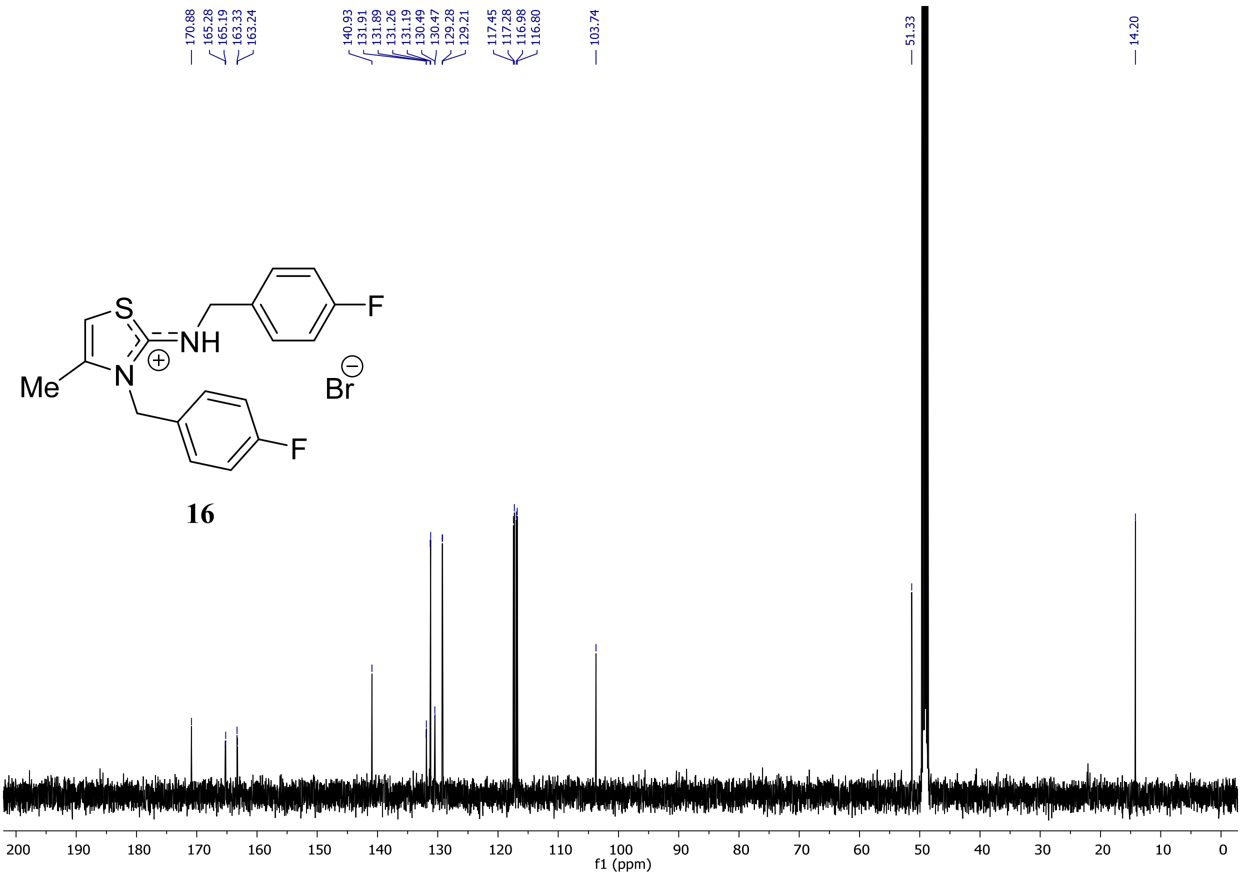

Figure S34. $^{13}C\{^{1}H\}$ Spectrum of **16** in MeOD (125 MHz)

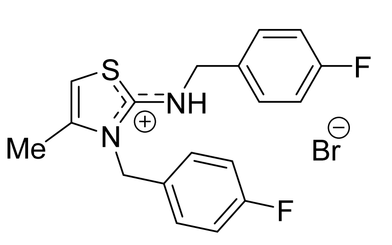

16

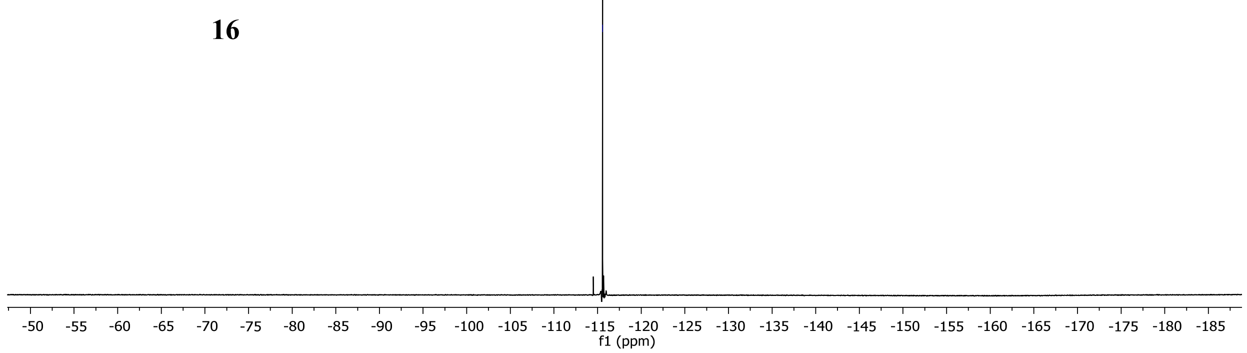

Figure S35. ^{19}F Spectrum of **16** in MeOD (376 MHz)

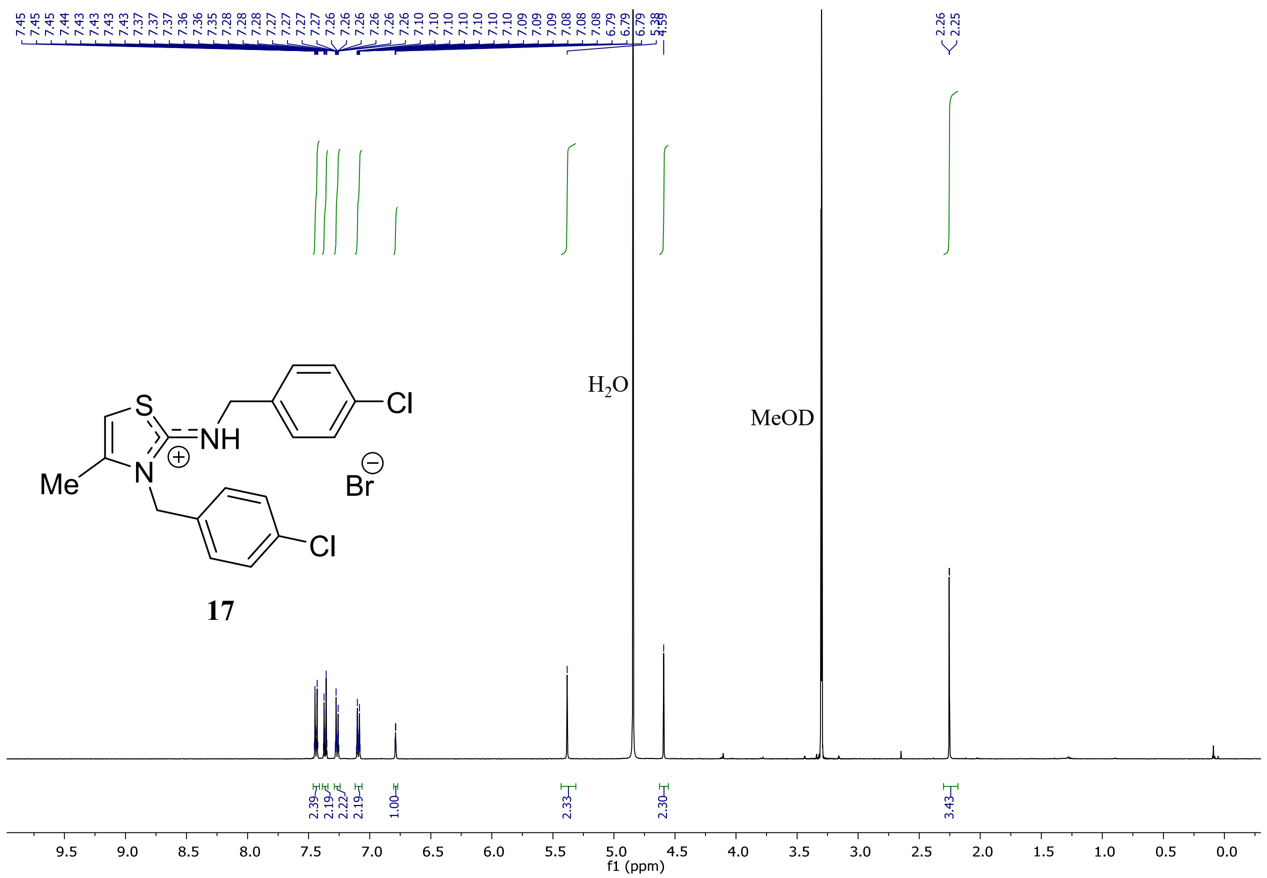

Figure S36. ^{1}H Spectrum of **17** in MeOD (500 MHz)

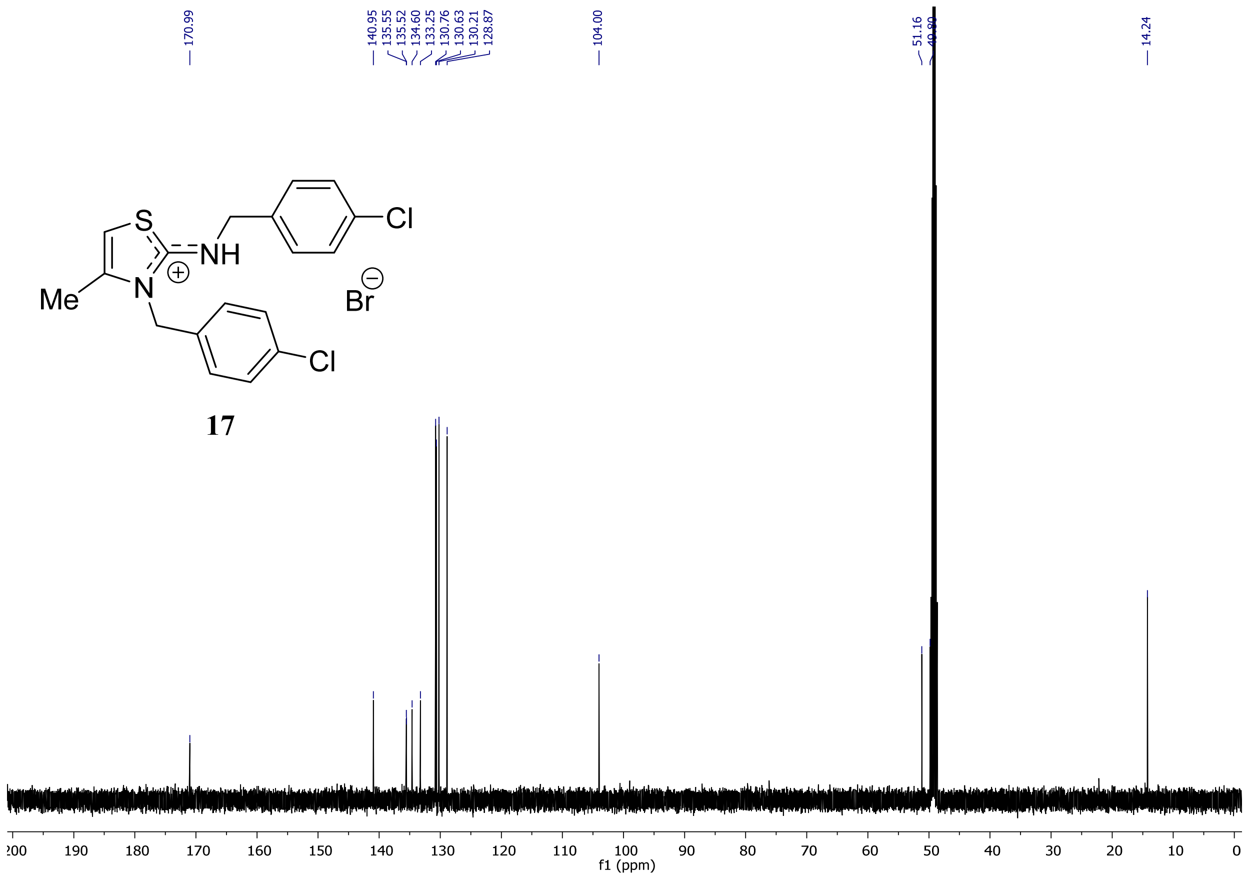

Figure S37. $^{13}C\{^{1}H\}$ Spectrum of **17** in MeOD (125 MHz)

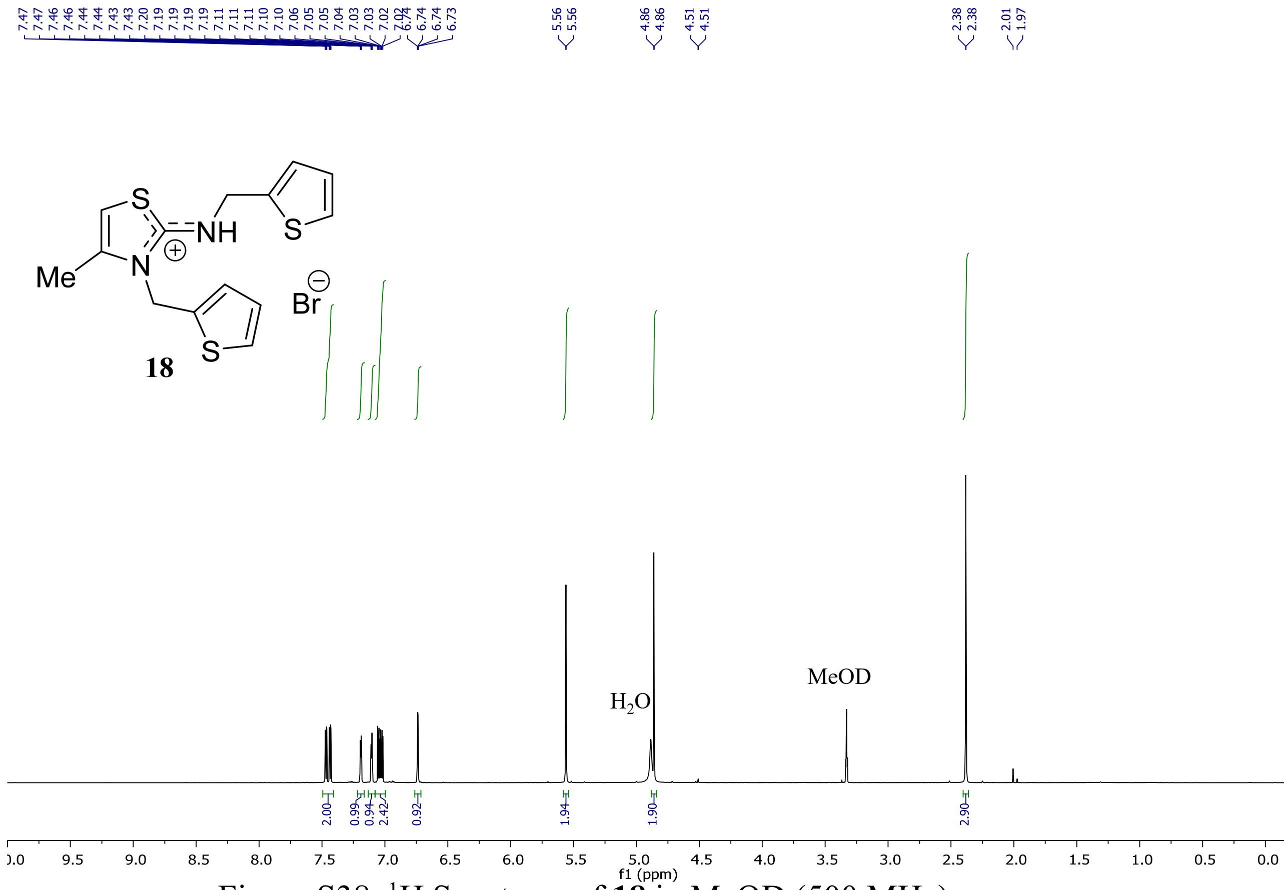

Figure S38. ^{1}H Spectrum of **18** in MeOD (500 MHz)

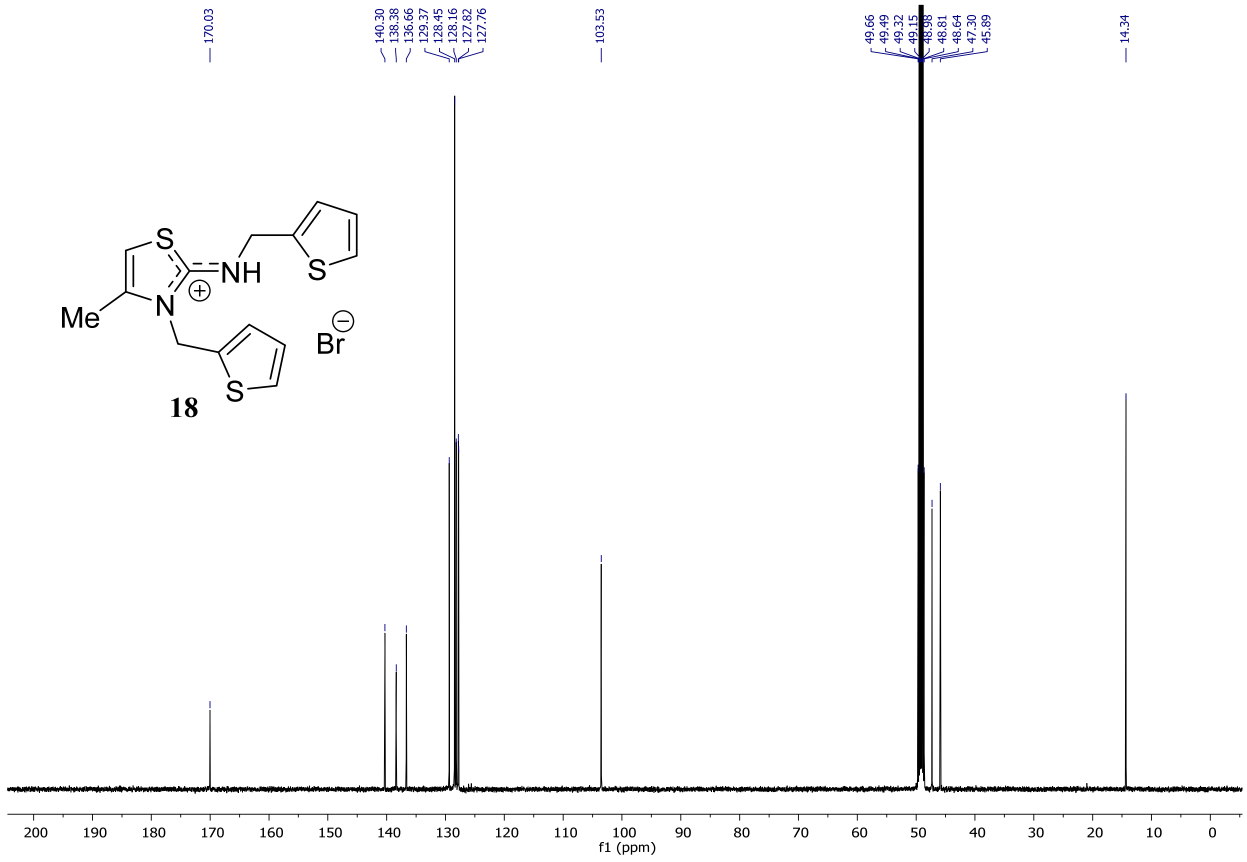

Figure S39. $^{13}C\{^{1}H\}$ Spectrum of **18** in MeOD (125 MHz)

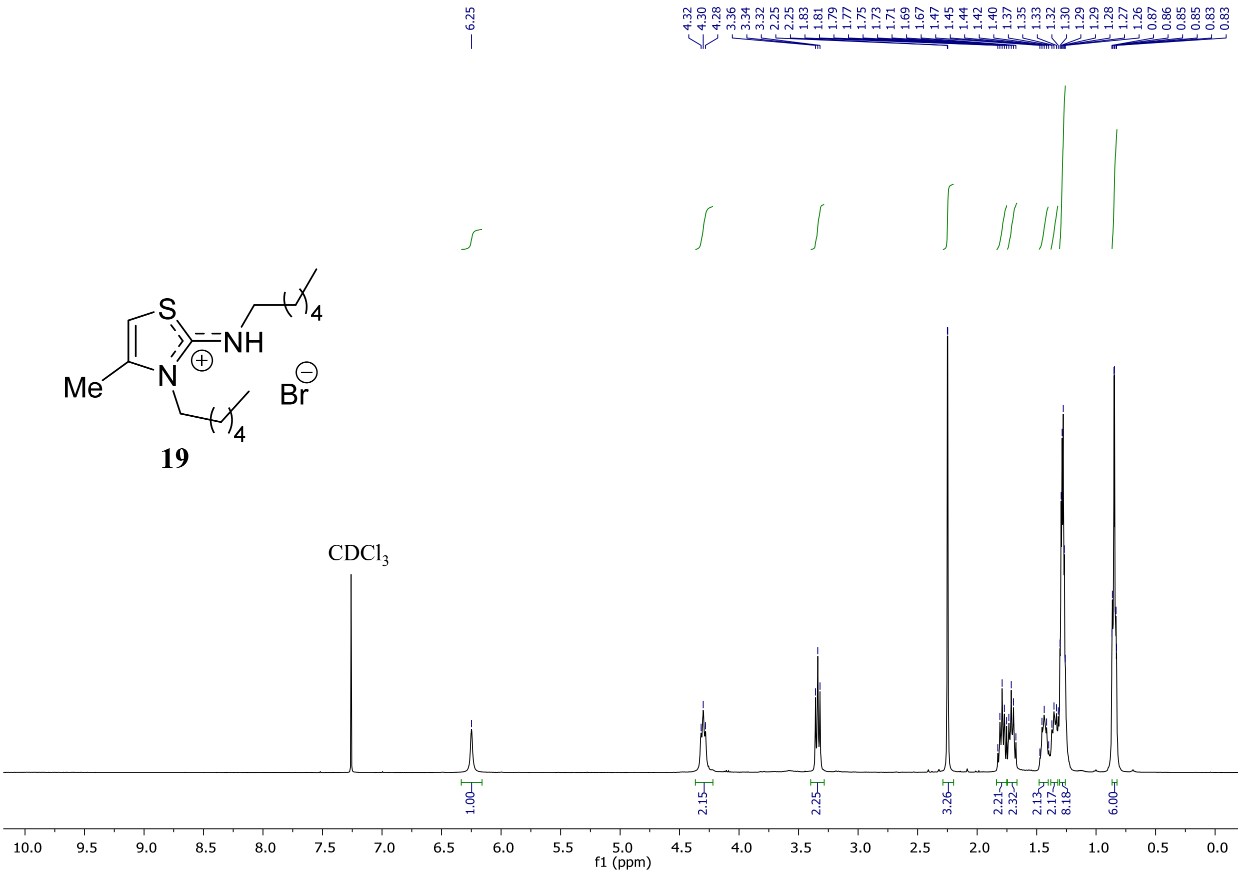

Figure S40. [1]H Spectrum of **19** in $CDCl_3$ (400 MHz)

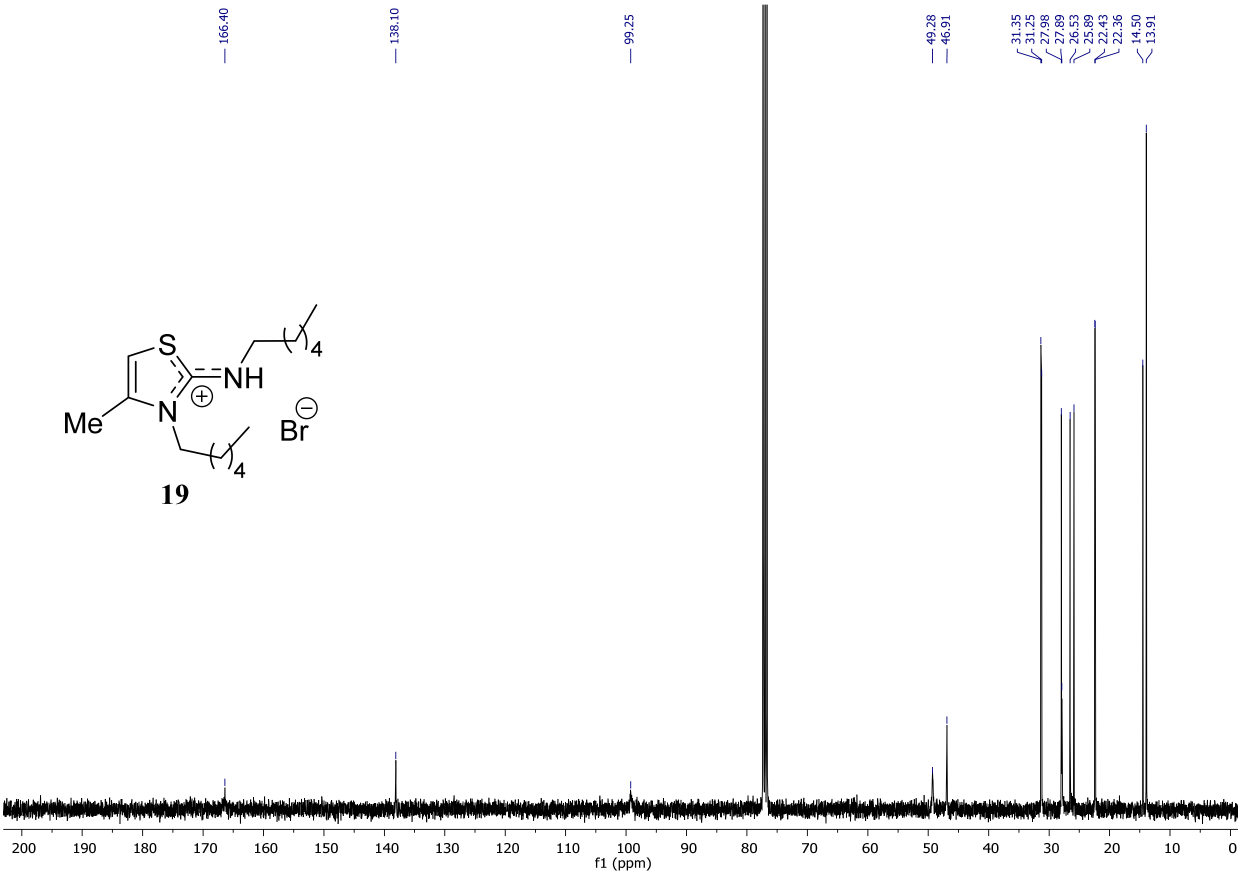

Figure S41. ^{13}C{^{1}H} Spectrum of **19** in $CDCl_3$ (100 MHz)

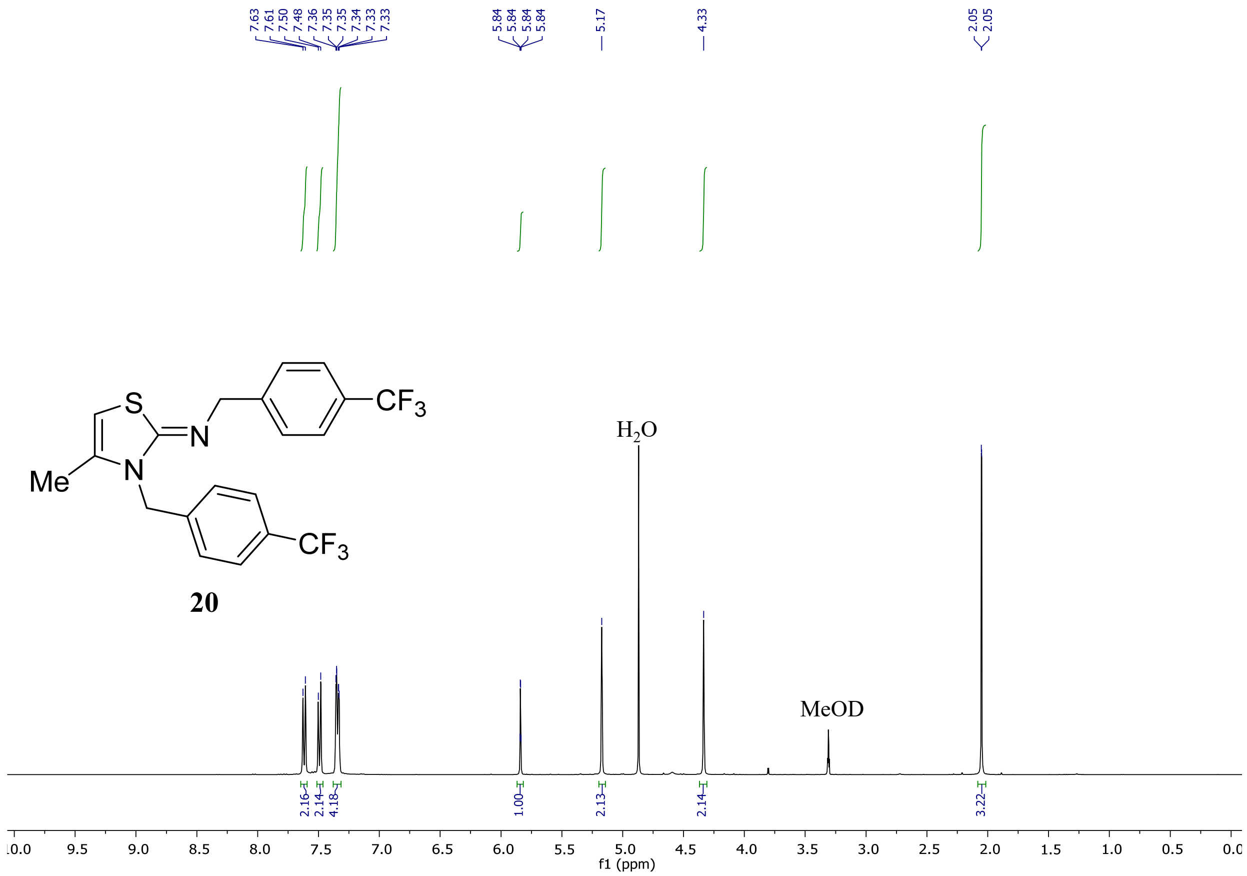

Figure S42. ^{1}H Spectrum of **20** in MeOD (400 MHz)

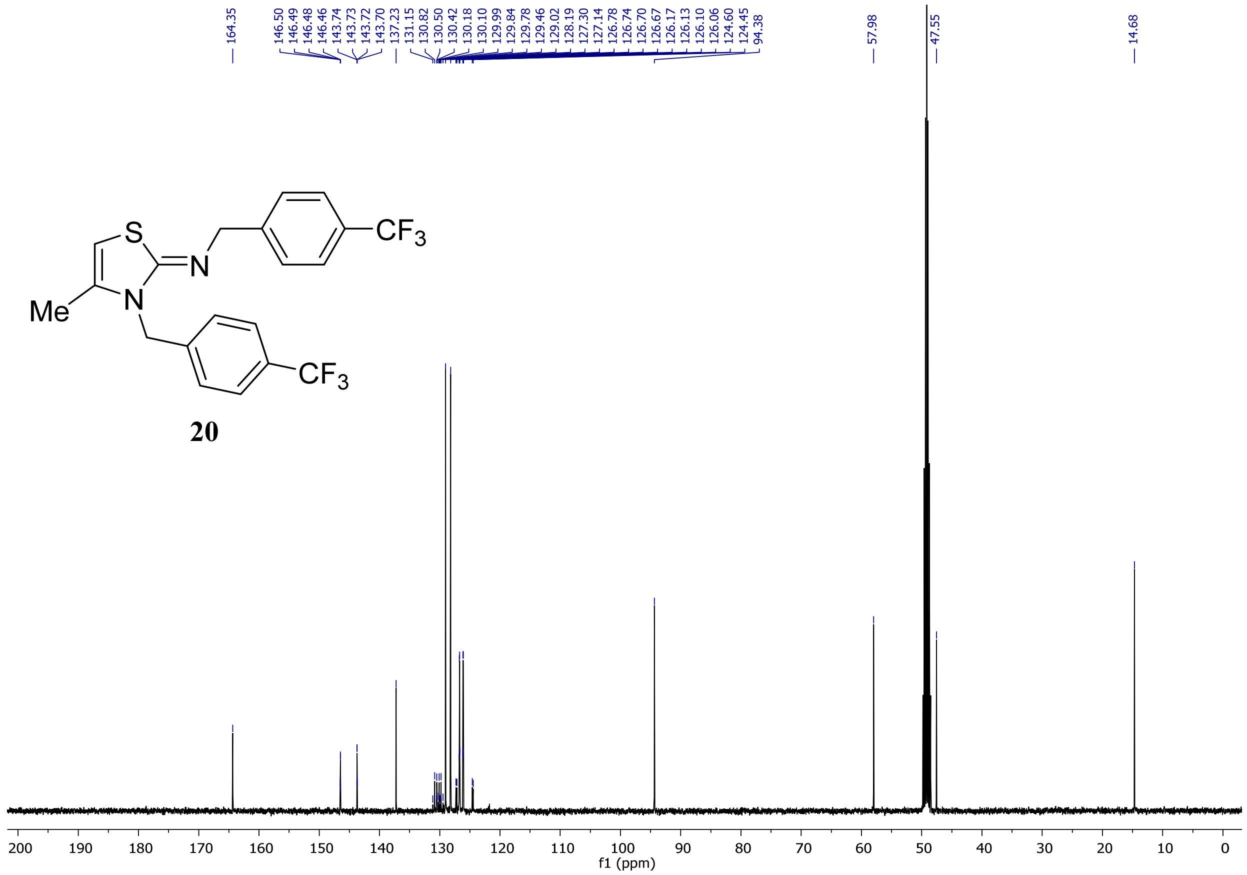

Figure S43. $^{13}C\{^{1}H\}$ Spectrum of **20** in MeOD (100 MHz)

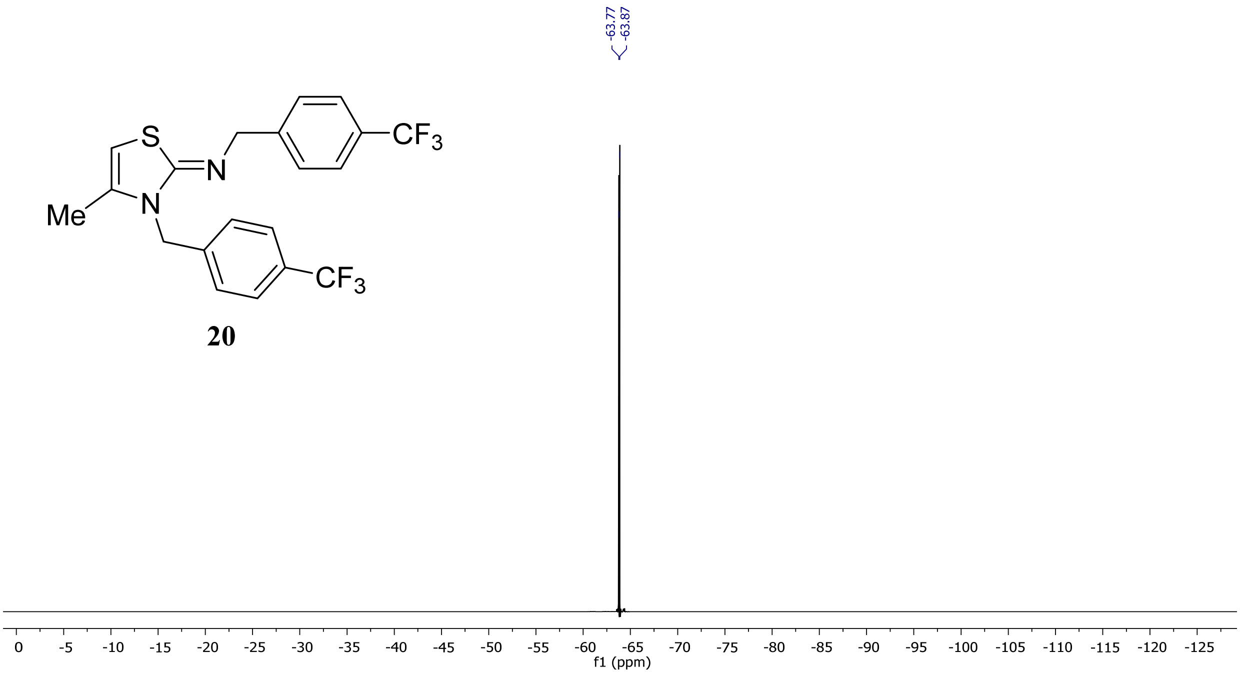

Figure S44. ^{19}F Spectrum of **20** in MeOD (376 MHz)

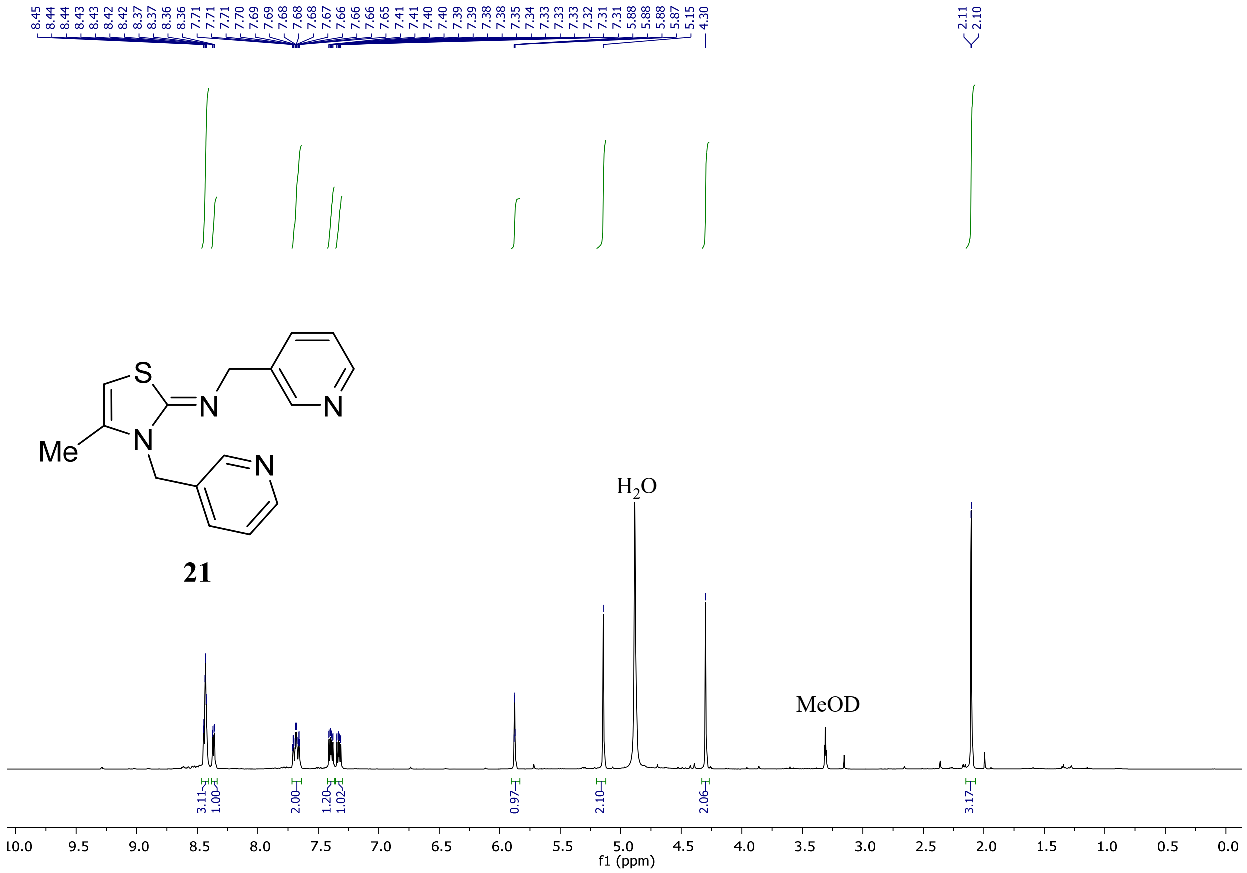

Figure S45. ^{1}H Spectrum of **21** in MeOD (400 MHz)

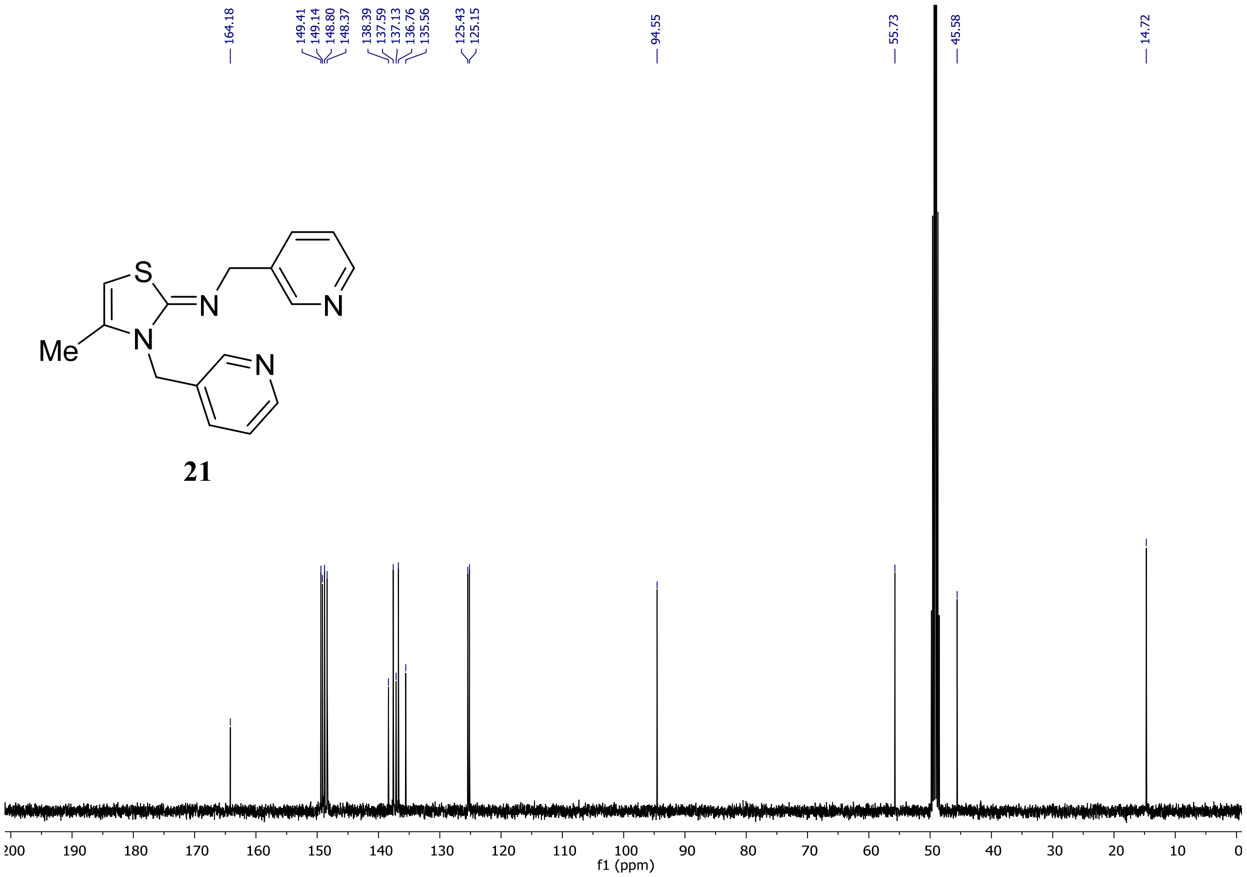

Figure S46. $^{13}C\{^{1}H\}$ Spectrum of **21** in MeOD (100 MHz)

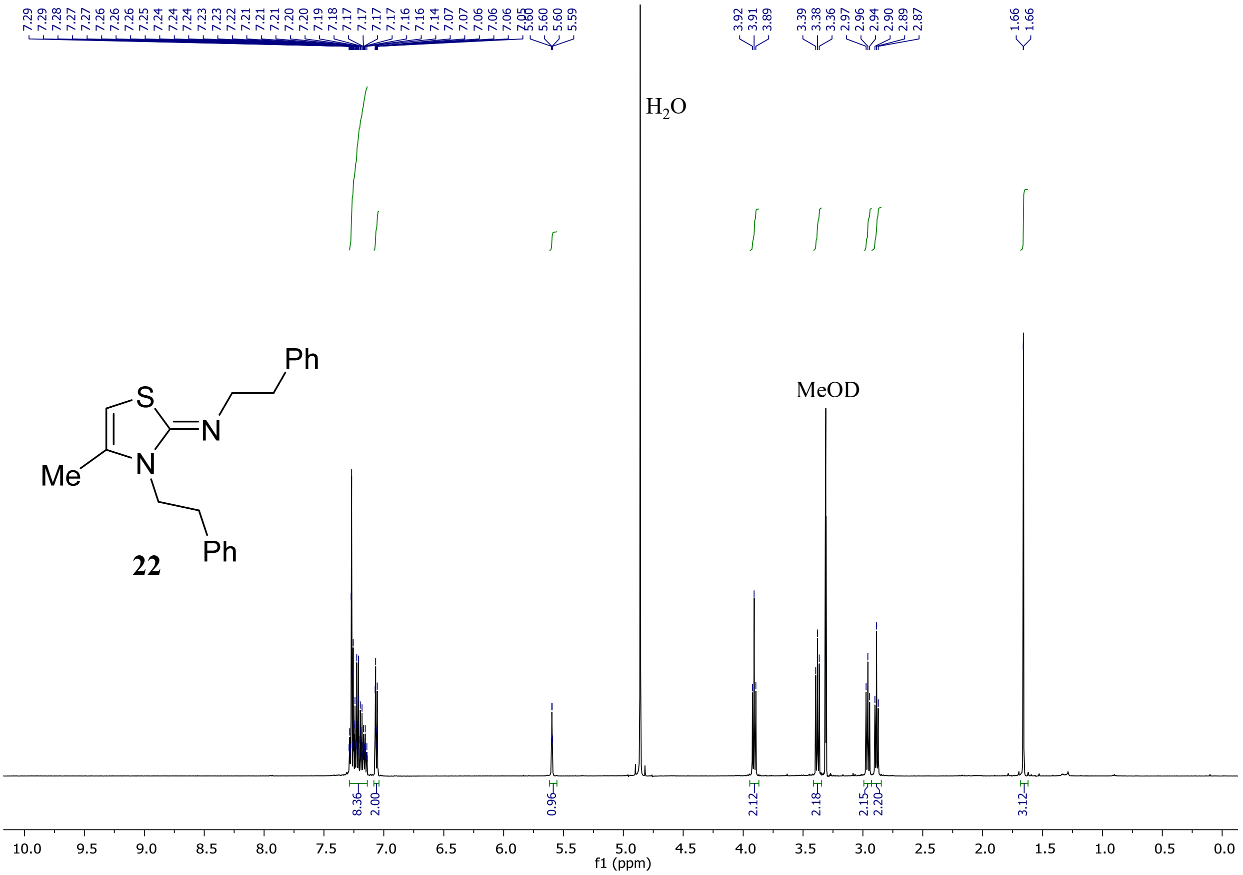

Figure S47. ^{1}H Spectrum of **22** in MeOD (500 MHz)

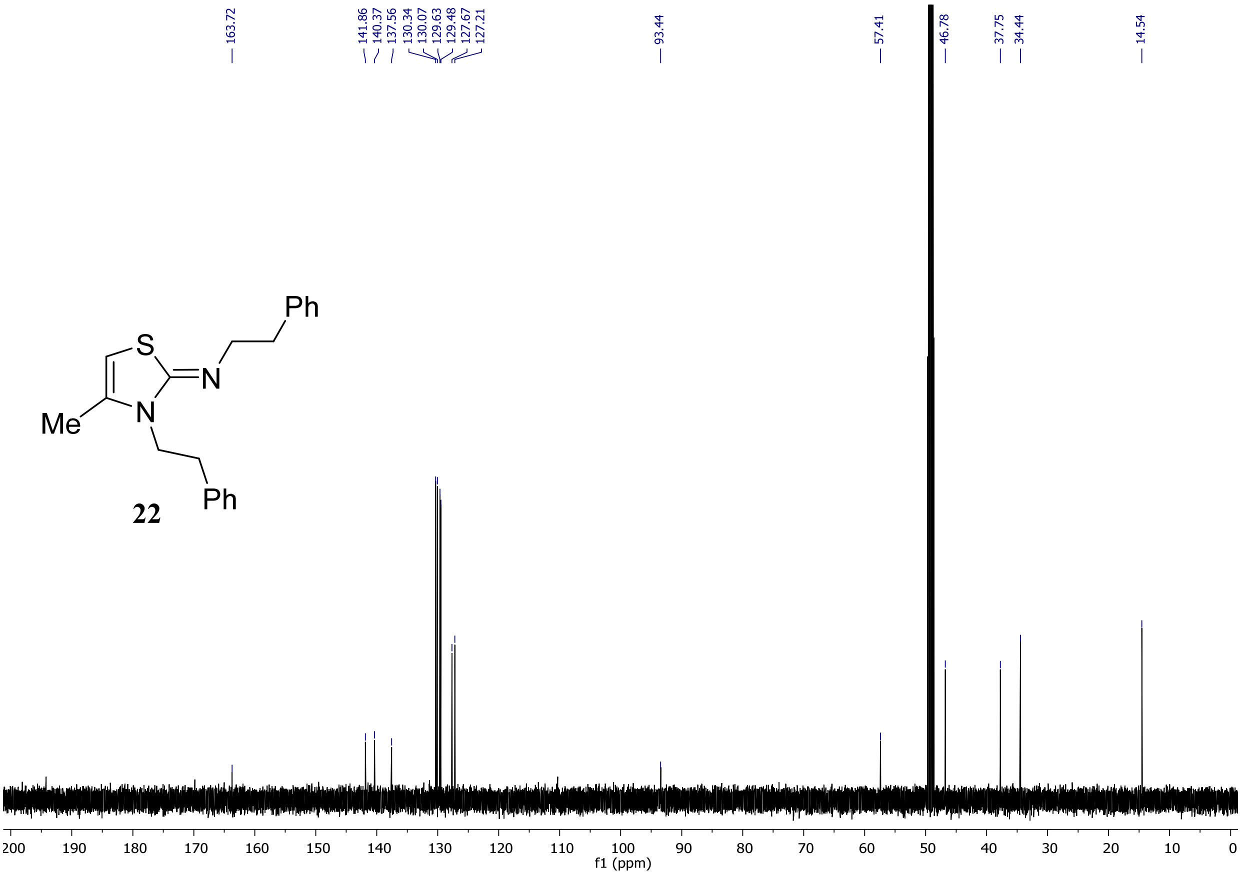

Figure S48. $^{13}C\{^{1}H\}$ Spectrum of **22** in MeOD (125 MHz)

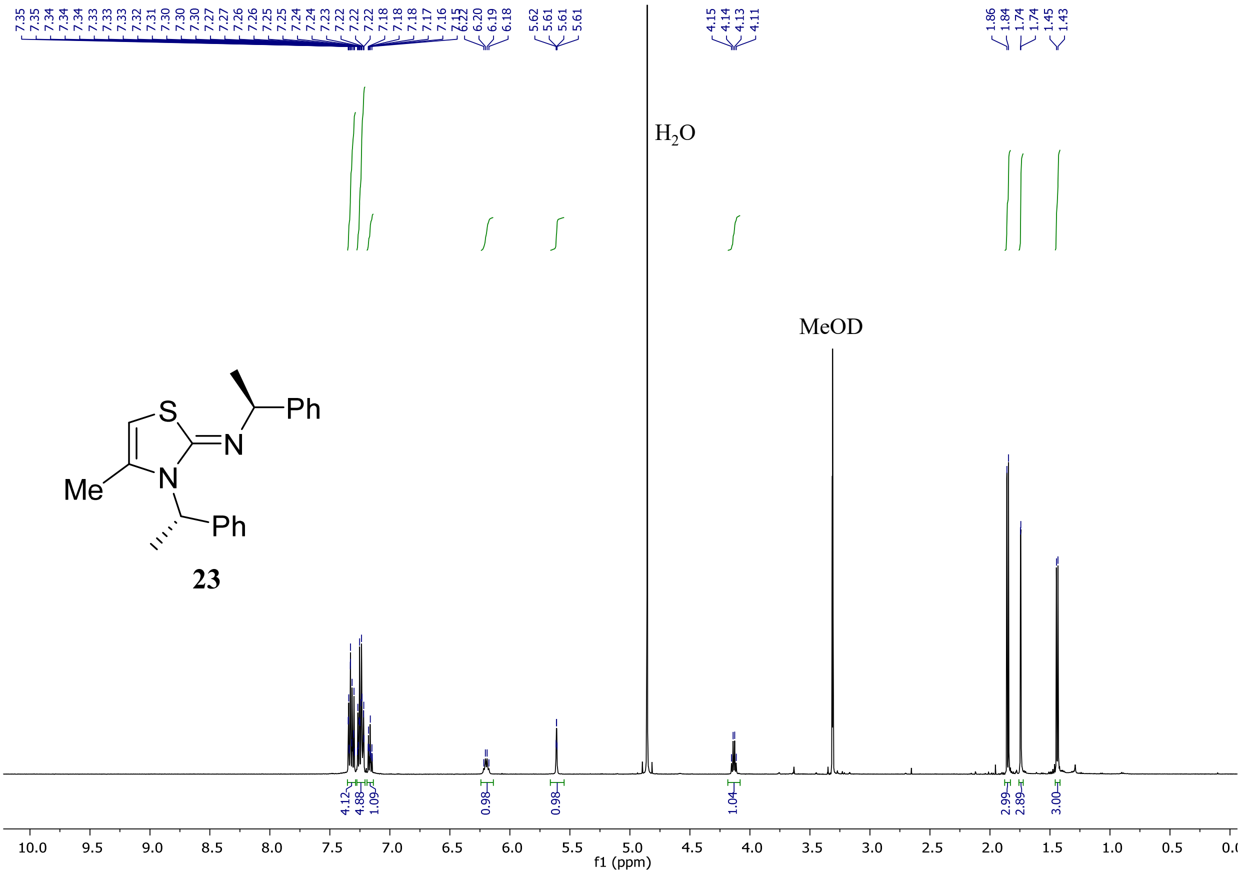

Figure S49. ^{1}H Spectrum of **23** in MeOD (500 MHz)

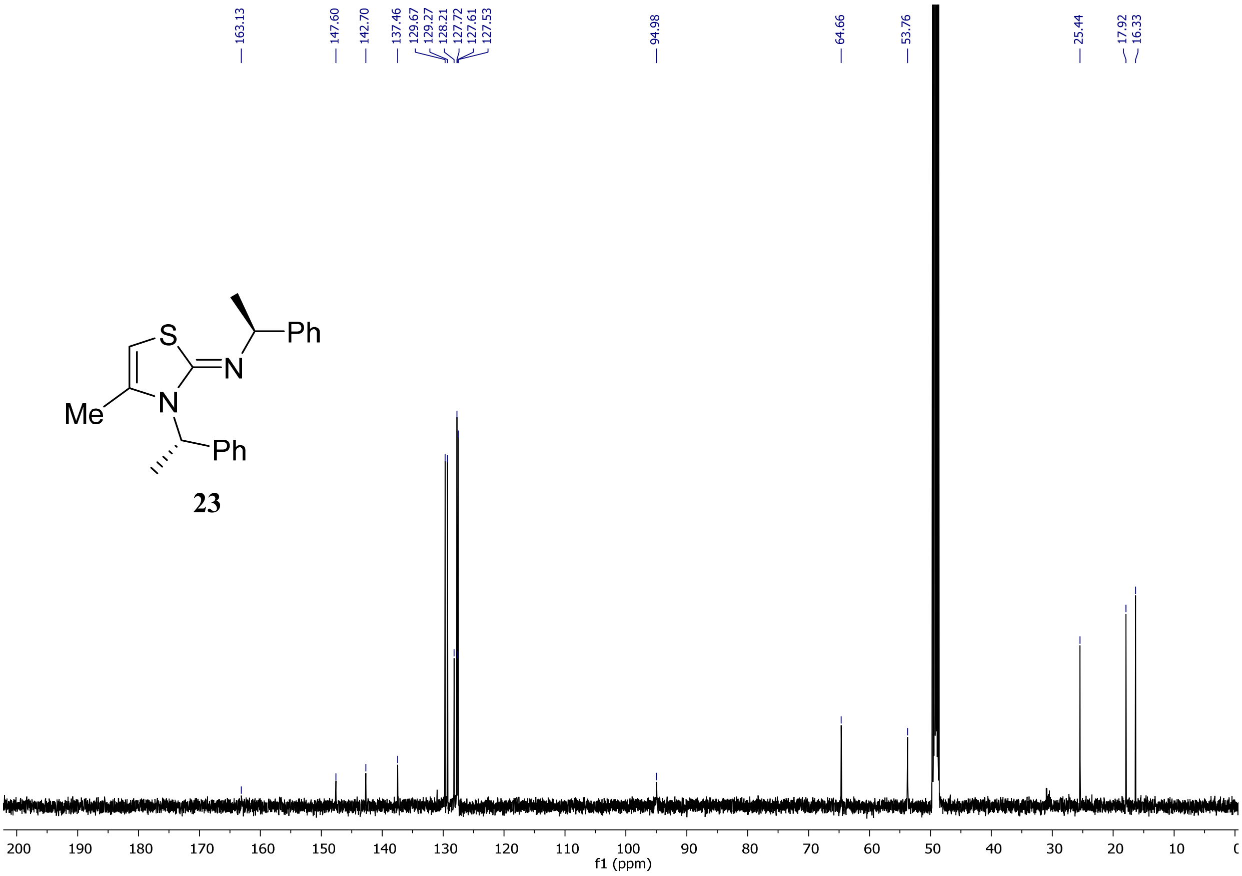

Figure S50. $^{13}C\{^{1}H\}$ Spectrum of **23** in MeOD (125 MHz)

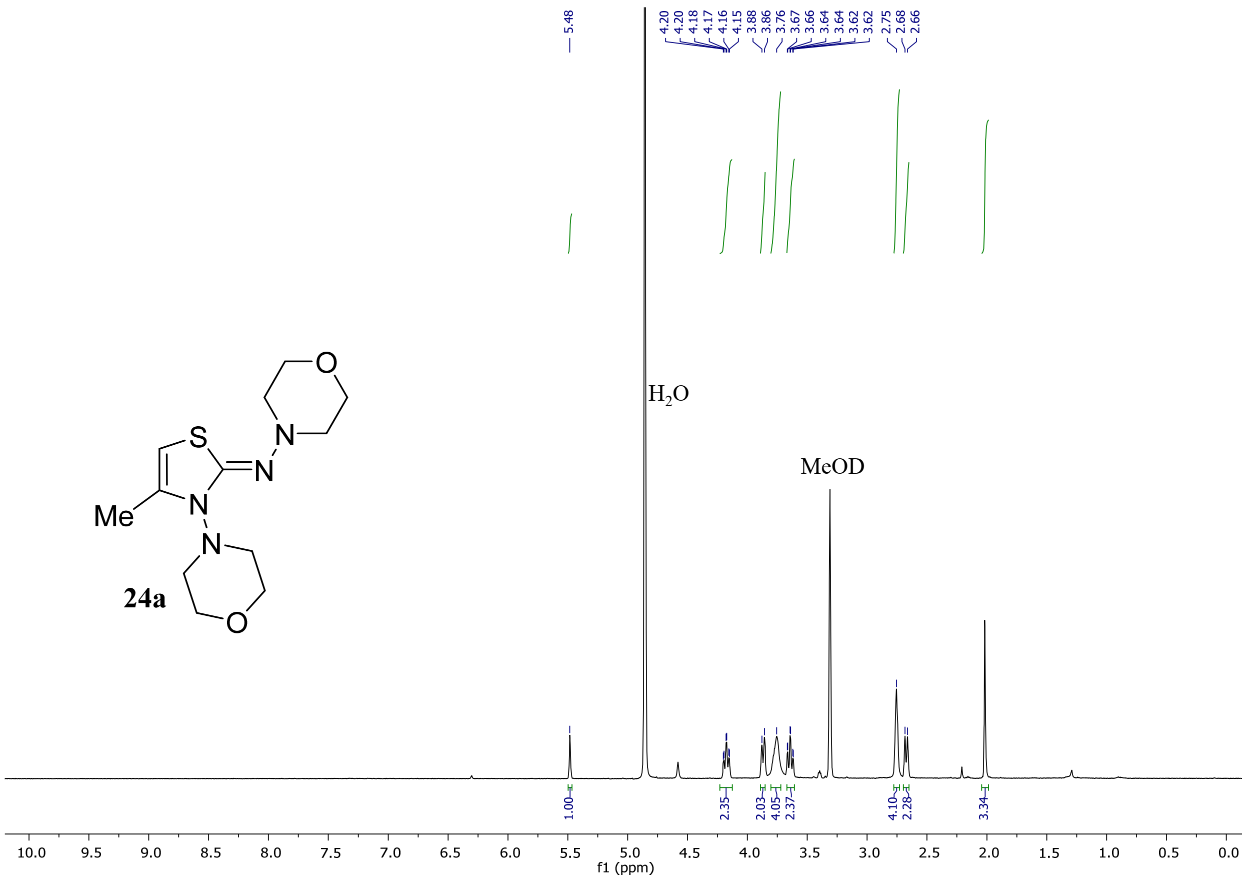

Figure S51. ^{1}H Spectrum of **24a** in MeOD (500 MHz)

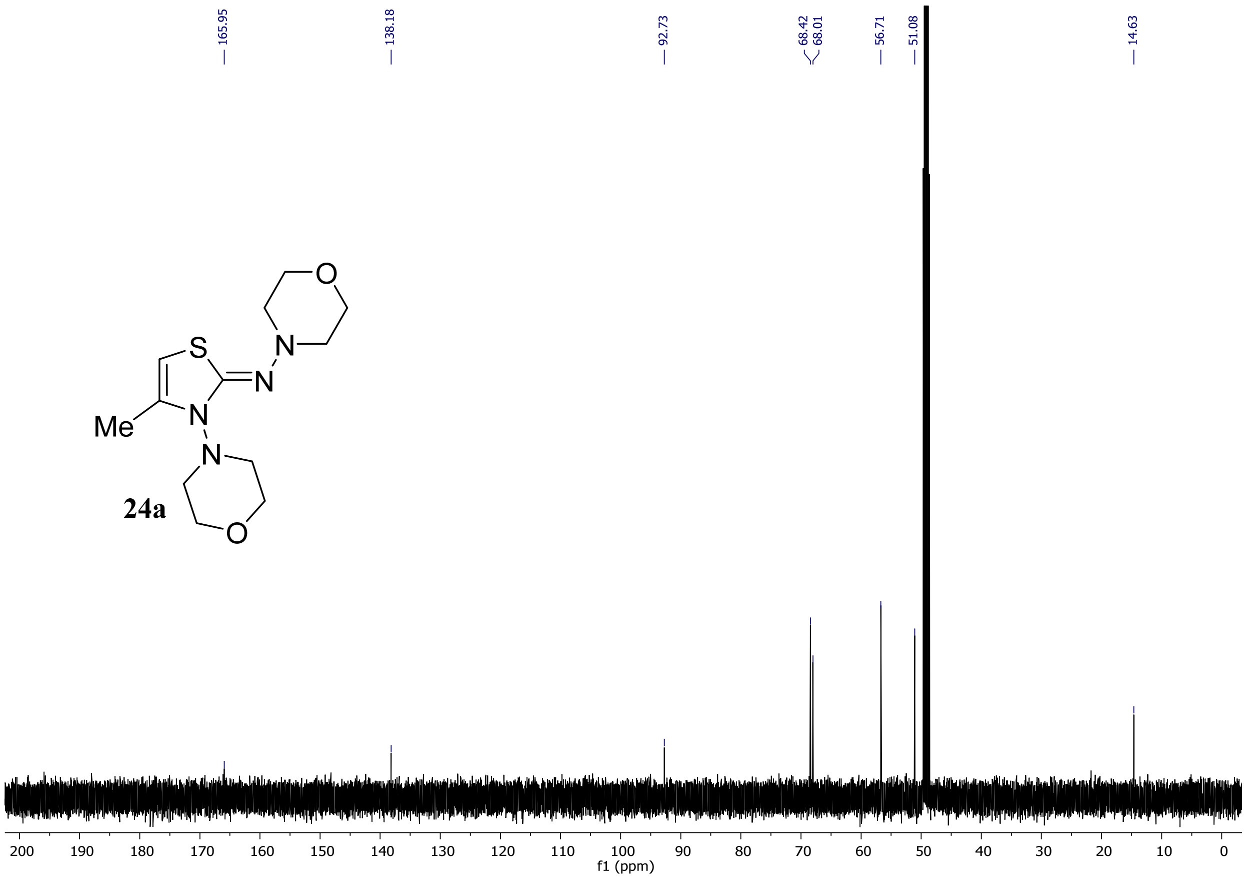

Figure S52. ^{13}C{^{1}H} Spectrum of **24a** in MeOD (125 MHz)

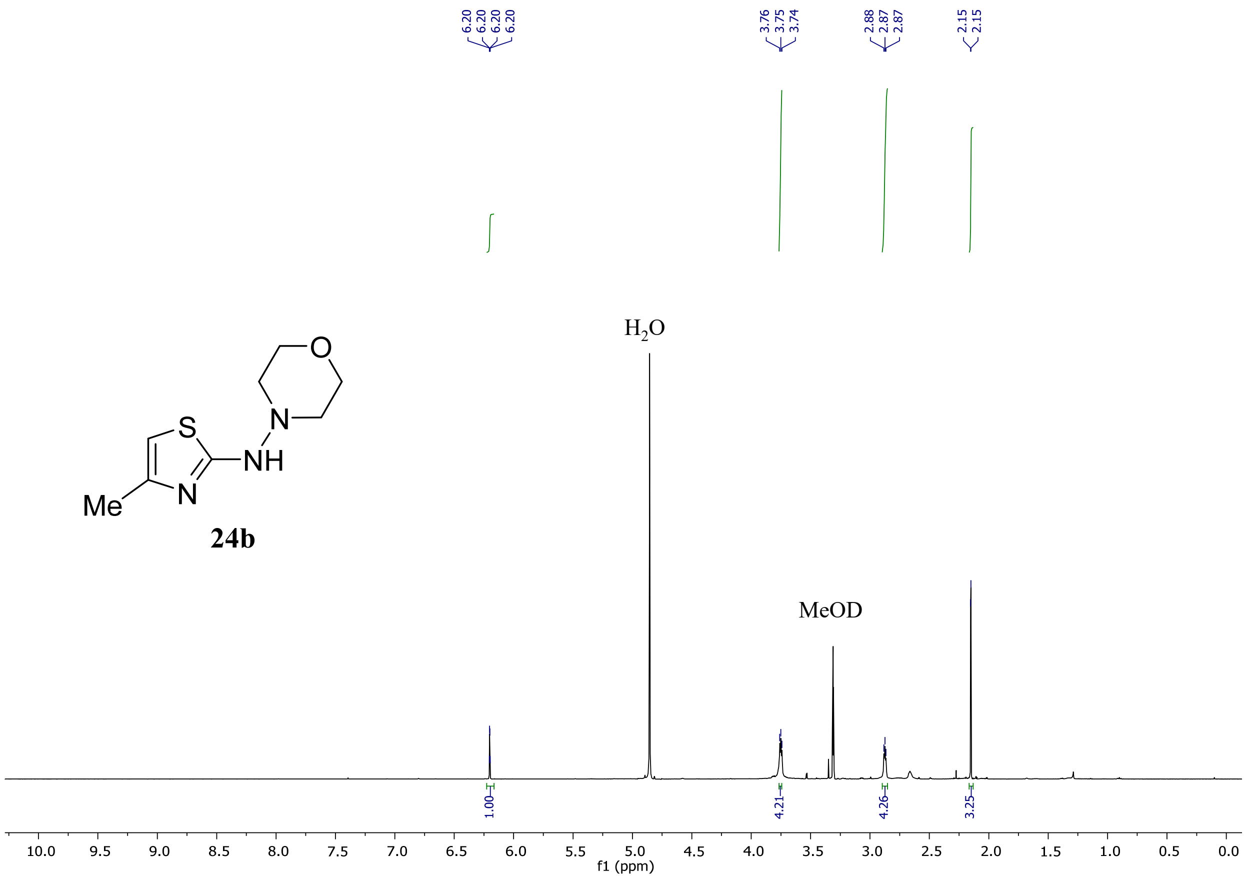

Figure S53. [1]H Spectrum of **24b** in MeOD (500 MHz)

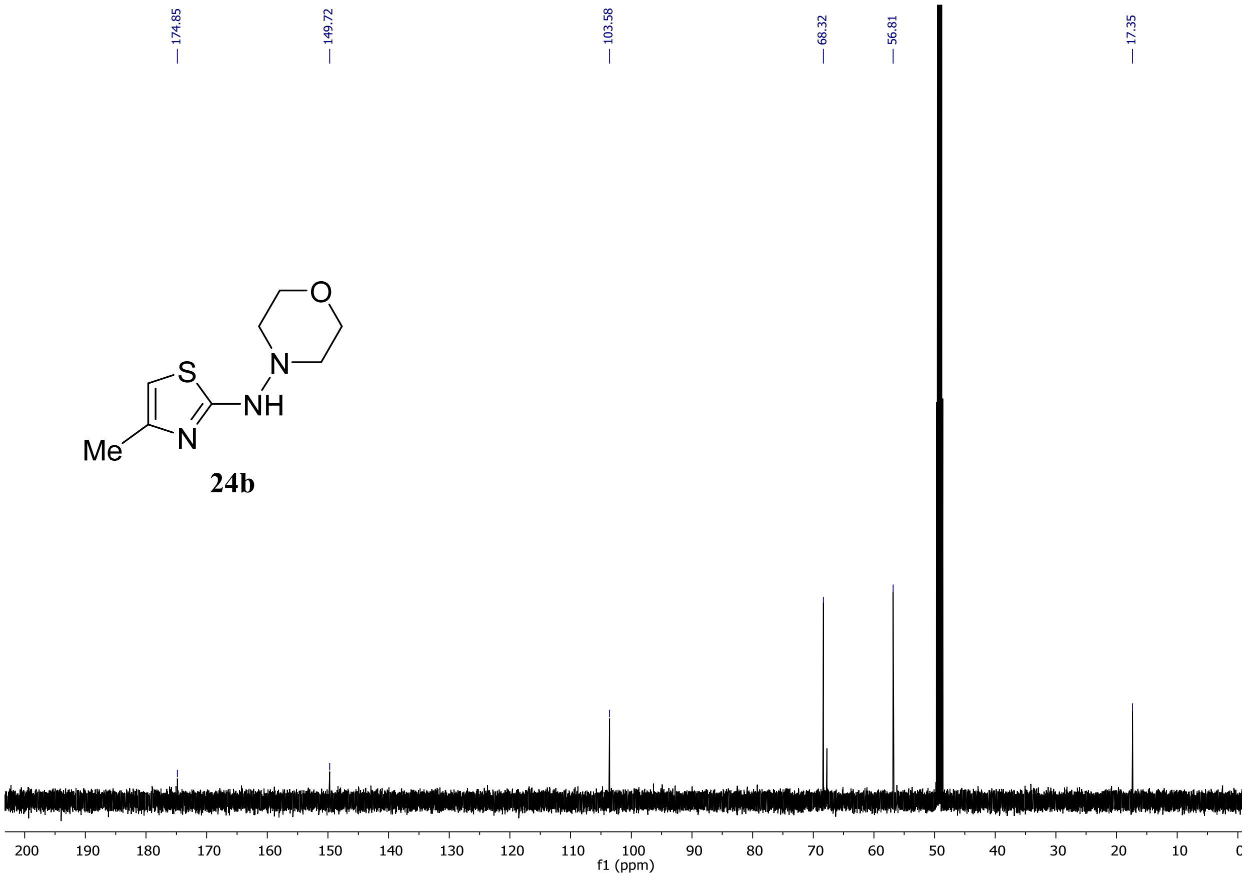

Figure S54. $^{13}C\{^{1}H\}$ Spectrum of **24b** in MeOD (125 MHz)

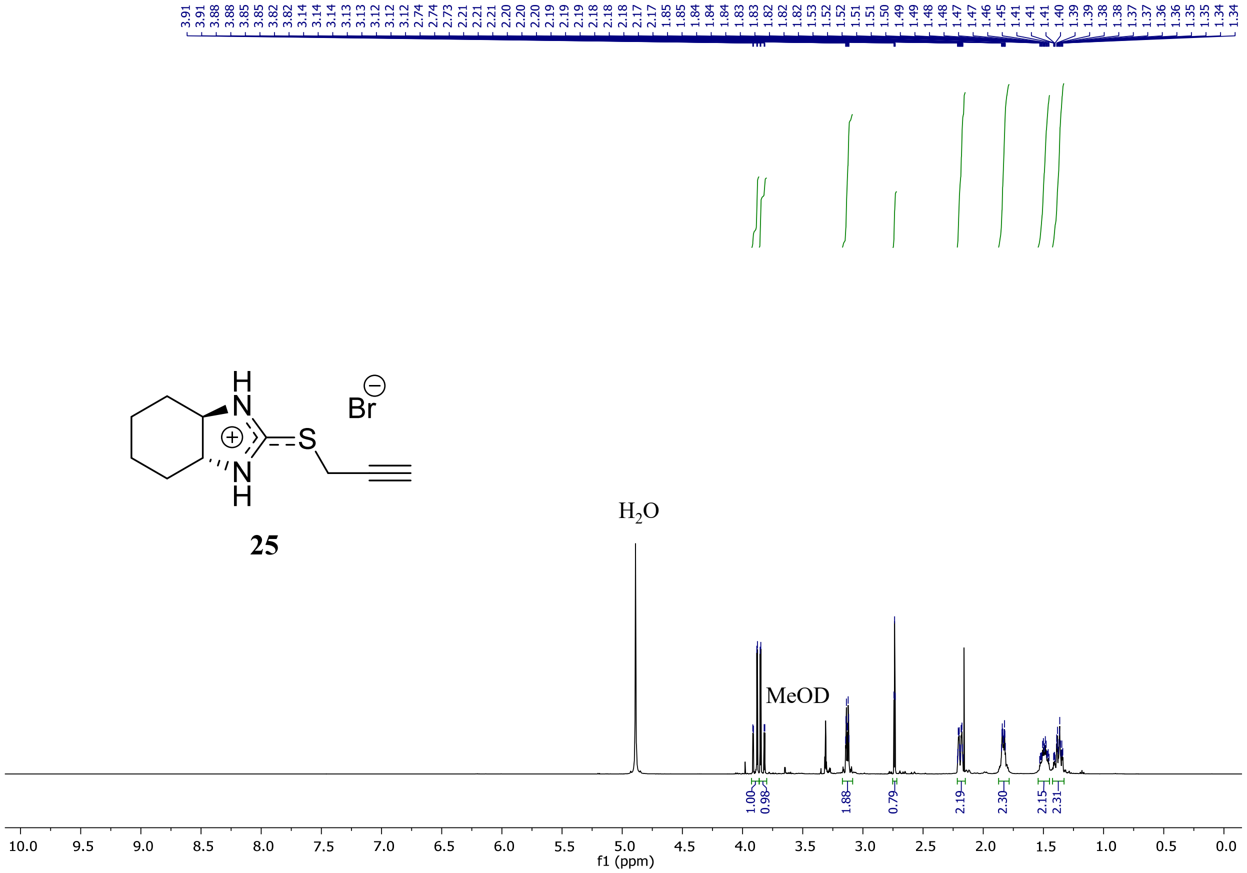

Figure S55. ^{1}H Spectrum of **25** in MeOD (500 MHz)

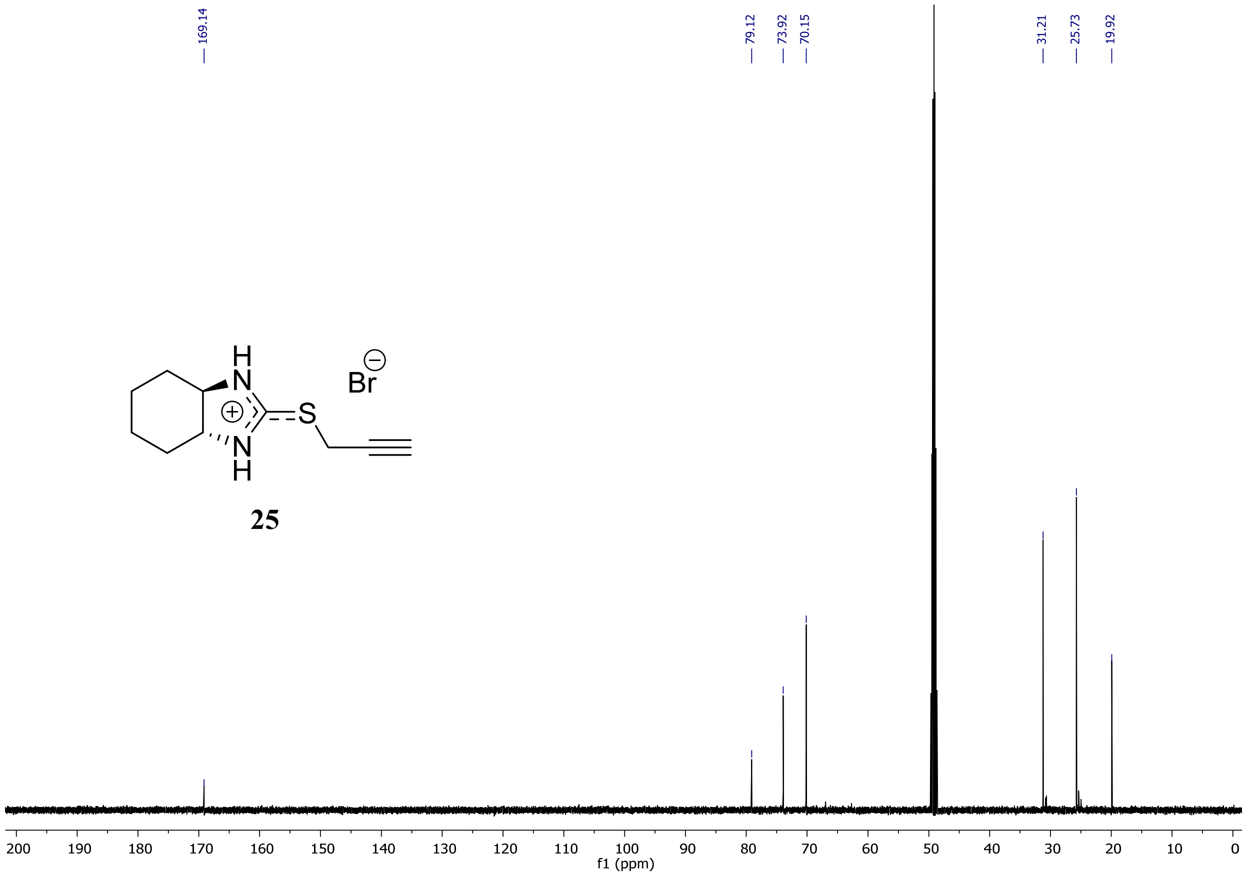

Figure S56. $^{13}C\{^{1}H\}$ Spectrum of **25** in MeOD (125 MHz)

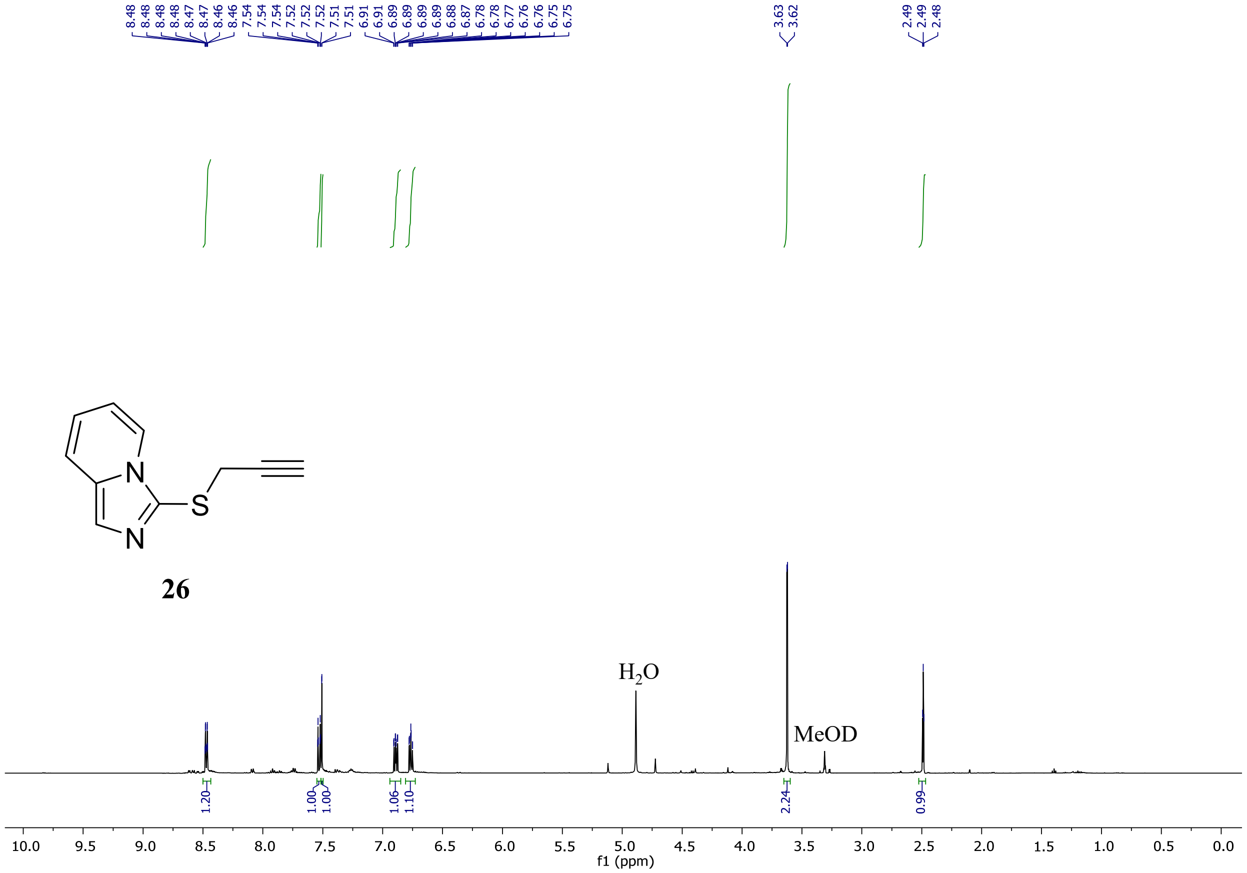

Figure S57. ^{1}H Spectrum of **26** in MeOD (500 MHz)

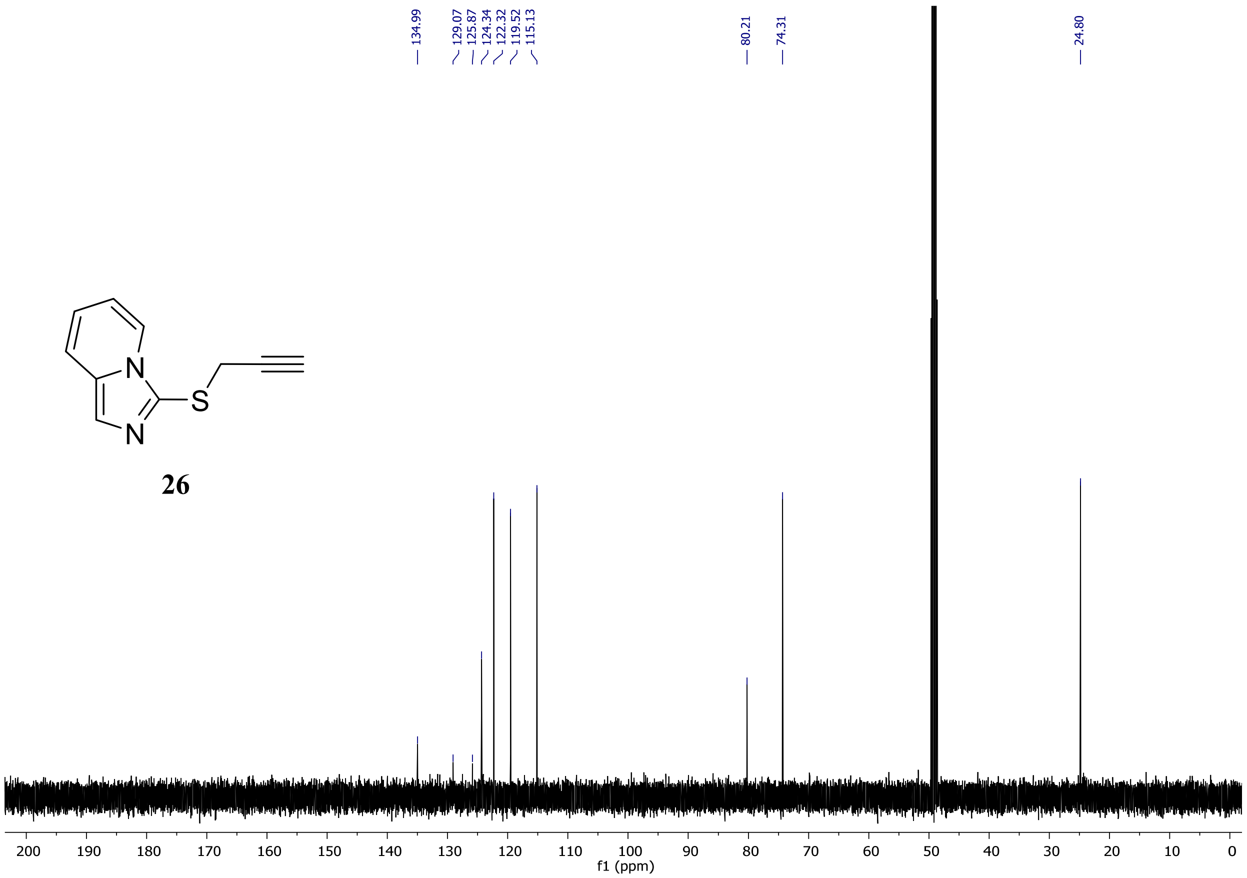

Figure S58. $^{13}C\{^{1}H\}$ Spectrum of **26** in MeOD (125 MHz)

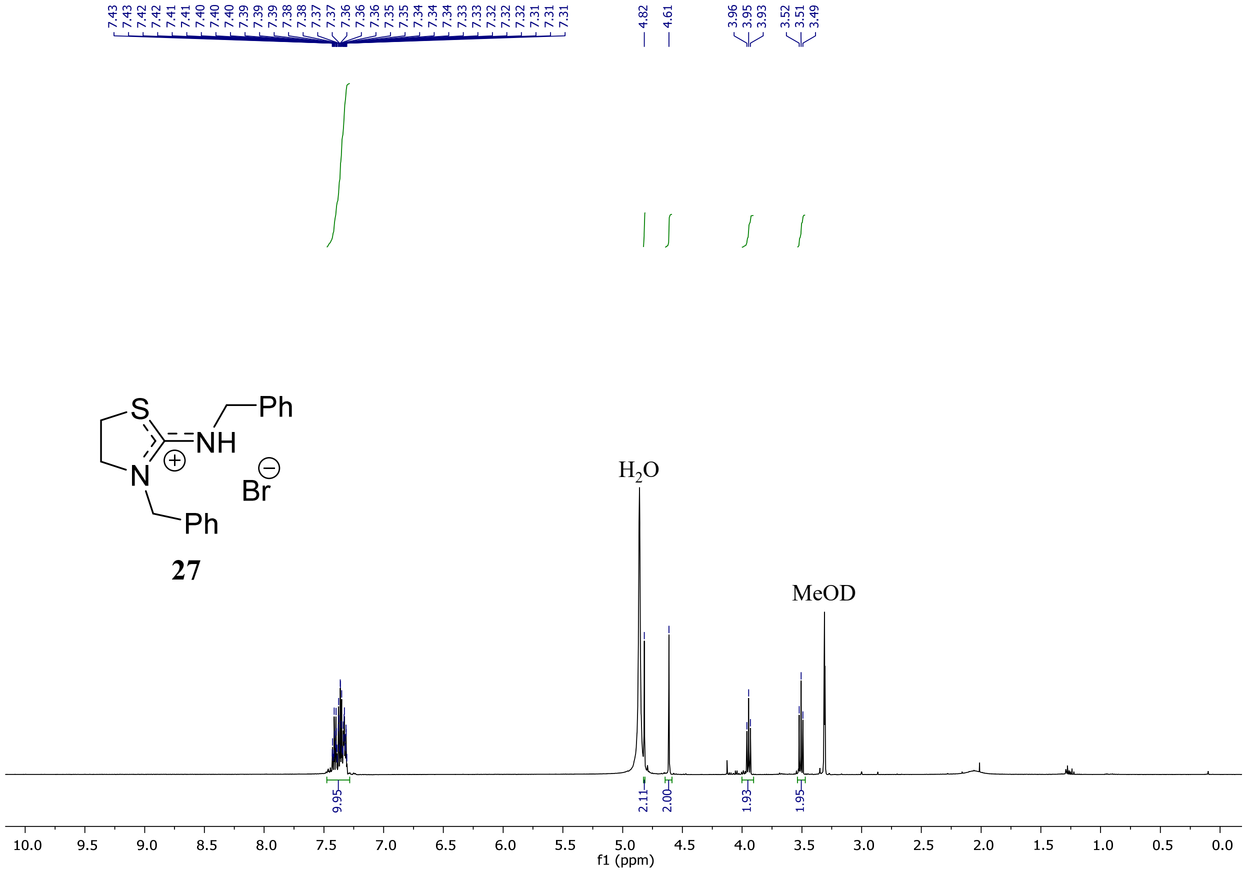

Figure S59. ^{1}H Spectrum of **27** in MeOD (500 MHz)

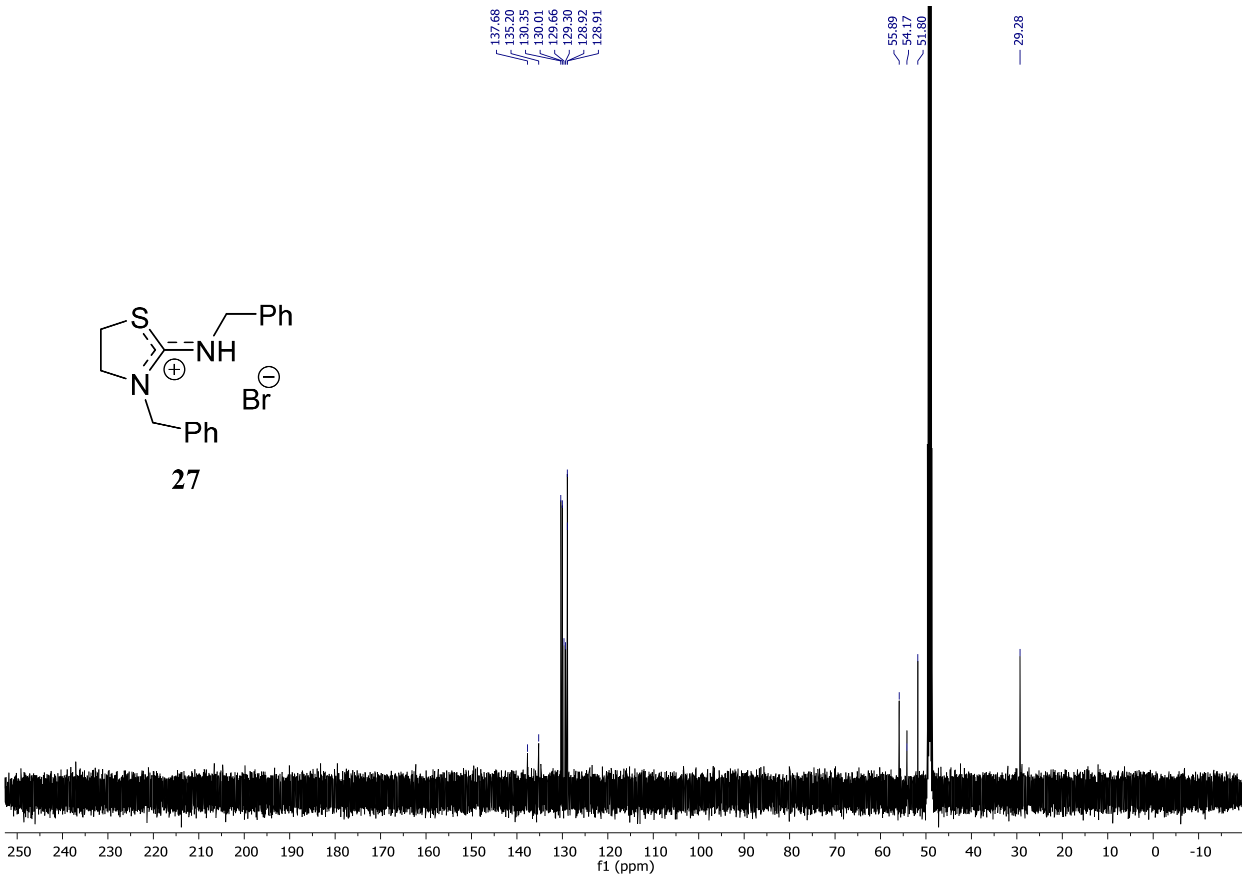

Figure S60. $^{13}C\{^{1}H\}$ Spectrum of **27** in MeOD (125 MHz)

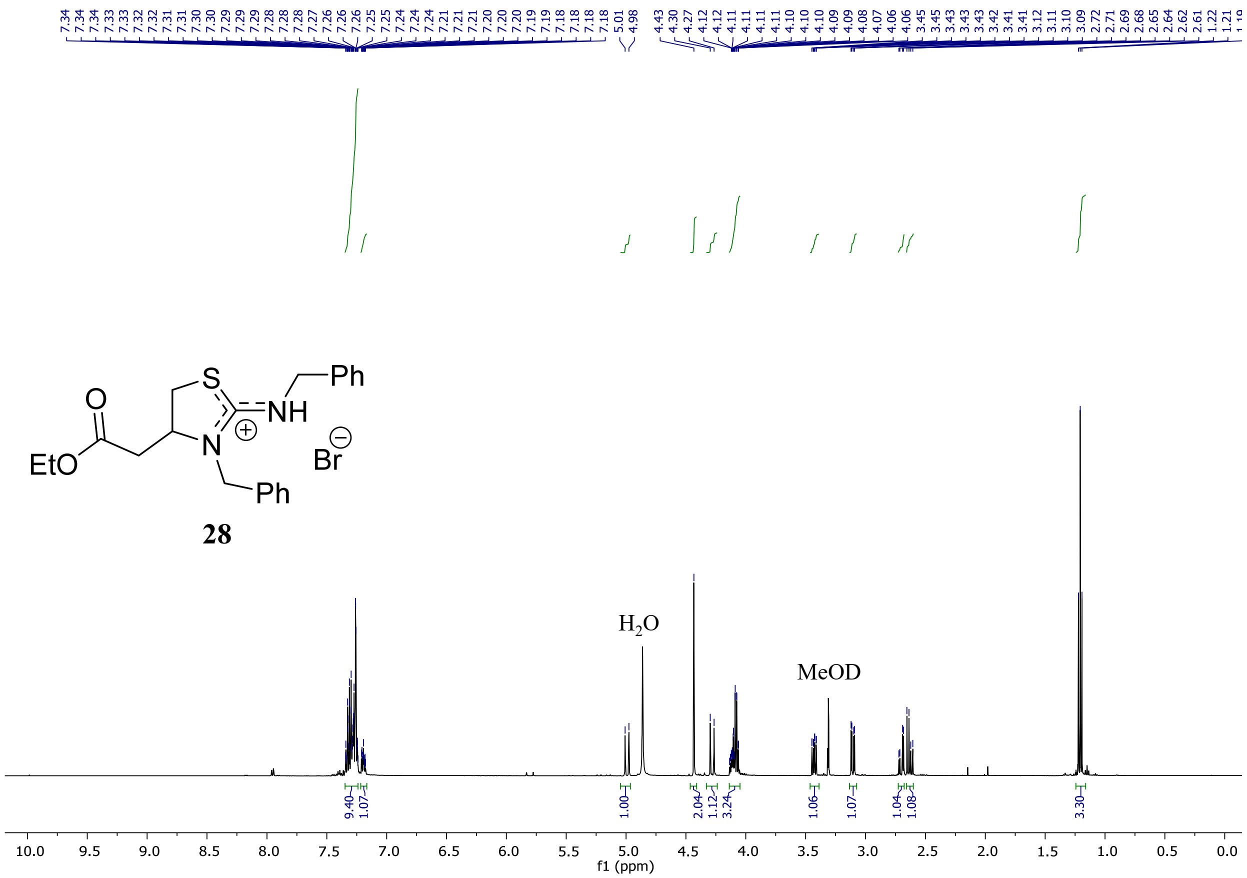

Figure S61. [1]H Spectrum of **28** in MeOD (500 MHz)

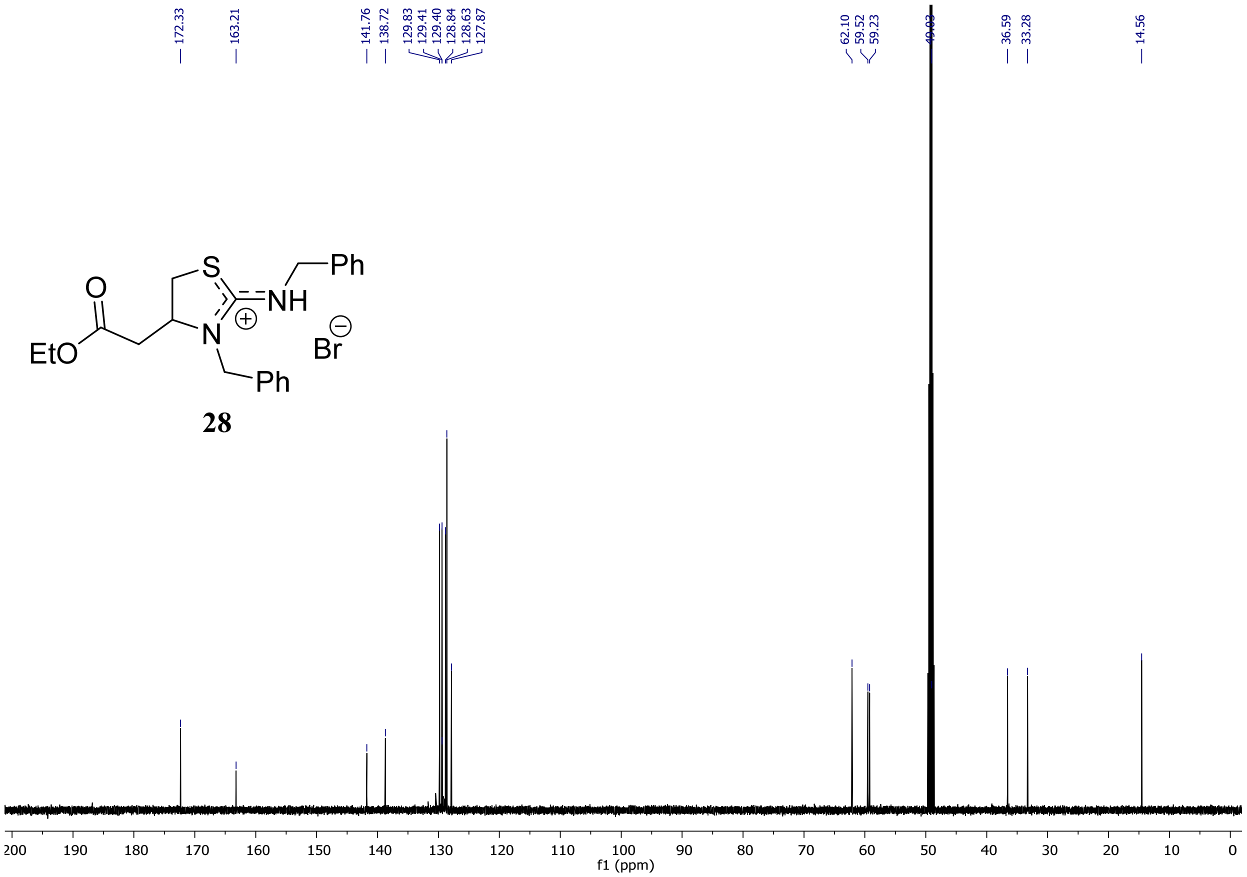

Figure S62. $^{13}C\{^1H\}$ Spectrum of **28** in MeOD (125 MHz)

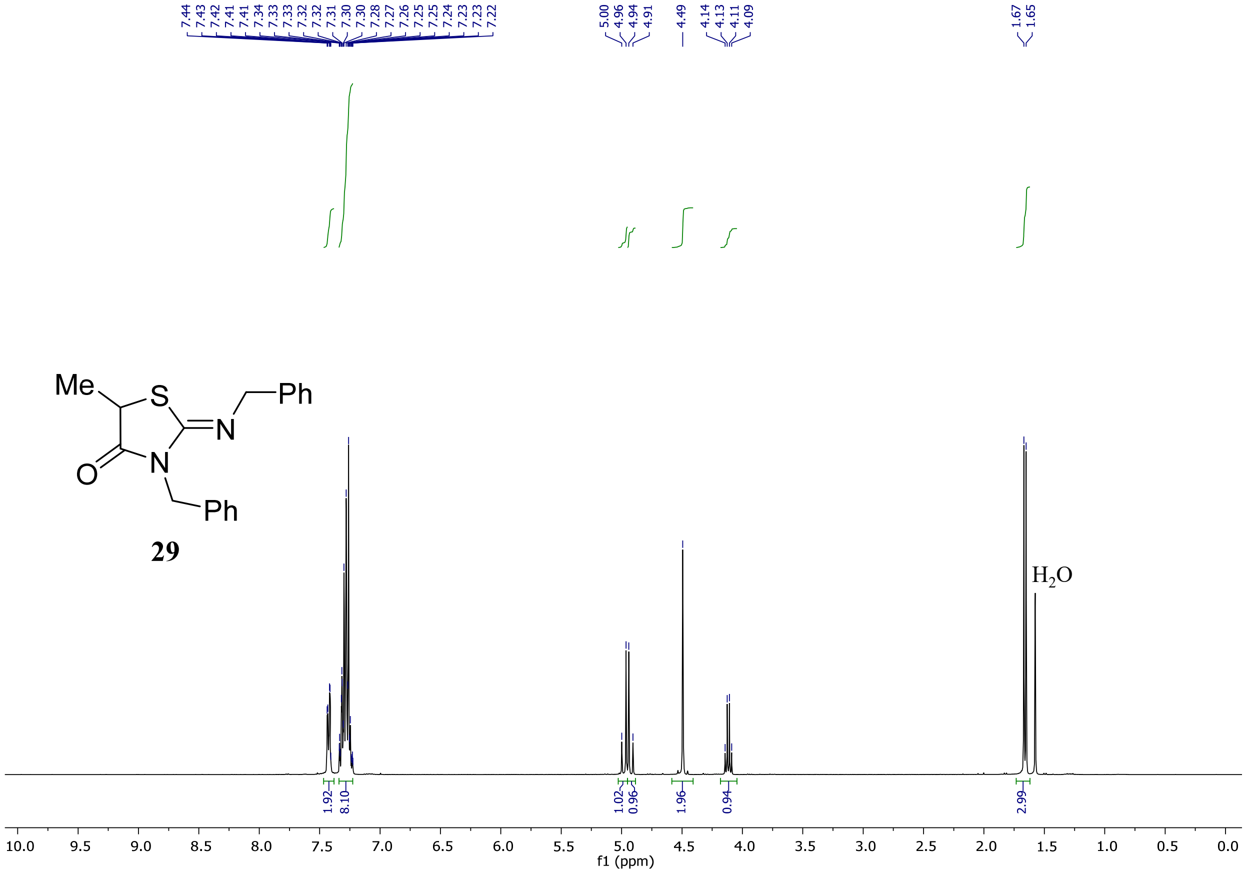

Figure S63. ^{1}H Spectrum of **29** in $CDCl_3$ (400 MHz)

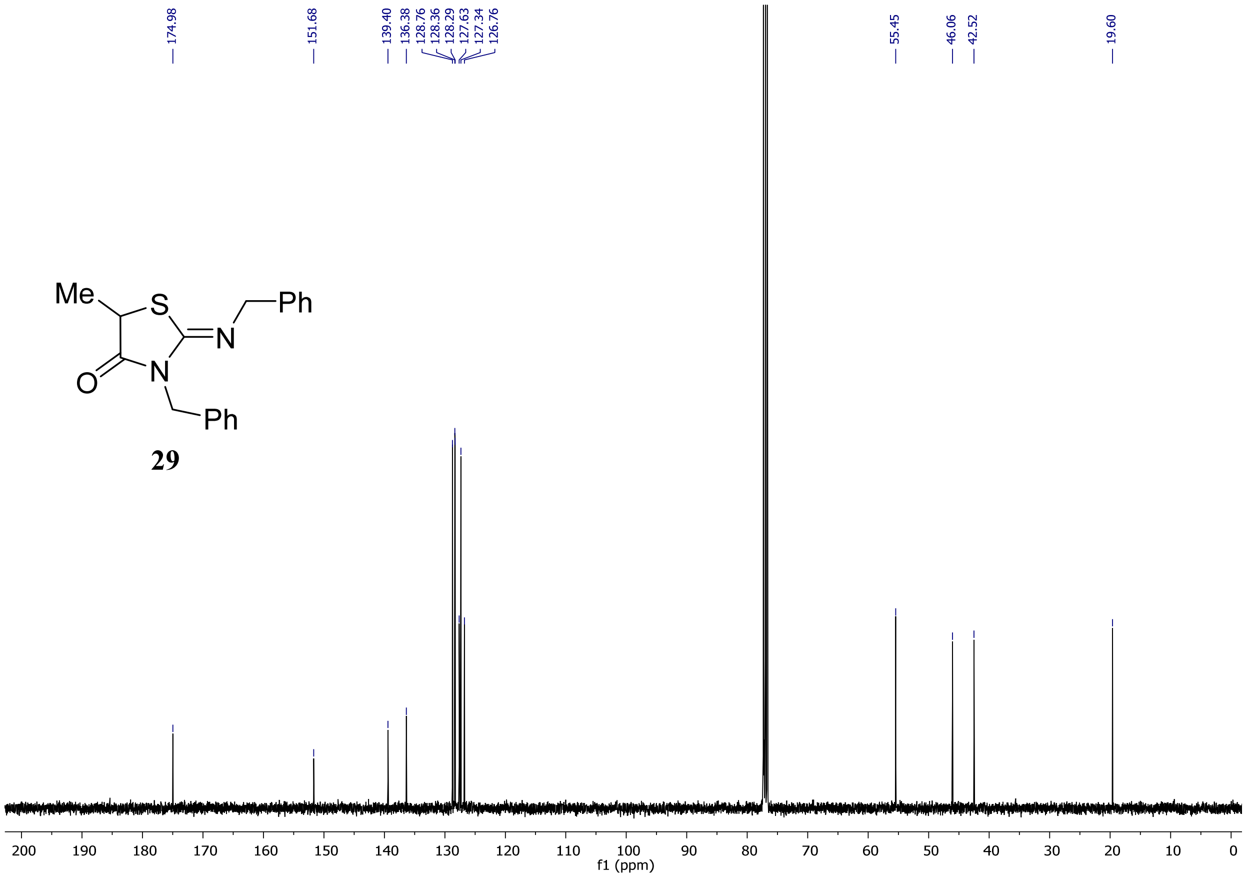

Figure S64. $^{13}C\{^1H\}$ Spectrum of **29** in $CDCl_3$ (100 MHz)

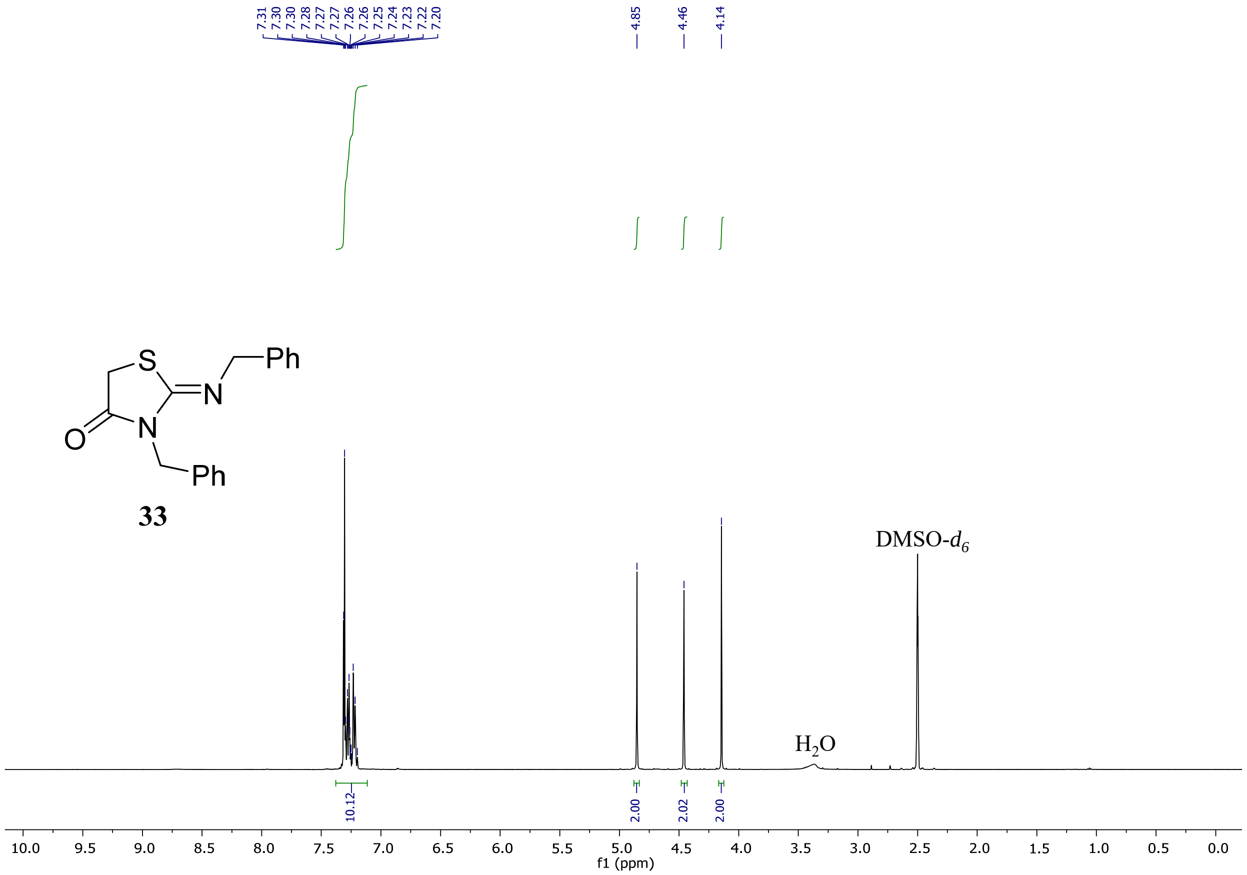

Figure S65. [1]H Spectrum of **33** in DMSO-d_6 (500 MHz)

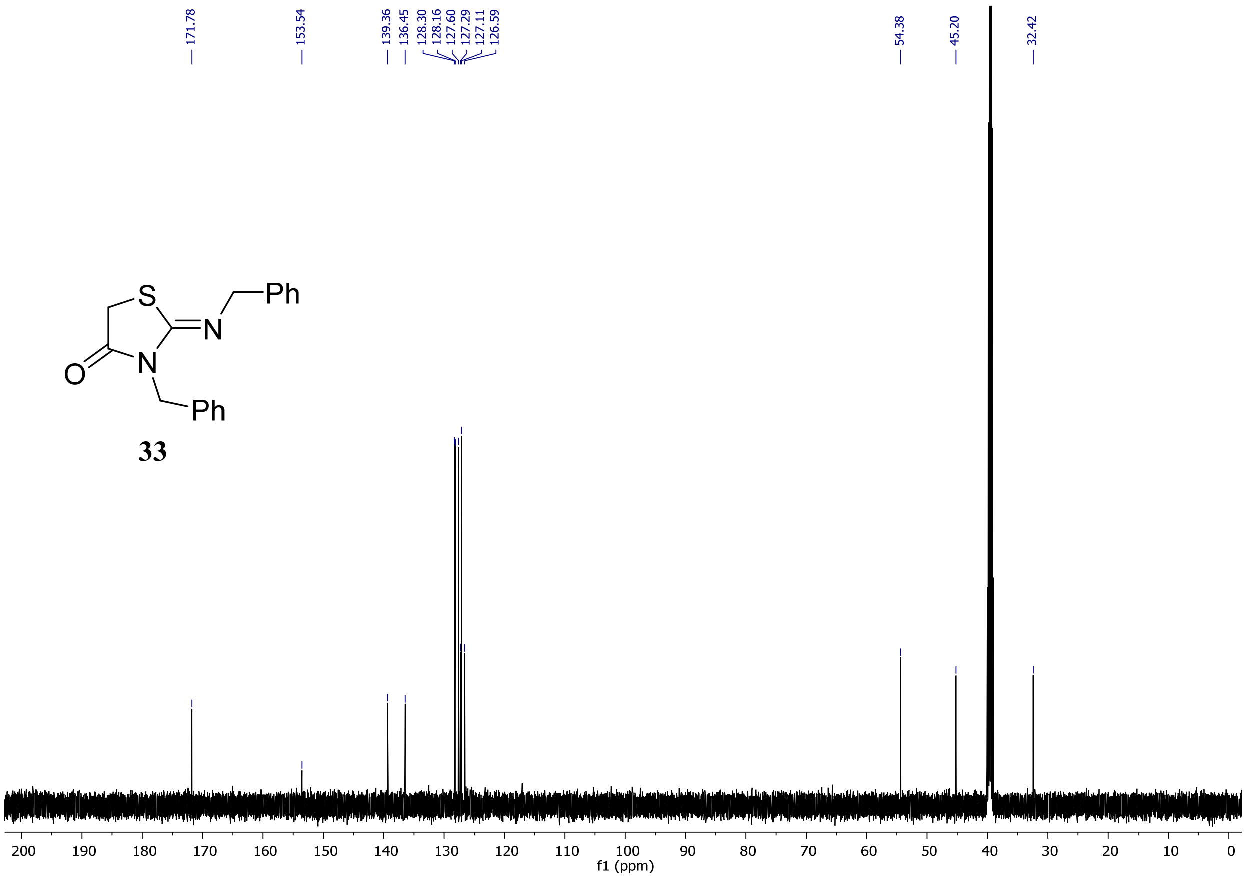

Figure S66. $^{13}C\{^{1}H\}$ Spectrum of **33** in DMSO-d_6 (125 MHz)

3. HRMS-ESI of bis-thiouronium salt 13

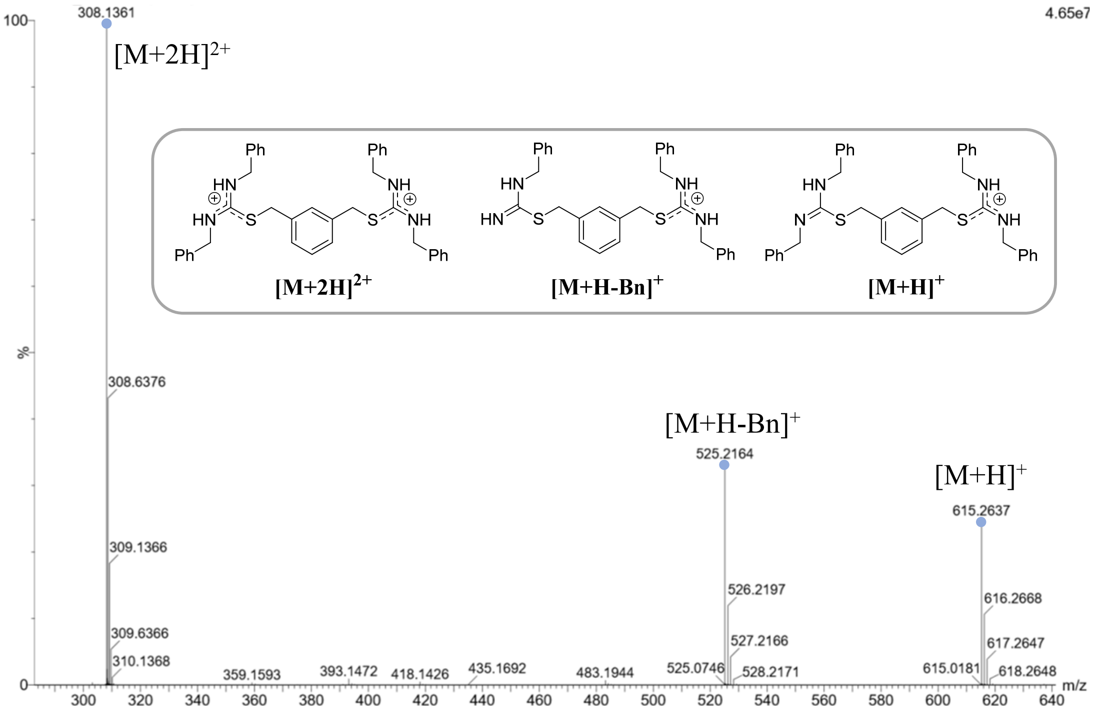

Figure S67. HRMS-ESI Spectrum of **13** (m/z).

4. DFT-Calculation Data

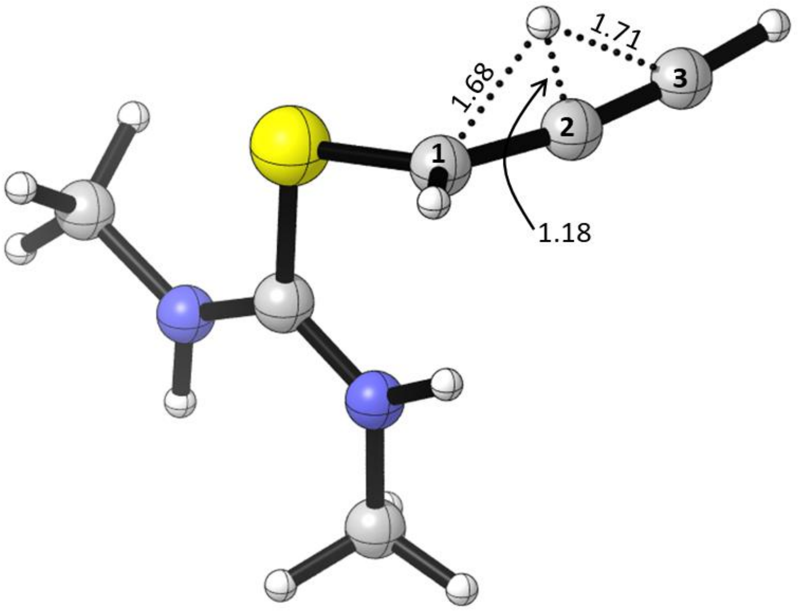

Figure S68. DFT-optimized structure of $TS_{I\text{-}III}$ (distances are given in Å). Color code: H, white; C, gray; N, blue; S, yellow.

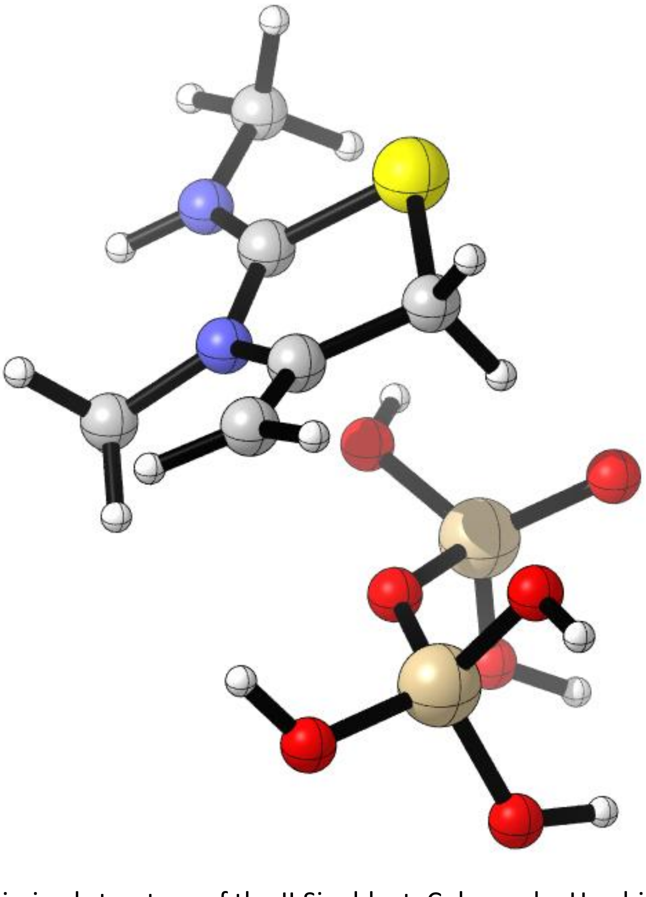

Figure S69. DFT-optimized structure of the II·Si adduct. Color code: H, white; C, gray; N, blue; S, yellow; O, red; Si, brown.

Table S4. For all the optimized species, absolute electronic energy (E), correction to Gibbs Free energy (G corr.), and Grimme's D3 dispersion correction (D3 corr.).

Species	E	G corr.	D3 corr.
**I**	-742.571255	0.131688	-0.00069816
**TS(I-II)**	-742.488178	0.123929	-0.00070358
**II**	-742.628008	0.135595	-0.00062158
**TS(II-IV)**	-742.493269	0.128388	-0.00060073
**IV**	-742.642858	0.135240	-0.00060668
**TS(I-III)**	-742.448616	0.123363	-0.00066534
**III**	-742.576519	0.129151	-0.00066359
**I·Si**	-1851.50970	0.195864	-0.00228598
**TS(I-II)·Si**	-1851.47672	0.191964	-0.00227921
**II·Si**	-1851.55257	0.200309	-0.00236407
**TS(II-IV)·Si**	-1851.52883	0.195002	-0.0023816
**IV·Si**	-1851.57467	0.200002	-0.00233928
**TS(I-A)·Si**	-1851.48046	0.188182	-0.00194218
**A·Si**	-1851.48268	0.187295	-0.00189063
**TS(A-III)·Si**	-1851.48029	0.187670	-0.00202582
**III·Si**	-1851.51236	0.191052	-0.00229039
**TS(III-IV)·Si**	-1851.48384	0.192570	-0.00231423

Cartesian coordinates of the optimized species

I
C 0.735463 0.150875 0.091275
C -2.561328 -0.108212 -0.211711
C -1.789840 -0.843027 0.783682
S -0.139957 -1.371402 0.197414
H -1.687347 -0.283074 1.717772
N 0.176640 1.320652 0.343069
N 2.008278 0.065087 -0.255934
C 0.908471 2.580254 0.249522
H 1.269588 2.739286 -0.771124
H 0.221996 3.381617 0.514402
H 1.750080 2.583908 0.948181
C 2.716104 -1.184648 -0.522616
H 2.275125 -1.707156 -1.375594
H 3.746746 -0.929723 -0.761492
H 2.706896 -1.830384 0.359256
C -3.182104 0.522012 -1.033278
H -3.739767 1.074160 -1.763677
H -2.281725 -1.790006 1.027710
H 2.536689 0.926922 -0.350339
H -0.813992 1.373187 0.545073

TS$_{I-II}$
C -0.679903 0.247135 -0.090396
C 2.388105 -0.329402 0.145897
C 1.579113 -1.152110 -0.707941
S -0.091805 -1.456164 -0.069431
H 1.567818 -0.748287 -1.724035
N 0.165458 1.186673 -0.309885
N -2.007478 0.362259 0.135818
C -0.351907 2.549315 -0.337568
H -0.782427 2.843906 0.630061
H 0.466562 3.234573 -0.564415
H -1.125490 2.674866 -1.107533
C -2.906344 -0.742227 0.441749
H -2.648888 -1.232204 1.387104
H -3.913578 -0.336105 0.529325
H -2.908226 -1.481123 -0.364714
C 2.986307 0.486367 0.867043
H 3.786143 0.798691 1.522390
H 2.095095 -2.126120 -0.732293
H -2.364044 1.299722 0.274980
H 2.097843 1.173710 0.565782

II
C -0.622263 0.206801 -0.027711
C 1.665703 -0.165587 0.007963
C 1.180793 -1.566742 -0.250345
S -0.623801 -1.536183 0.054077
H 1.345365 -1.855610 -1.291147
N 0.593884 0.753514 -0.130586
N -1.741563 0.889172 0.031333
C 0.817126 2.195732 -0.136937
H 0.899673 2.576135 0.885122
H 1.737178 2.395953 -0.685427
H -0.000313 2.692864 -0.660319
C -3.062041 0.264203 0.094236
H -3.170289 -0.314430 1.015772
H -3.803072 1.061059 0.086668
H -3.219107 -0.378469 -0.775435
C 2.917769 0.178970 0.299350
H 3.669935 -0.597215 0.384426
H 1.648761 -2.290895 0.415657
H -1.697662 1.902027 0.084793
H 3.221588 1.208453 0.450091

TS$_{II-IV}$
C 0.646503 0.185187 -0.022019
C -1.638760 -0.209912 -0.083731
C -1.117816 -1.535199 0.011358
S 0.639027 -1.542751 -0.043766
H -2.003471 -0.742828 1.019204
N -0.589813 0.727334 -0.054567
N 1.752731 0.888386 0.044481
C -0.808552 2.172063 -0.076480
H -1.780683 2.363541 -0.527067
H -0.773562 2.581522 0.935869
H -0.050223 2.640308 -0.705567
C 3.076208 0.270523 0.059742
H 3.242531 -0.294987 -0.861274
H 3.813893 1.067238 0.127611
H 3.177246 -0.387611 0.928081
C -2.989201 0.142184 0.063824
H -3.740177 -0.621290 -0.091957
H -3.276556 1.147866 0.332065
H -1.679216 -2.428401 -0.222980
H 1.695066 1.899538 0.120711

IV
C 0.664476 0.183218 0.000028
C -1.590234 -0.279275 -0.000001
C -1.094247 -1.529961 0.000028
S 0.651857 -1.546426 0.000042
H -3.266724 0.714867 0.888114
N -0.574917 0.692618 -0.000096
N 1.775089 0.901779 0.000329
C -0.795074 2.139098 0.000036
H -1.864151 2.335698 -0.001305
H -0.351501 2.577841 0.897369

H -0.349331 2.578302 -0.895991
C 3.097115 0.287757 -0.000214
H 3.235430 -0.326340 -0.895714
H 3.836977 1.085842 -0.000965
H 3.236472 -0.325616 0.895626
C -3.024739 0.123428 -0.000083
H -3.642979 -0.775124 0.000215
H -3.266691 0.714392 -0.888615
H -1.648638 -2.457421 0.000092
H 1.706436 1.914010 0.000112

$TS_{I\text{-}III}$

C -0.863192 0.120422 -0.095559
C 2.643712 0.129764 0.180942
C 1.755530 -0.310219 -0.829096
S 0.311030 -1.184254 -0.423316
H 2.010905 -0.144751 -1.867533
N -0.522539 1.379889 -0.269256
N -2.066419 -0.229647 0.320694
C -1.424197 2.498622 -0.027511
H -1.750083 2.507893 1.016787
H -0.880995 3.417714 -0.238710
H -2.296417 2.439098 -0.685891
C -2.492813 -1.609344 0.544870
H -1.878767 -2.083926 1.314309
H -3.525711 -1.580508 0.886400
H -2.443978 -2.187830 -0.381259
C 3.514352 0.331752 1.032494
H 4.311468 0.418212 1.746035
H 3.136026 -0.916132 -0.077604
H -2.750465 0.501973 0.488020
H 0.413893 1.558642 -0.624411

III

C 0.890881 0.118115 0.037769
C -2.736666 0.010584 0.018138
C -1.713742 -0.180548 0.814846
S -0.322917 -1.145707 0.262611
N 0.583322 1.396075 0.145759
N 2.117739 -0.281434 -0.245247
C 1.555549 2.470387 -0.024800
H 1.964217 2.463415 -1.039931
H 1.039903 3.413234 0.145637
H 2.365973 2.369041 0.702763
C 2.529872 -1.678689 -0.359195
H 2.012721 -2.172126 -1.186332
H 3.599588 -1.687571 -0.558922
H 2.340264 -2.213338 0.574891
C -3.760025 0.192563 -0.766364
H -3.831481 1.082857 -1.386549
H -1.703810 0.151879 1.849929
H 2.822915 0.426915 -0.425161
H -0.387044 1.643625 0.309316
H -4.559224 -0.543584 -0.813369

I·Si

C 3.049165 -0.330458 -0.368008
C 0.708473 0.618383 1.721963
C 1.560138 1.575821 1.028602
S 3.270792 0.975995 0.793328
H 1.127854 1.871600 0.069700
N 1.916441 -0.543166 -0.999473
N 4.116343 -1.093400 -0.579027
C 1.764780 -1.644836 -1.943856
H 1.914454 -2.607713 -1.444688
H 0.750491 -1.601538 -2.337910
H 2.469338 -1.547435 -2.776545
C 5.402984 -0.941771 0.093474
H 5.292350 -1.059221 1.174427
H 6.058971 -1.726058 -0.280226
H 5.854427 0.028485 -0.131943
C 0.001148 -0.180371 2.287858
H -0.646141 -0.881319 2.777106
H 1.689196 2.483785 1.626181
H 4.046012 -1.824580 -1.278273
H 1.064714 0.058955 -0.903849
H -0.909090 -1.012644 -0.761792
Si -1.706704 1.412028 -0.649752
O -2.738050 0.445282 0.247871
O -1.332750 2.646167 0.397510
H -1.182889 3.509032 -0.010014
O -2.608556 2.161973 -1.834685
H -2.597294 1.723341 -2.695398
O -0.502400 0.538537 -1.226099
Si -2.756133 -1.189620 0.363528
O -1.408967 -1.783832 -0.356971
O -4.042261 -1.864084 -0.420971
H -4.922743 -1.718226 -0.049879
O -2.849160 -1.535639 1.973817
H -3.155047 -2.417985 2.222515

$TS_{I\text{-}II}$·Si

C -2.992252 -0.068404 0.209929
C -0.795994 -1.472852 -0.145969
C -1.053159 -0.754654 -1.423038
S -2.800747 -0.239544 -1.542950
H -0.430759 0.139517 -1.510698
N -1.920684 -0.289966 0.914936
N -4.212018 0.199452 0.678923
C -2.024127 -0.535049 2.341838
H -2.823878 -1.249058 2.575203
H -1.072926 -0.959802 2.679035
H -2.201582 0.394777 2.894964
C -5.393008 0.387934 -0.153835

H -5.622035 -0.520287 -0.718376
H -6.232017 0.609945 0.503946
H -5.259899 1.226455 -0.844306
C -0.182612 -2.431539 0.388711
H -0.042777 -2.836441 1.379767
H -0.845974 -1.409280 -2.272142
H -4.306088 0.348629 1.677320
H -0.404903 0.780135 0.970154
H 1.711255 -0.599316 1.805661
Si 1.398675 1.729398 -0.046429
O 2.086196 0.413297 -0.748436
O 0.600716 2.452642 -1.280522
H 0.162780 3.298466 -1.114327
O 2.511287 2.768239 0.562147
H 2.907782 2.571181 1.422122
O 0.429227 1.253015 1.191545
Si 2.861315 -0.896639 -0.109318
O 2.579427 -0.918131 1.512756
O 4.488705 -0.745493 -0.236451
H 4.907949 -1.008756 -1.066404
O 2.407537 -2.250405 -0.908912
H 1.476222 -2.516429 -0.714120

II·Si

C -2.455359 -0.400020 -0.190734
C -0.714309 -1.866091 0.220181
C -0.844547 -1.230050 1.574622
S -2.459258 -0.364930 1.552976
H -0.829821 -1.961840 2.381767
N -1.475638 -1.134184 -0.722340
N -3.376411 0.218216 -0.891316
C -1.268811 -1.238964 -2.160656
H -1.437033 -0.262768 -2.621054
H -0.230553 -1.519649 -2.336476
H -1.935380 -1.986728 -2.600554
C -4.358129 1.122133 -0.298677
H -3.856928 1.957145 0.198658
H -4.983468 1.505786 -1.102469
H -4.990607 0.587199 0.415224
C 0.011294 -2.943434 -0.076890
H 0.564911 -3.443353 0.709711
H -0.067455 -0.474355 1.728683
H -3.368868 0.120188 -1.901582
H 0.061423 -3.347080 -1.083453
H 4.111058 1.076632 0.518808
Si 0.689944 1.831966 0.051596
O 1.270859 0.350762 -0.492984
O -0.780003 1.843984 -0.746153
H -1.339693 2.607822 -0.555924
O 1.617693 2.984360 -0.746367
H 2.168108 3.513657 -0.155401
O 0.686772 1.990514 1.618898
Si 2.592417 -0.496782 -0.070925
O 3.987993 0.379521 -0.139610
O 2.888862 -1.684819 -1.170920
H 2.148620 -2.269426 -1.383415
O 2.334297 -1.074118 1.454177
H 3.072478 -1.528214 1.882650

TS_{II-IV}·Si

C 2.388542 -0.447971 0.407254
C 0.925149 -1.726401 -0.880812
C 1.576613 -0.972492 -1.871779
S 2.909163 -0.032122 -1.178259
H 1.718791 -1.337659 -2.880484
N 1.334137 -1.276686 0.406491
N 2.961512 0.023370 1.503467
C 0.707379 -1.700638 1.652978
H 0.576861 -0.835630 2.308717
H -0.278682 -2.101098 1.428904
H 1.313091 -2.464983 2.148744
C 4.019018 1.021274 1.453432
H 3.662577 1.940401 0.975871
H 4.322930 1.242621 2.474906
H 4.883734 0.635480 0.904418
C -0.054501 -2.707244 -1.022760
H -0.179568 -3.112801 -2.023062
H 0.273935 0.417870 -1.903167
H 2.571523 -0.218968 2.406806
H -0.266577 -3.379231 -0.198035
H -1.631530 -1.824403 -1.052734
Si -0.632784 1.688677 -0.240735
O -1.374713 0.499589 0.615649
O 0.686388 2.033408 0.674548
H 1.305793 2.694150 0.335577
O -1.570352 3.038960 -0.331924
H -2.250928 3.059812 -1.018964
O -0.304029 1.223187 -1.772744
Si -2.697158 -0.434715 0.328169
O -2.525872 -1.337923 -1.015539
O -4.027935 0.490095 0.033678
H -4.345378 1.055820 0.750479
O -2.825672 -1.303847 1.722725
H -3.509255 -1.986603 1.759365

IV·Si

C 2.400150 -0.266913 -0.311879
C 1.092151 -1.504292 1.118296
C 0.736781 -2.052316 -0.057711
S 1.566003 -1.314239 -1.403723
H -0.073652 -2.738843 2.373052
N 2.037114 -0.477270 0.957690
N 3.306558 0.620674 -0.699385
C 2.547661 0.367624 2.034488

H 2.302595 1.410662 1.817613
H 2.070362 0.079636 2.967884
H 3.629652 0.238883 2.124913
C 3.522364 0.917276 -2.108747
H 2.608854 1.304380 -2.574418
H 4.307438 1.668221 -2.179577
H 3.852397 0.018471 -2.638059
C 0.617614 -1.900854 2.473729
H 0.096262 -1.072334 2.962104
H 0.033083 -2.854063 -0.224826
H 3.616732 1.308726 -0.021250
H -1.722198 -0.270583 1.524169
Si -0.842517 1.713158 0.333071
O -1.585260 0.719855 -0.806143
O -1.573662 3.207628 0.188427
H -2.514501 3.241311 0.404628
O 0.704568 2.013420 -0.193358
H 0.783367 2.585970 -0.967849
O -0.900464 1.017557 1.759441
Si -2.503358 -0.601702 -0.478525
O -2.295443 -1.010918 1.089080
H 1.456939 -2.210934 3.104466
O -2.091954 -1.902313 -1.410323
H -2.460785 -1.927405 -2.303213
O -4.053523 -0.151834 -0.850078
H -4.740447 -0.828798 -0.787260
